# Supplementary figures and images for: Specific and redundant roles for Gli2 and Gli3 in establishing cell fate during murine hair follicle development
Source: EMBO J. 2025 Aug 26;44(19):5290–314. doi: 10.1038/s44318-025-00519-9 (PMC12488920; doi:10.1038/s44318-025-00519-9)

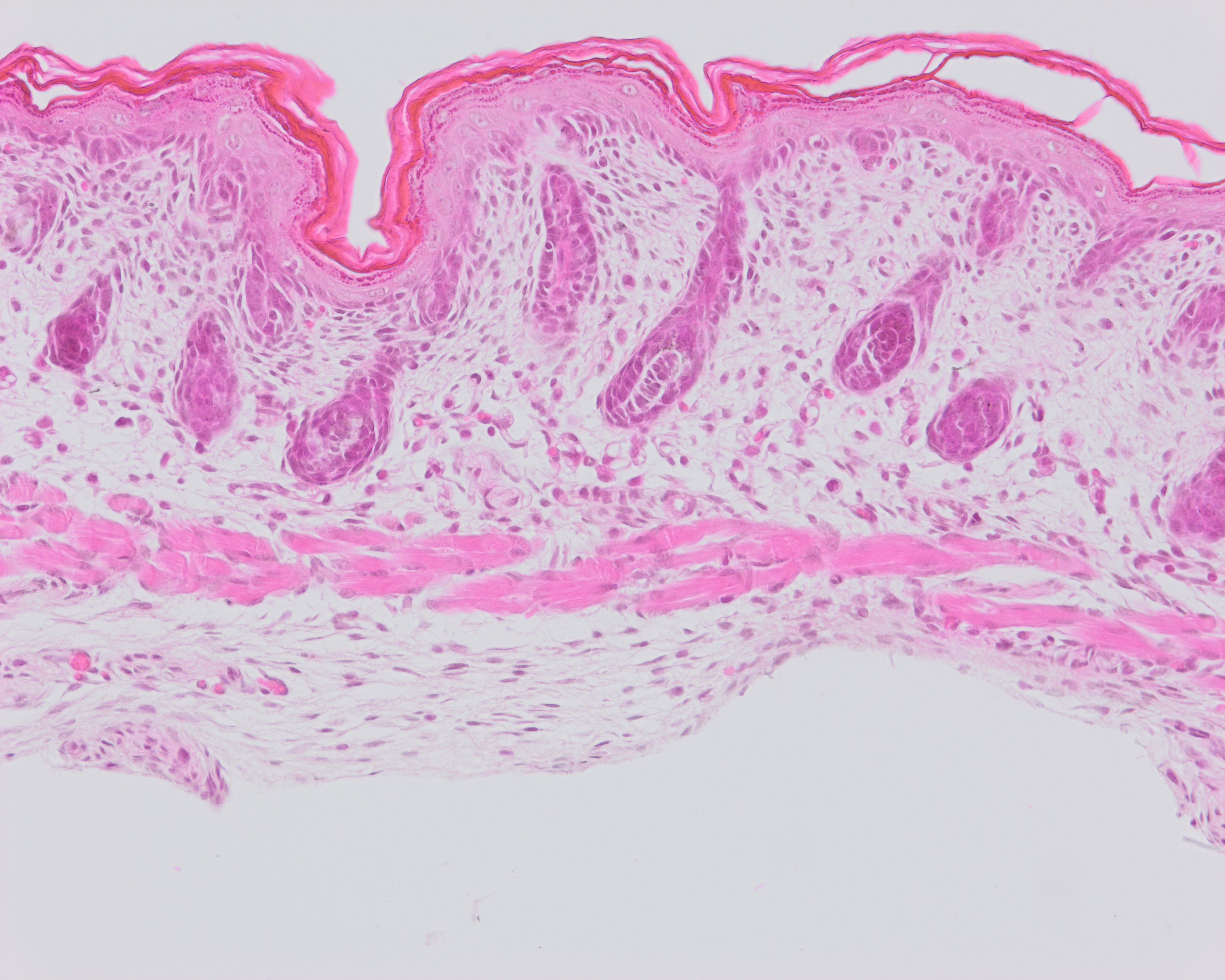

Supplement: Supplementary file 2 — Source data Fig. 1 [file 44318_2025_519_MOESM2_ESM.zip › Figure 1 Source Data/Fig. 1A SD/HE P0 Control.tif]

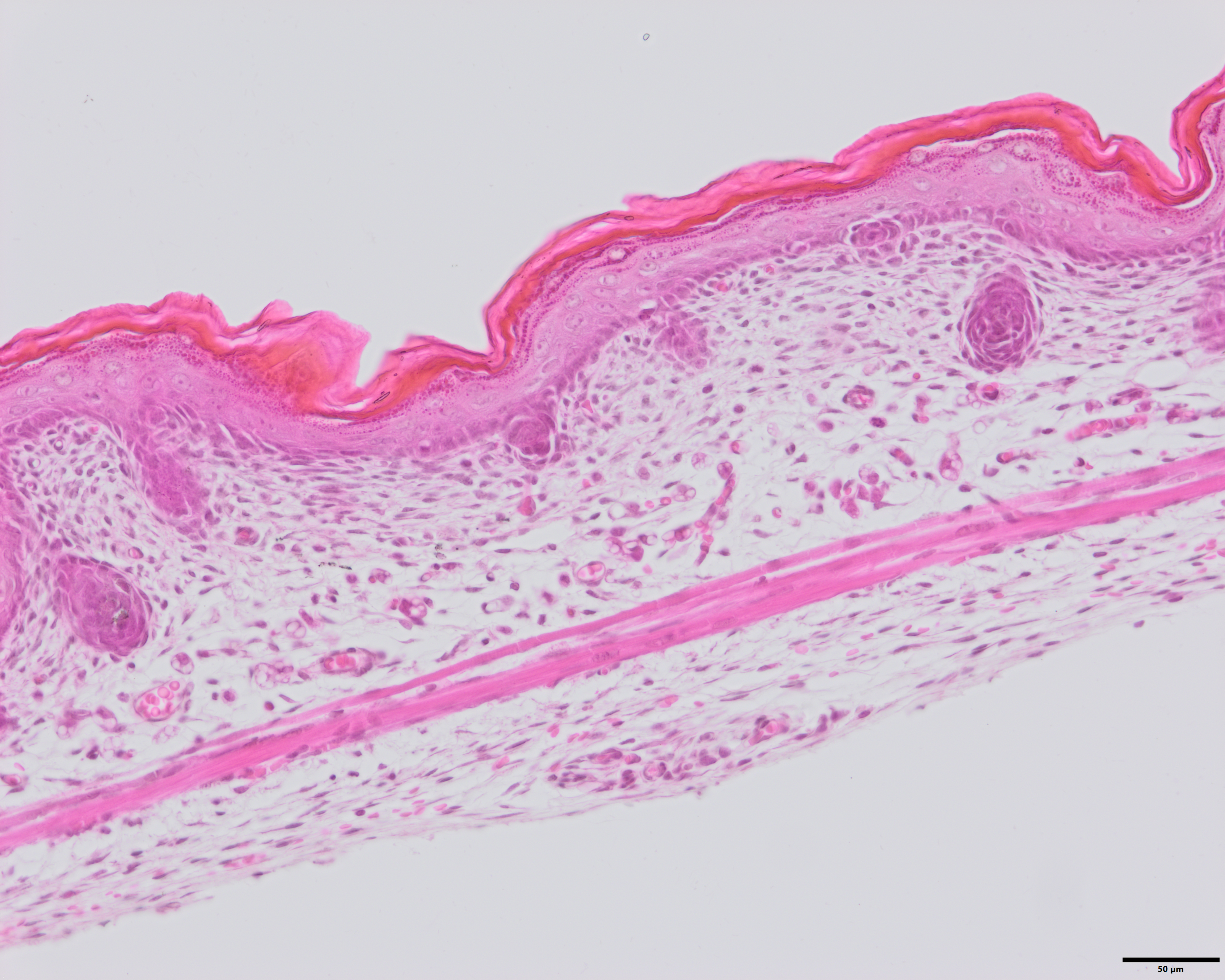

Supplement: Supplementary file 2 — Source data Fig. 1 [file 44318_2025_519_MOESM2_ESM.zip › Figure 1 Source Data/Fig. 1A SD/HE P0 Gli2EKO.tif]

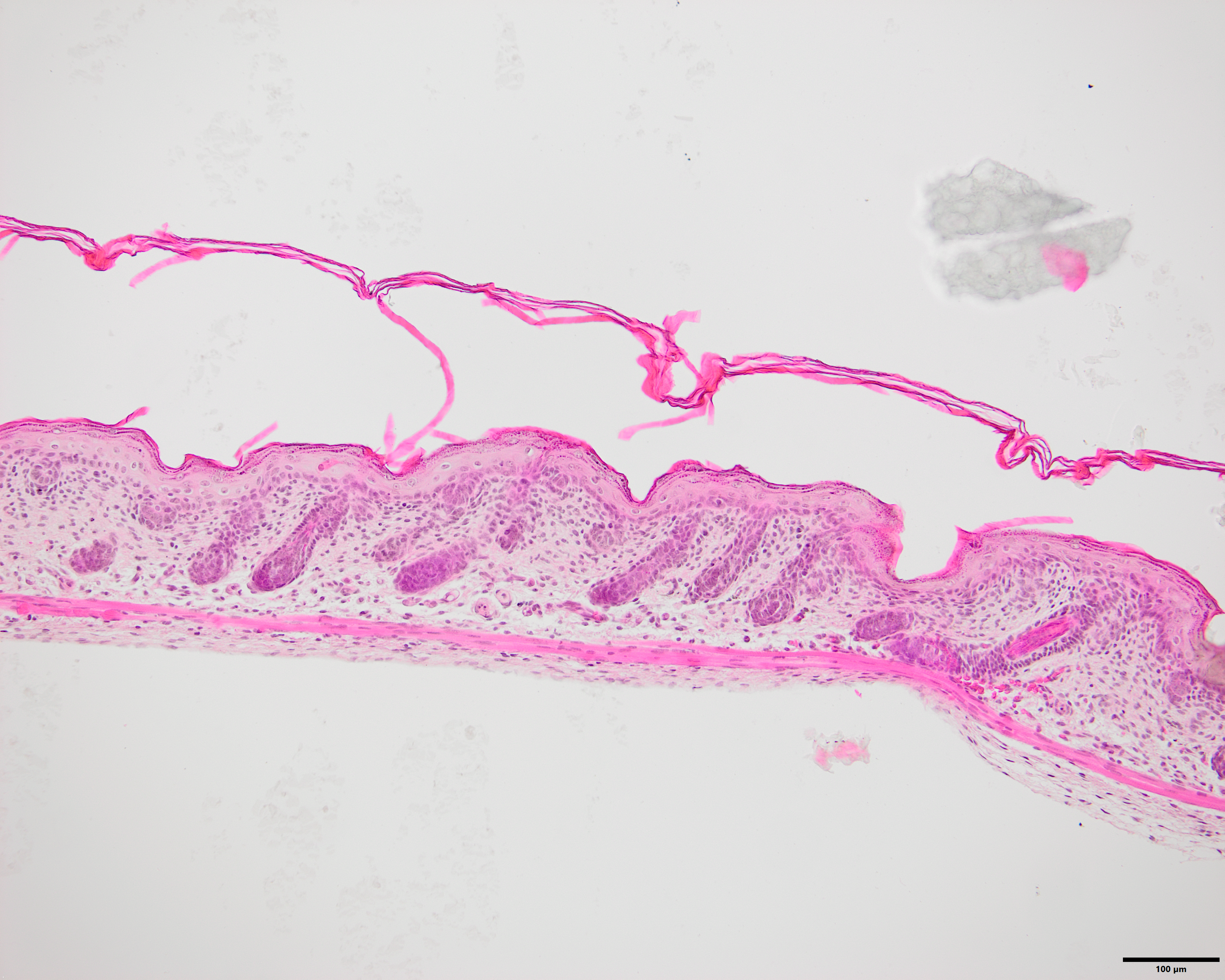

Supplement: Supplementary file 2 — Source data Fig. 1 [file 44318_2025_519_MOESM2_ESM.zip › Figure 1 Source Data/Fig. 1A SD/HE P0 Gli3EKO.tif]

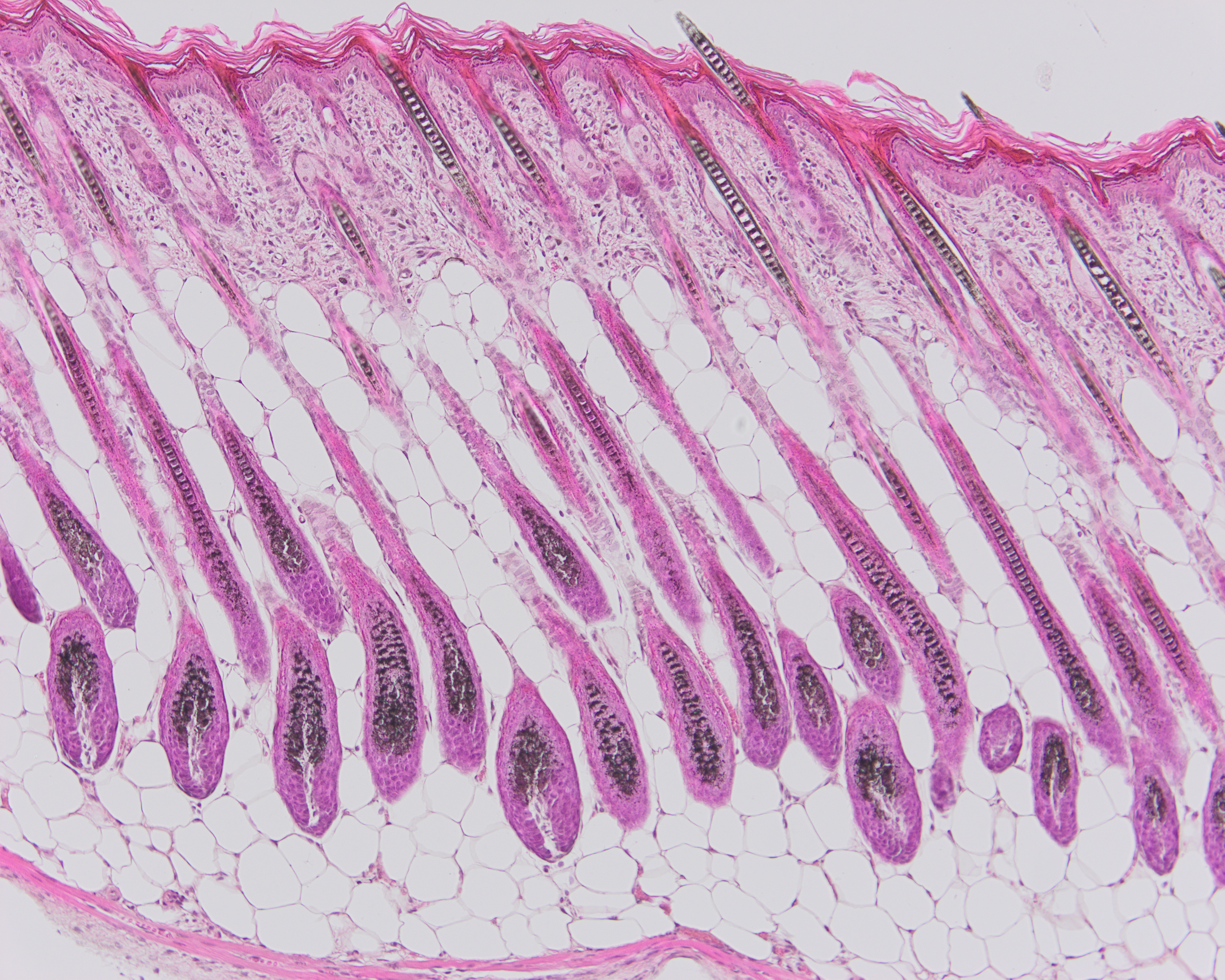

Supplement: Supplementary file 2 — Source data Fig. 1 [file 44318_2025_519_MOESM2_ESM.zip › Figure 1 Source Data/Fig. 1B SD/HE P6 Control.tif]

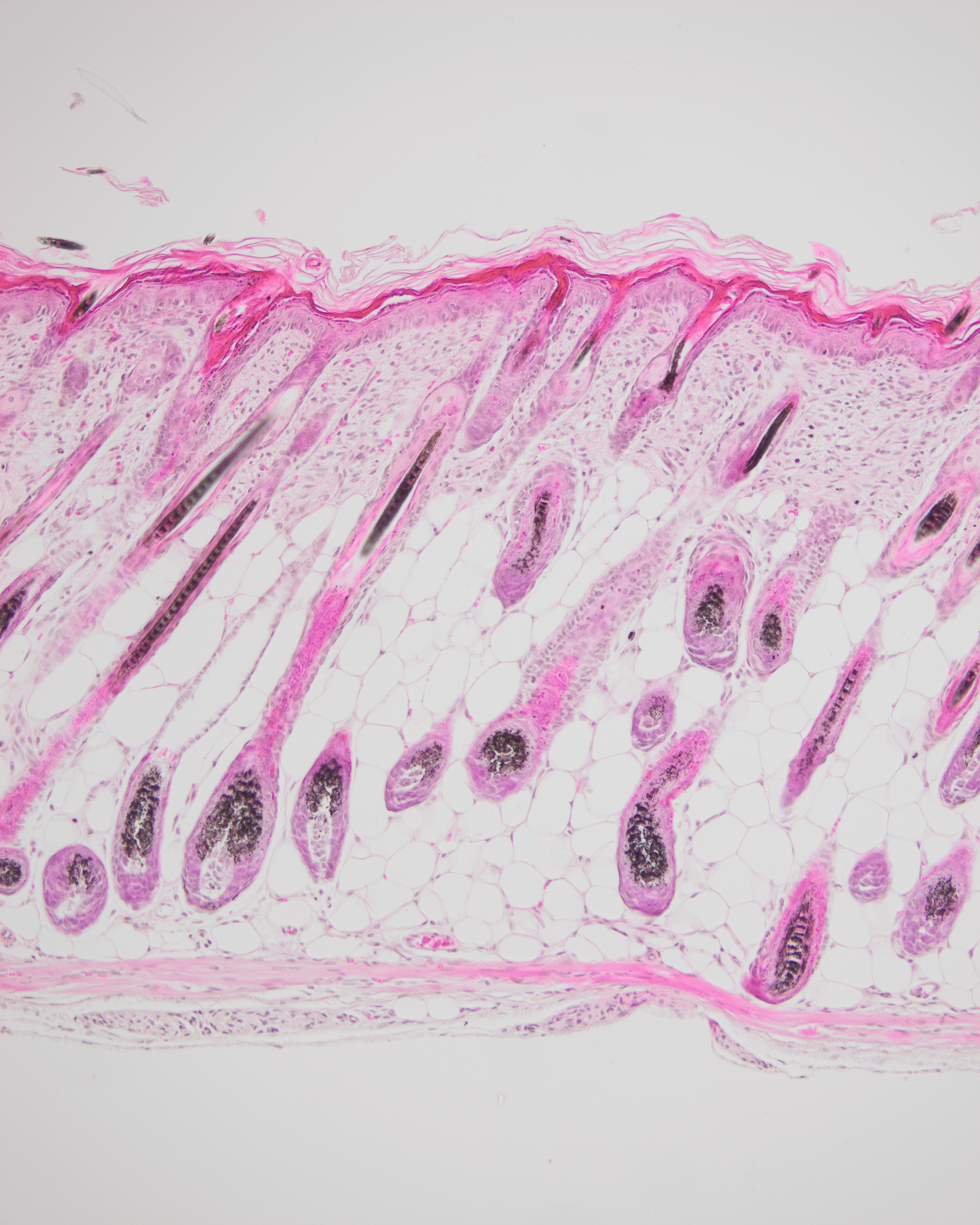

Supplement: Supplementary file 2 — Source data Fig. 1 [file 44318_2025_519_MOESM2_ESM.zip › Figure 1 Source Data/Fig. 1B SD/HE P6 Gli2EKO.tif]

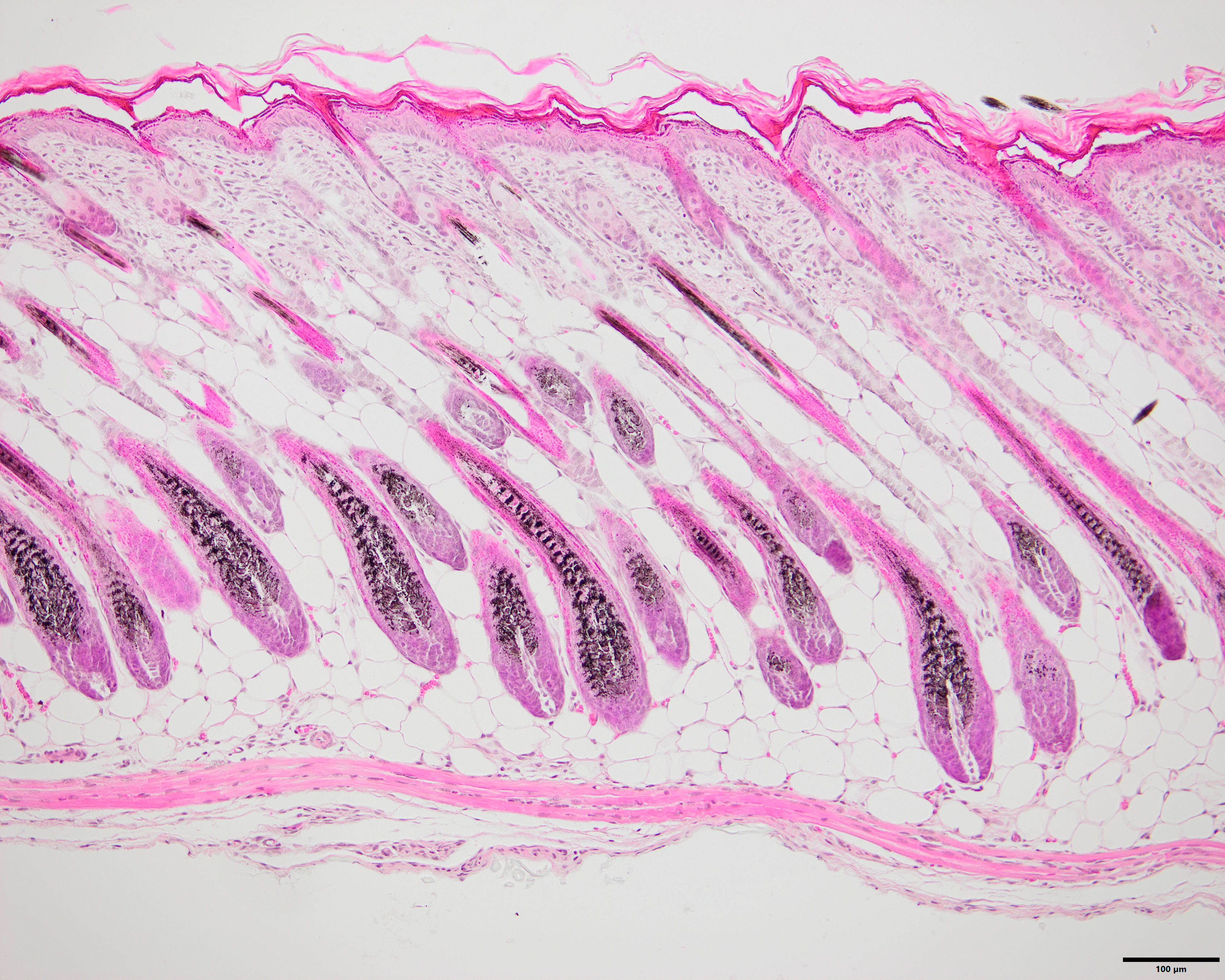

Supplement: Supplementary file 2 — Source data Fig. 1 [file 44318_2025_519_MOESM2_ESM.zip › Figure 1 Source Data/Fig. 1B SD/HE P6 Gli3EKO.tif]

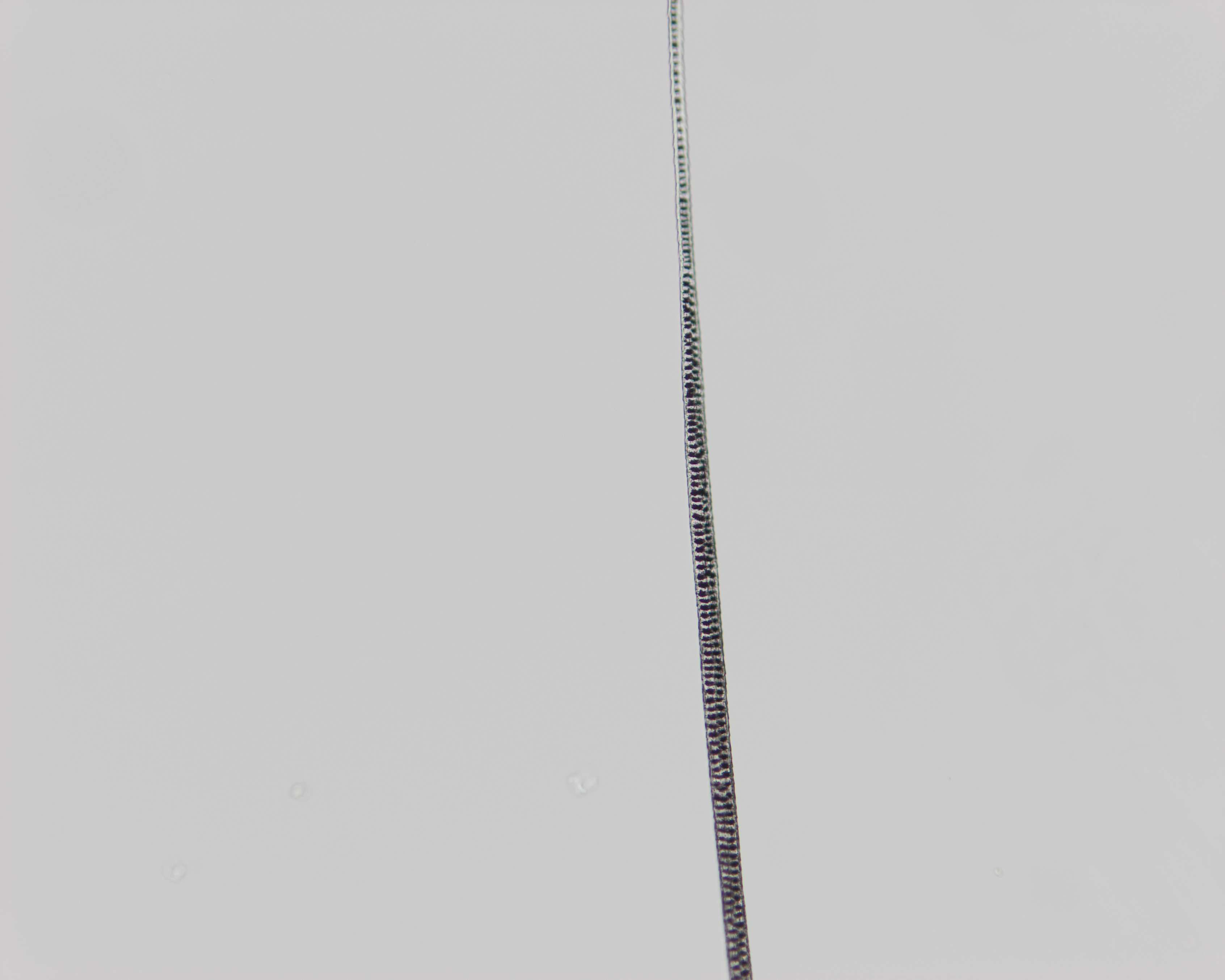

Supplement: Supplementary file 2 — Source data Fig. 1 [file 44318_2025_519_MOESM2_ESM.zip › Figure 1 Source Data/Fig. 1D SD/Auchene.png]

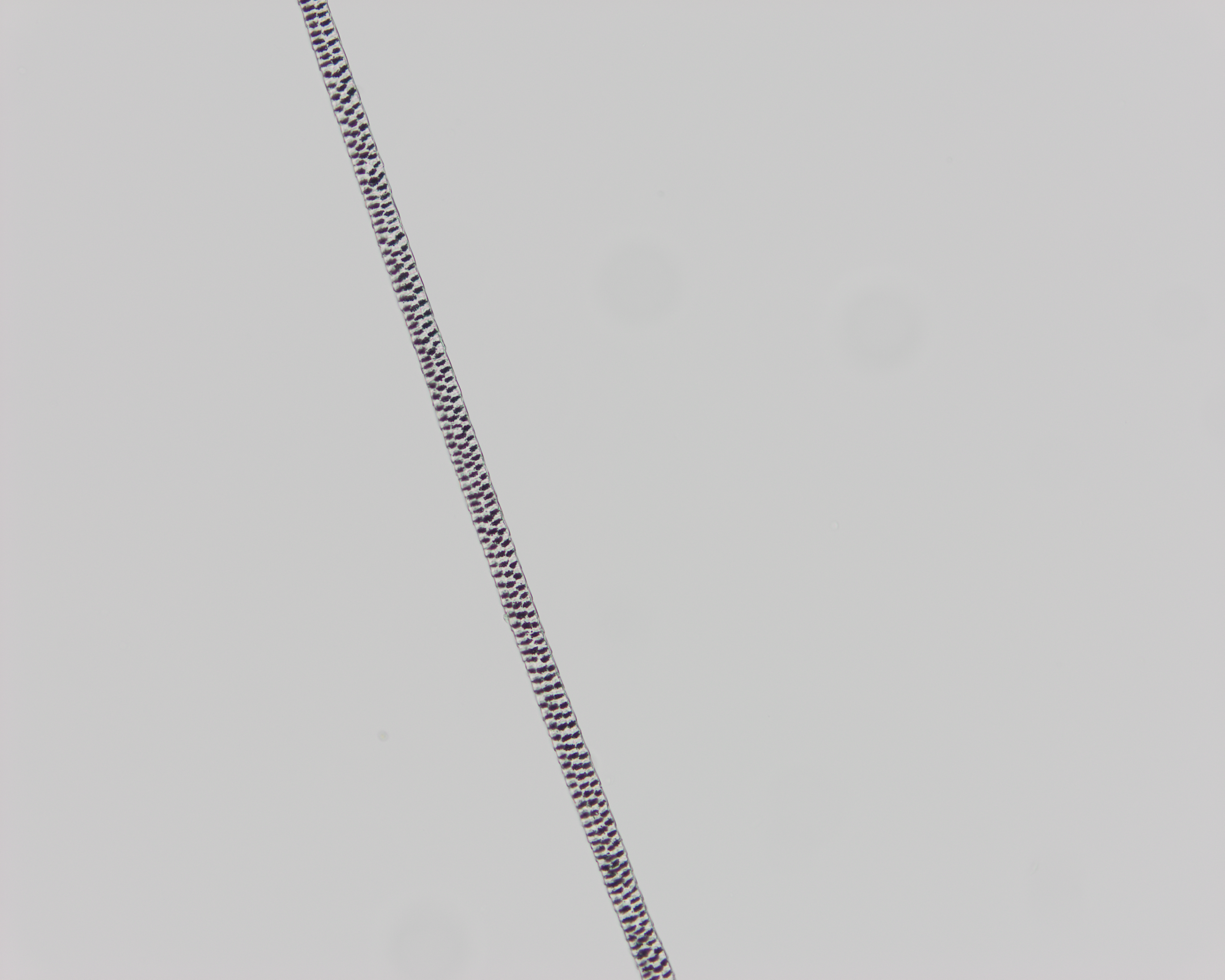

Supplement: Supplementary file 2 — Source data Fig. 1 [file 44318_2025_519_MOESM2_ESM.zip › Figure 1 Source Data/Fig. 1D SD/Awl.png]

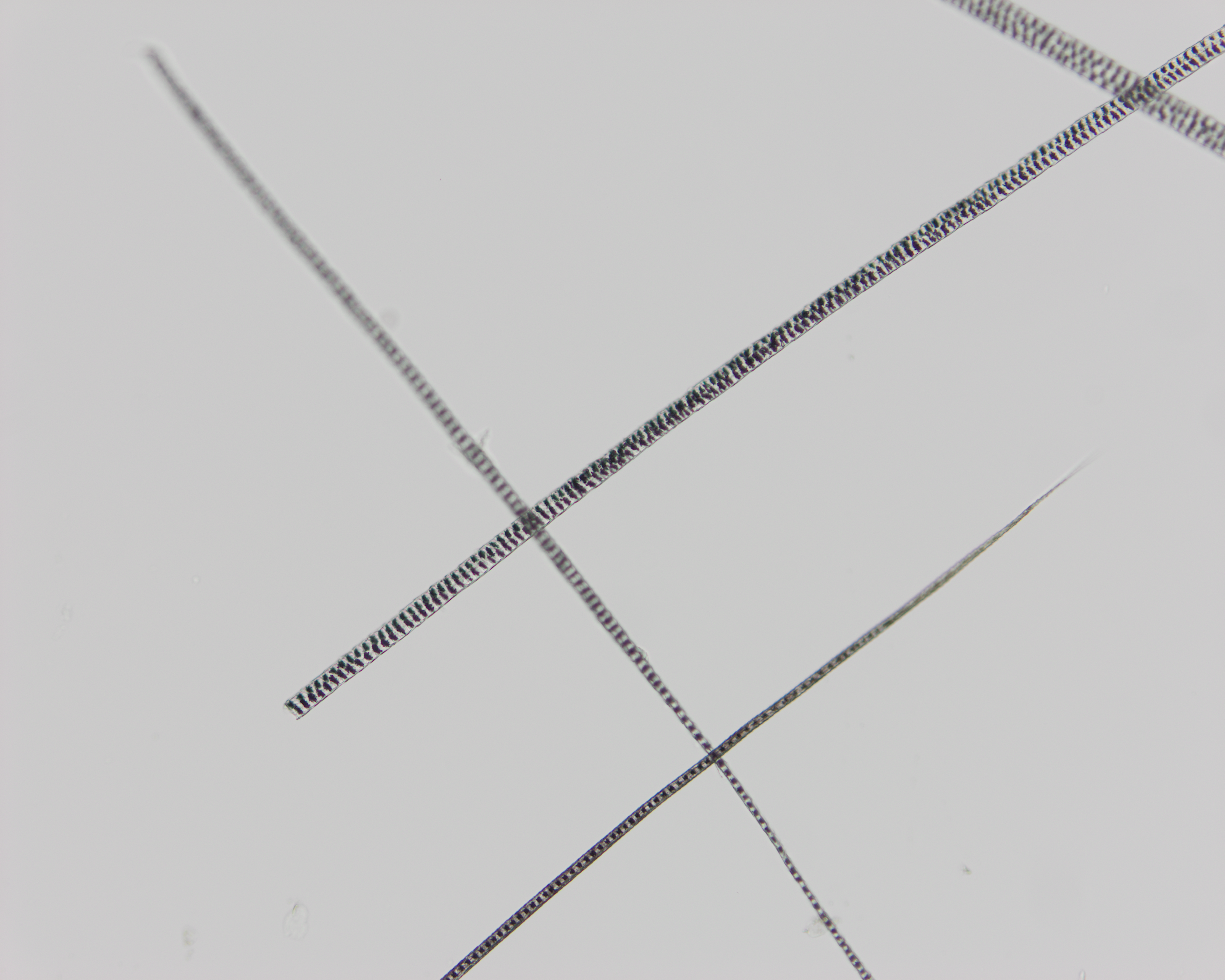

Supplement: Supplementary file 2 — Source data Fig. 1 [file 44318_2025_519_MOESM2_ESM.zip › Figure 1 Source Data/Fig. 1D SD/Guard.png]

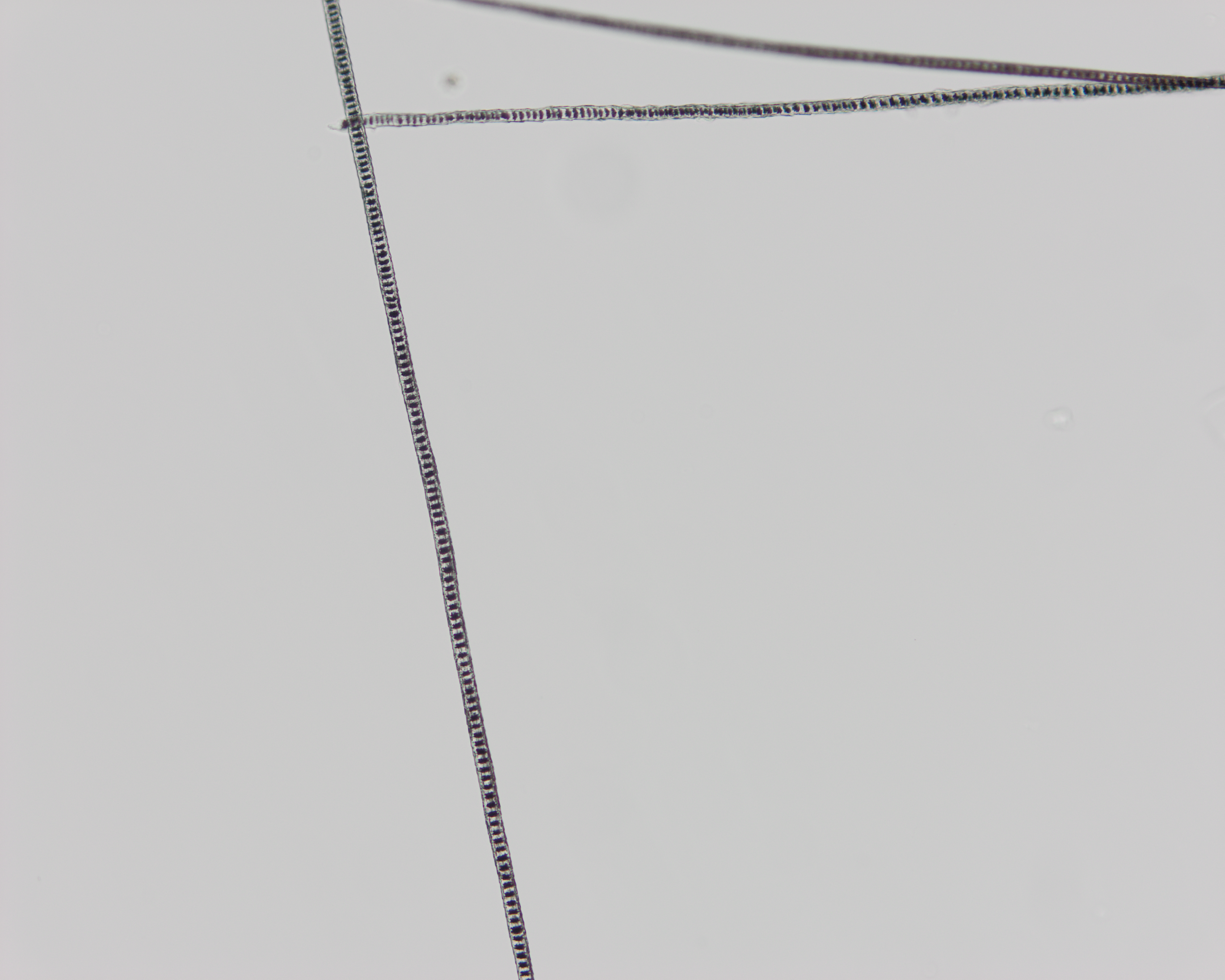

Supplement: Supplementary file 2 — Source data Fig. 1 [file 44318_2025_519_MOESM2_ESM.zip › Figure 1 Source Data/Fig. 1D SD/Zigzag.png]

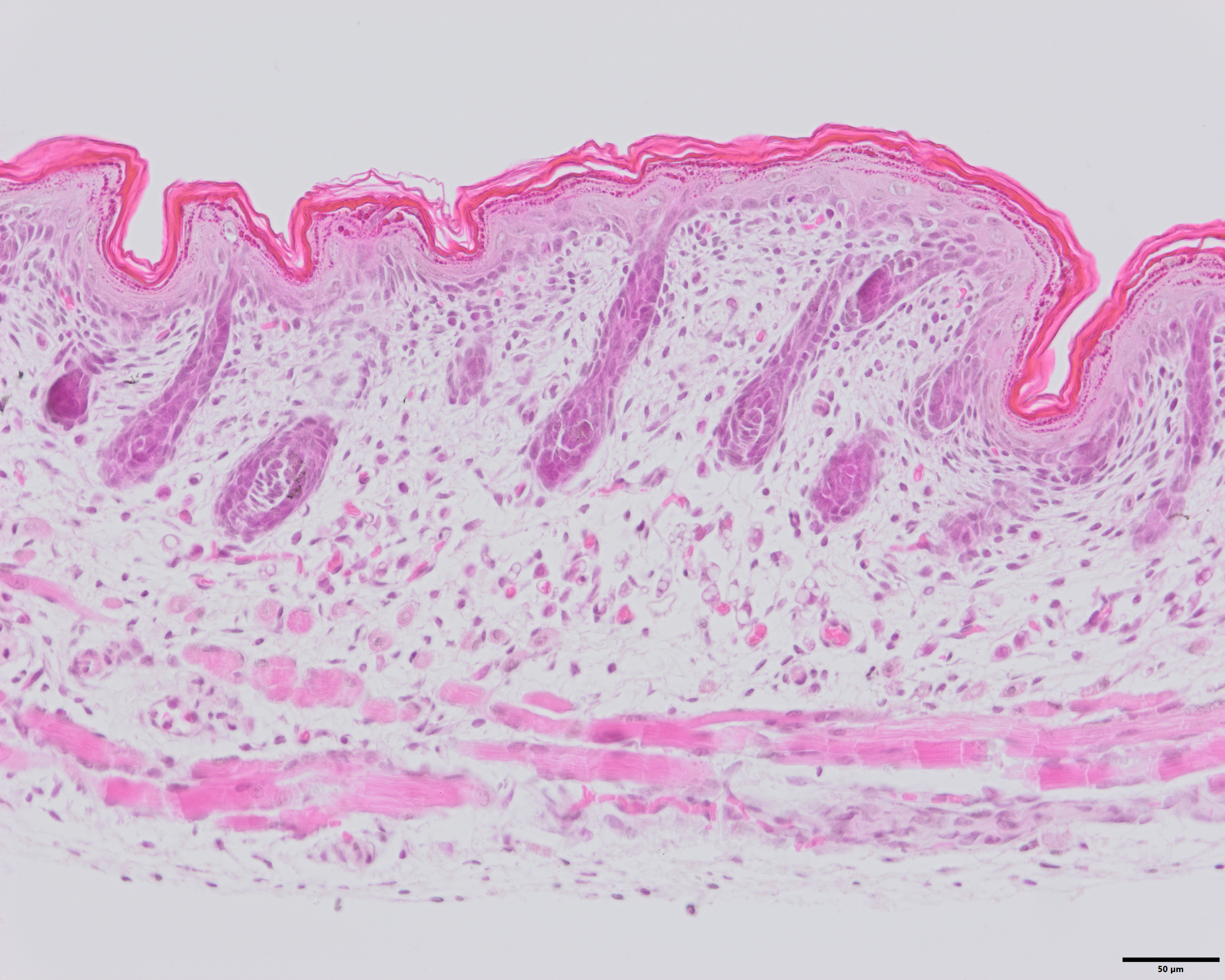

Supplement: Supplementary file 2 — Source data Fig. 1 [file 44318_2025_519_MOESM2_ESM.zip › Figure 1 Source Data/Fig. 1E SD/HE P0 Control.tif]

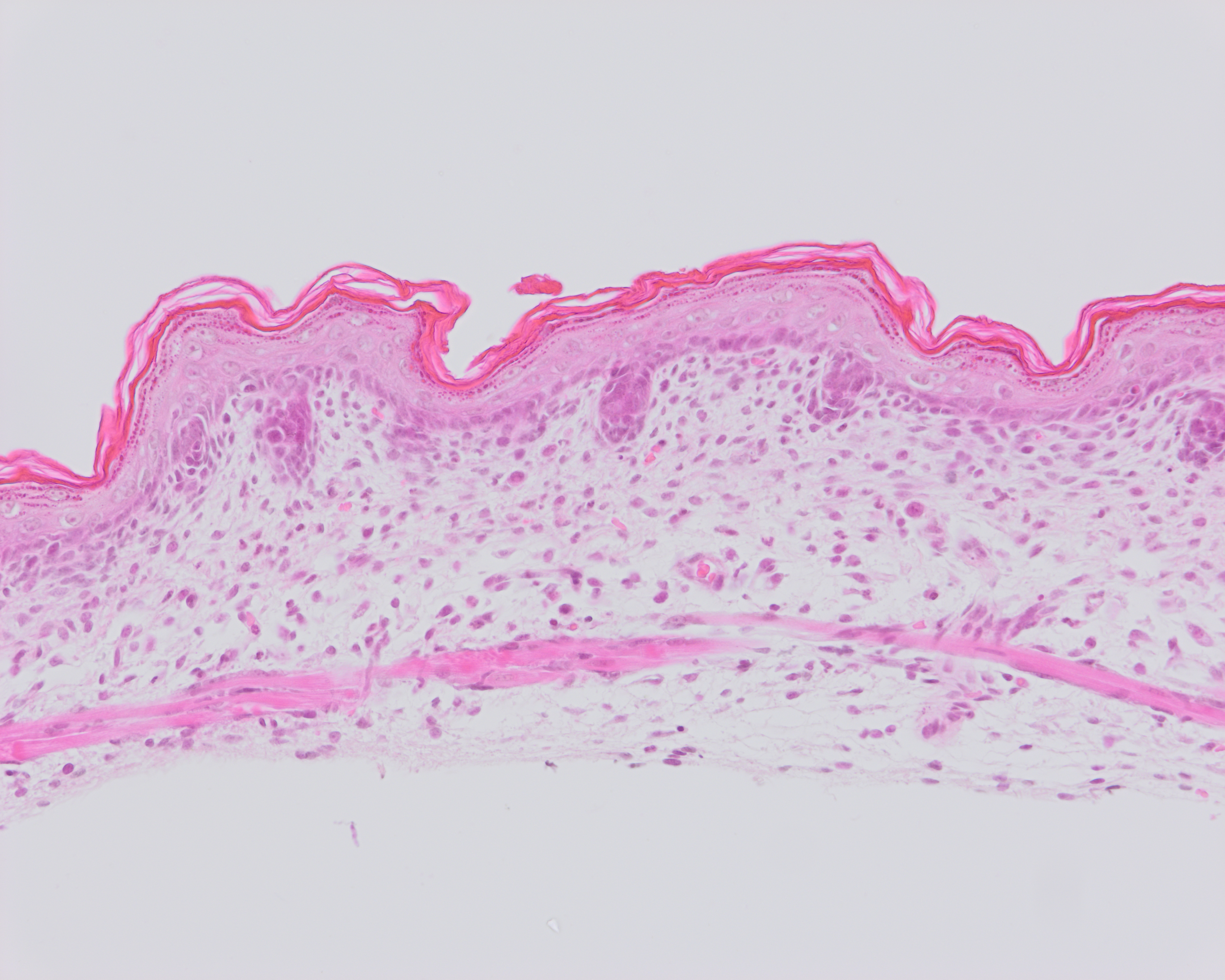

Supplement: Supplementary file 2 — Source data Fig. 1 [file 44318_2025_519_MOESM2_ESM.zip › Figure 1 Source Data/Fig. 1E SD/HE P0 Gli2-3EKO.tif]

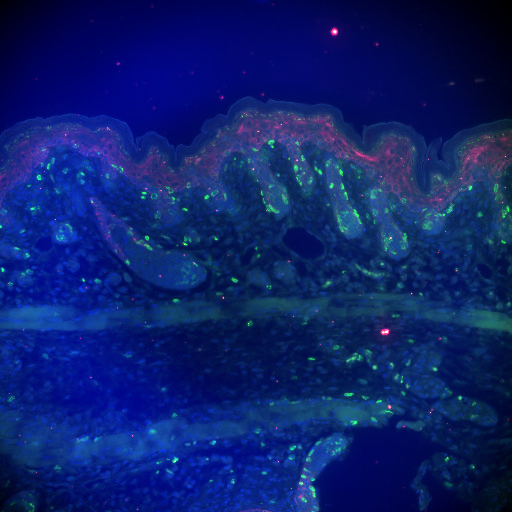

Supplement: Supplementary file 2 — Source data Fig. 1 [file 44318_2025_519_MOESM2_ESM.zip › Figure 1 Source Data/Fig. 1F SD/P0 Control.tif]

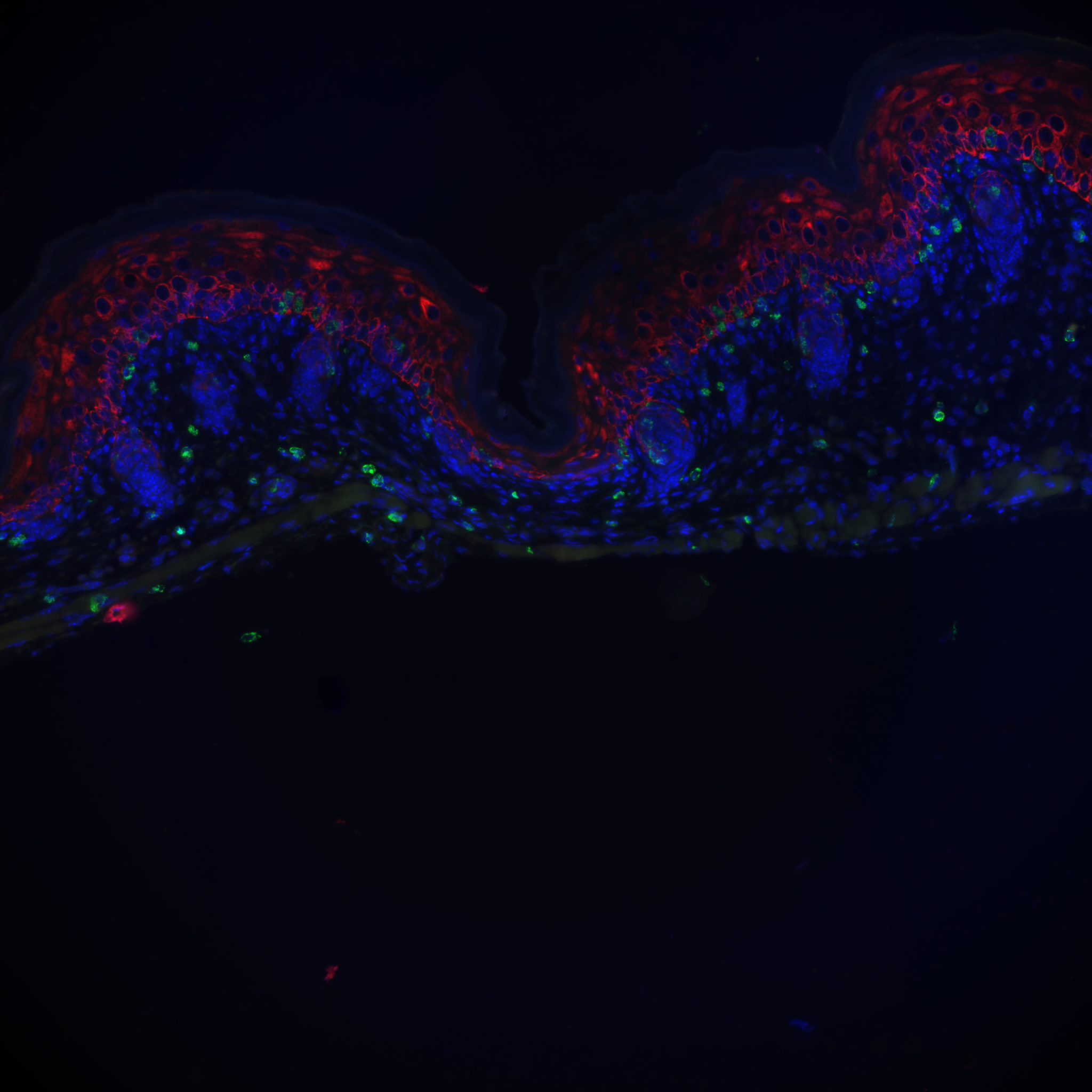

Supplement: Supplementary file 2 — Source data Fig. 1 [file 44318_2025_519_MOESM2_ESM.zip › Figure 1 Source Data/Fig. 1F SD/P0 Gli2-3EKO.tif]

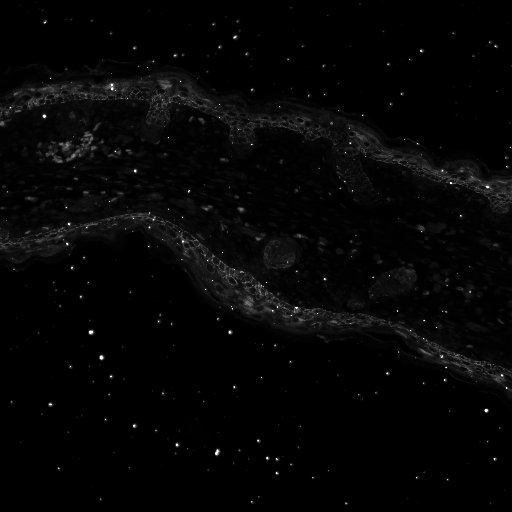

Supplement: Supplementary file 2 — Source data Fig. 1 [file 44318_2025_519_MOESM2_ESM.zip › Figure 1 Source Data/Fig. 1F SD/P0 Gli2EKO.tif]

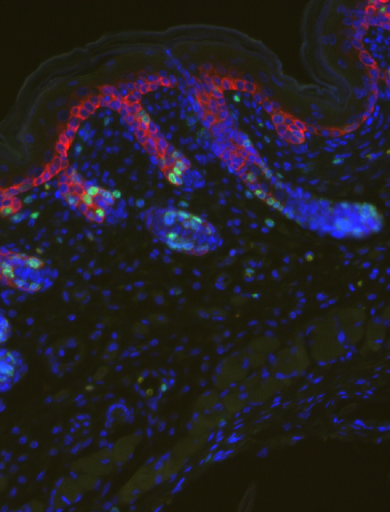

Supplement: Supplementary file 2 — Source data Fig. 1 [file 44318_2025_519_MOESM2_ESM.zip › Figure 1 Source Data/Fig. 1F SD/P0 Gli3EKO.tif]

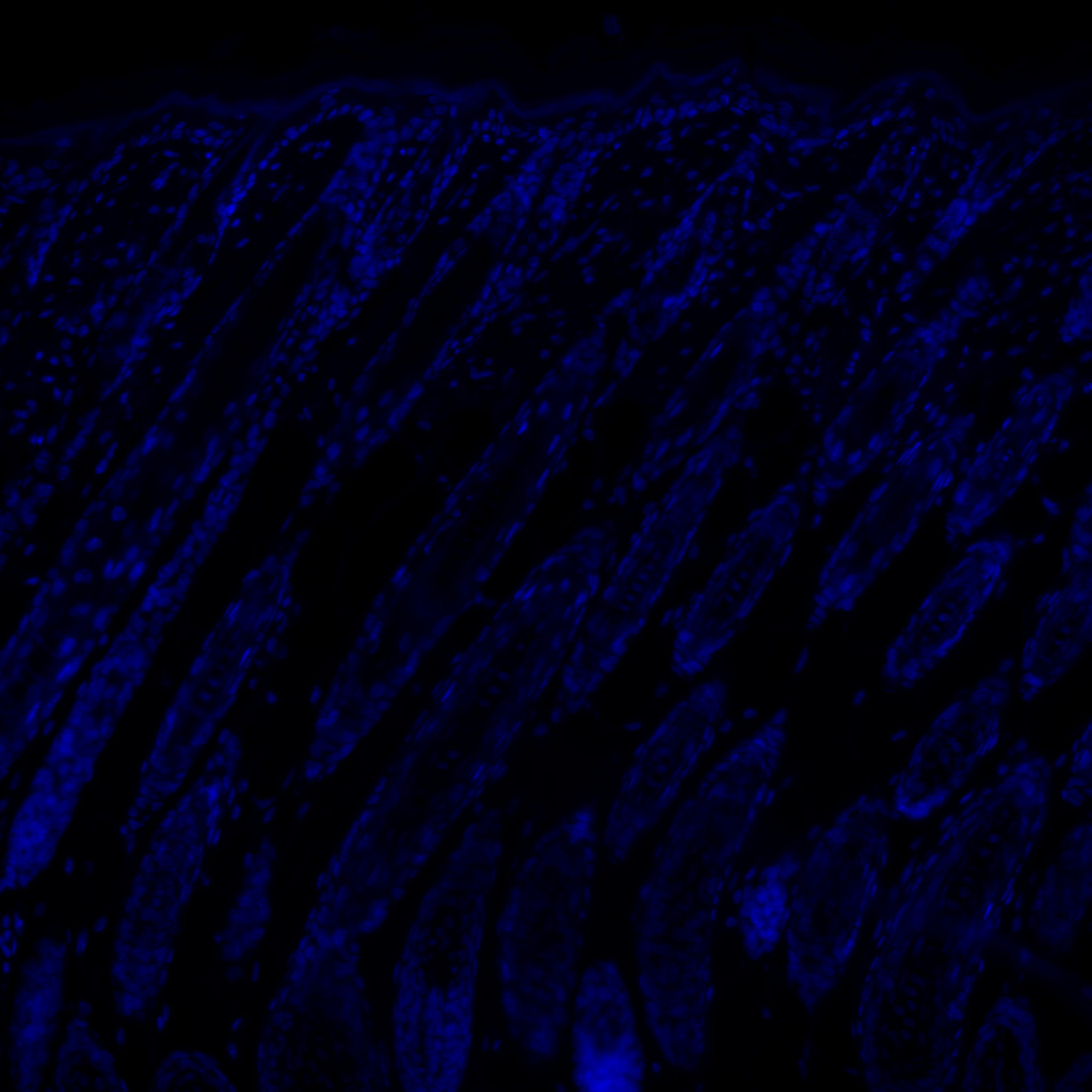

Supplement: Supplementary file 3 — Source data Fig. 2 [file 44318_2025_519_MOESM3_ESM.zip › Figure 2 Source Data/Fig. 2A SD/K14/P6 Control DAPI.tif]

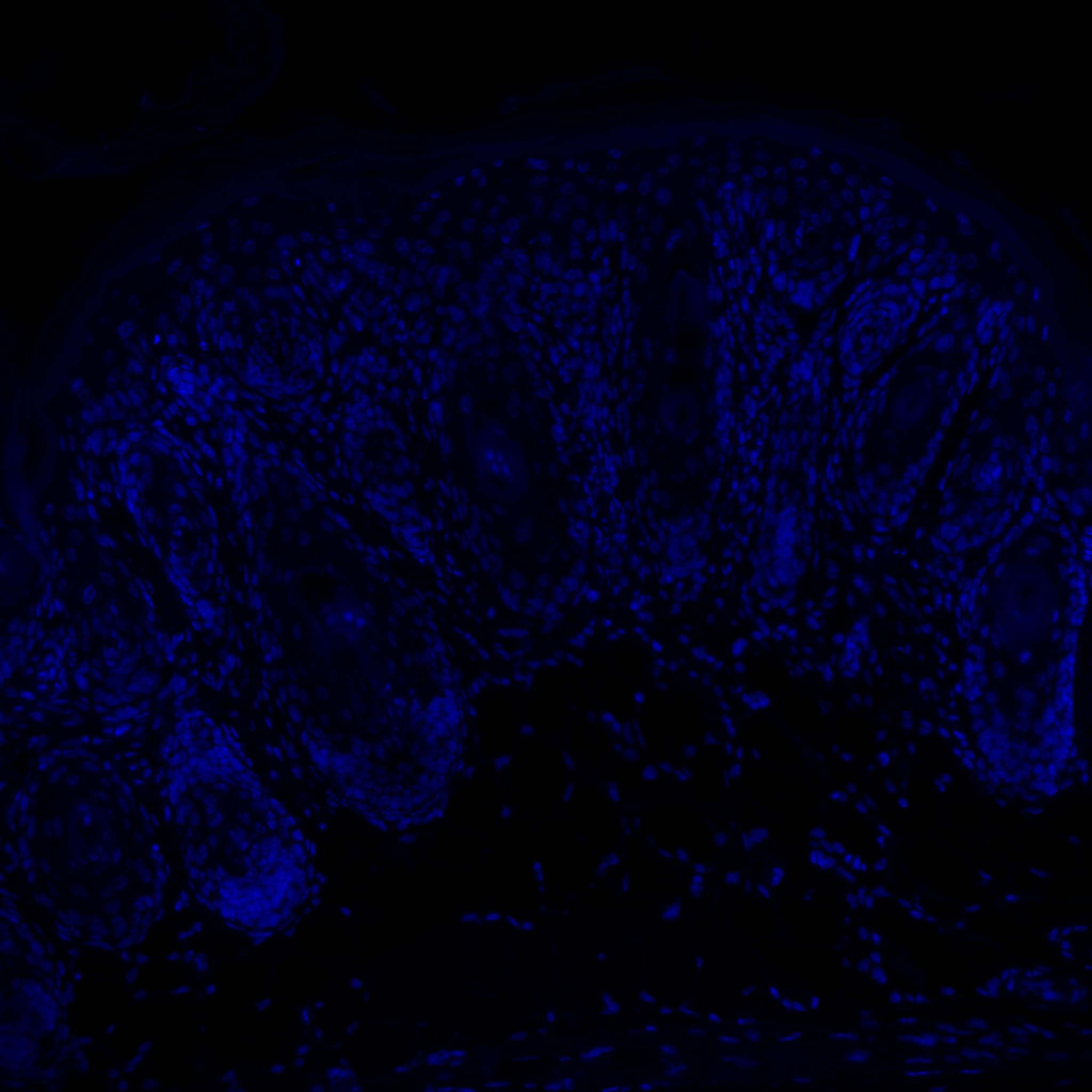

Supplement: Supplementary file 3 — Source data Fig. 2 [file 44318_2025_519_MOESM3_ESM.zip › Figure 2 Source Data/Fig. 2A SD/K14/P6 Gli2-3EKO DAPI.tif]

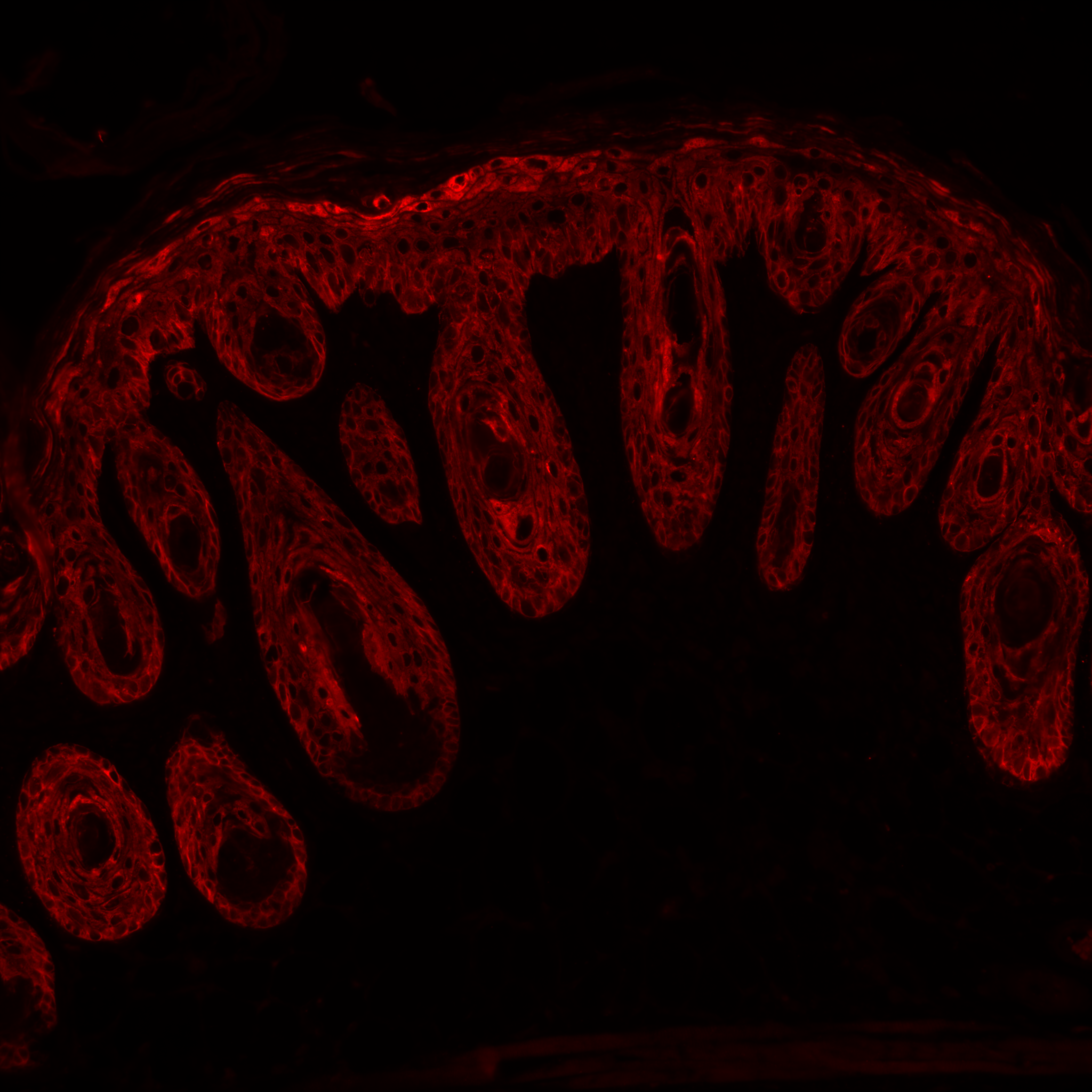

Supplement: Supplementary file 3 — Source data Fig. 2 [file 44318_2025_519_MOESM3_ESM.zip › Figure 2 Source Data/Fig. 2A SD/K14/P6 Gli2-3EKO K14.tif]

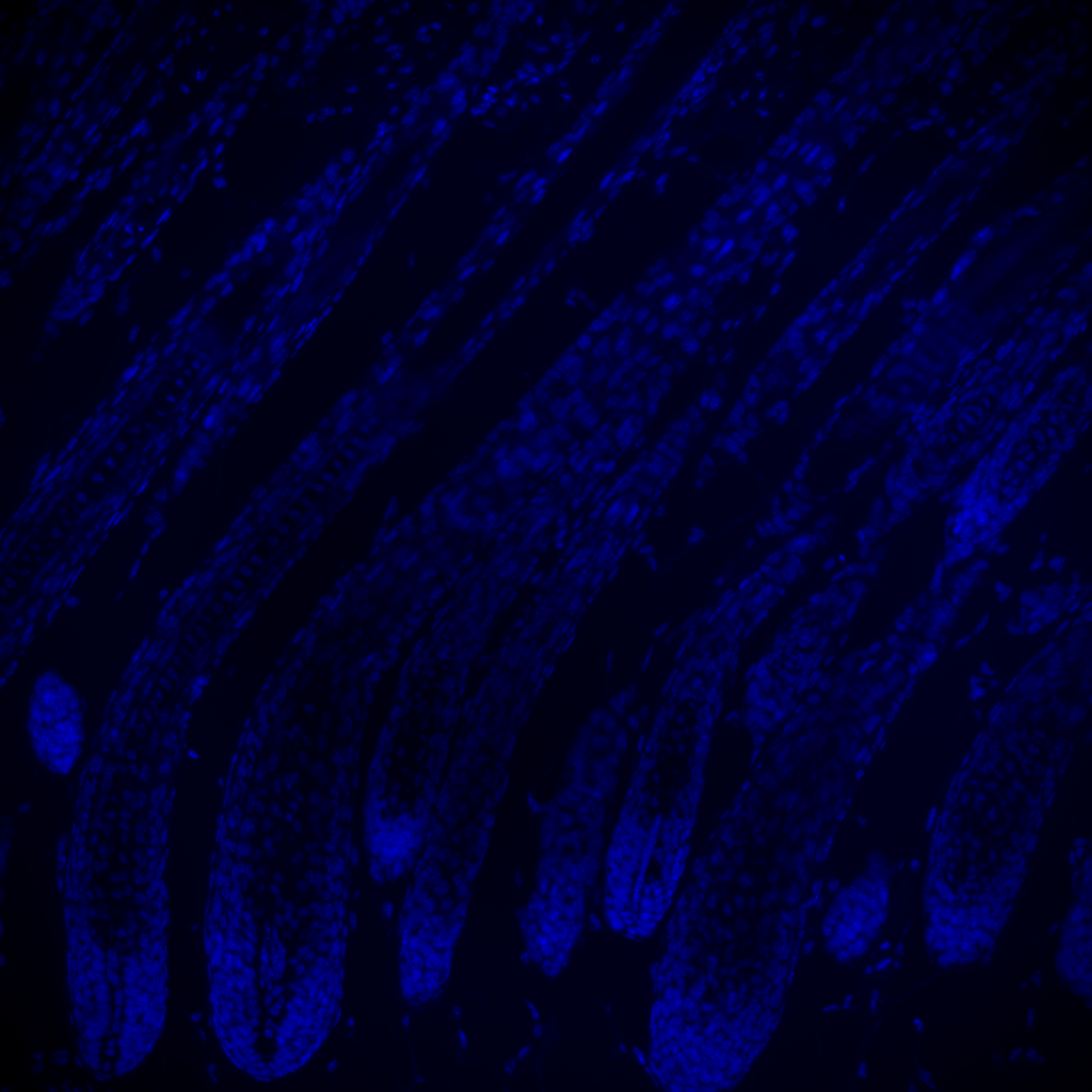

Supplement: Supplementary file 3 — Source data Fig. 2 [file 44318_2025_519_MOESM3_ESM.zip › Figure 2 Source Data/Fig. 2A SD/K71/P6 Control DAPI.tif]

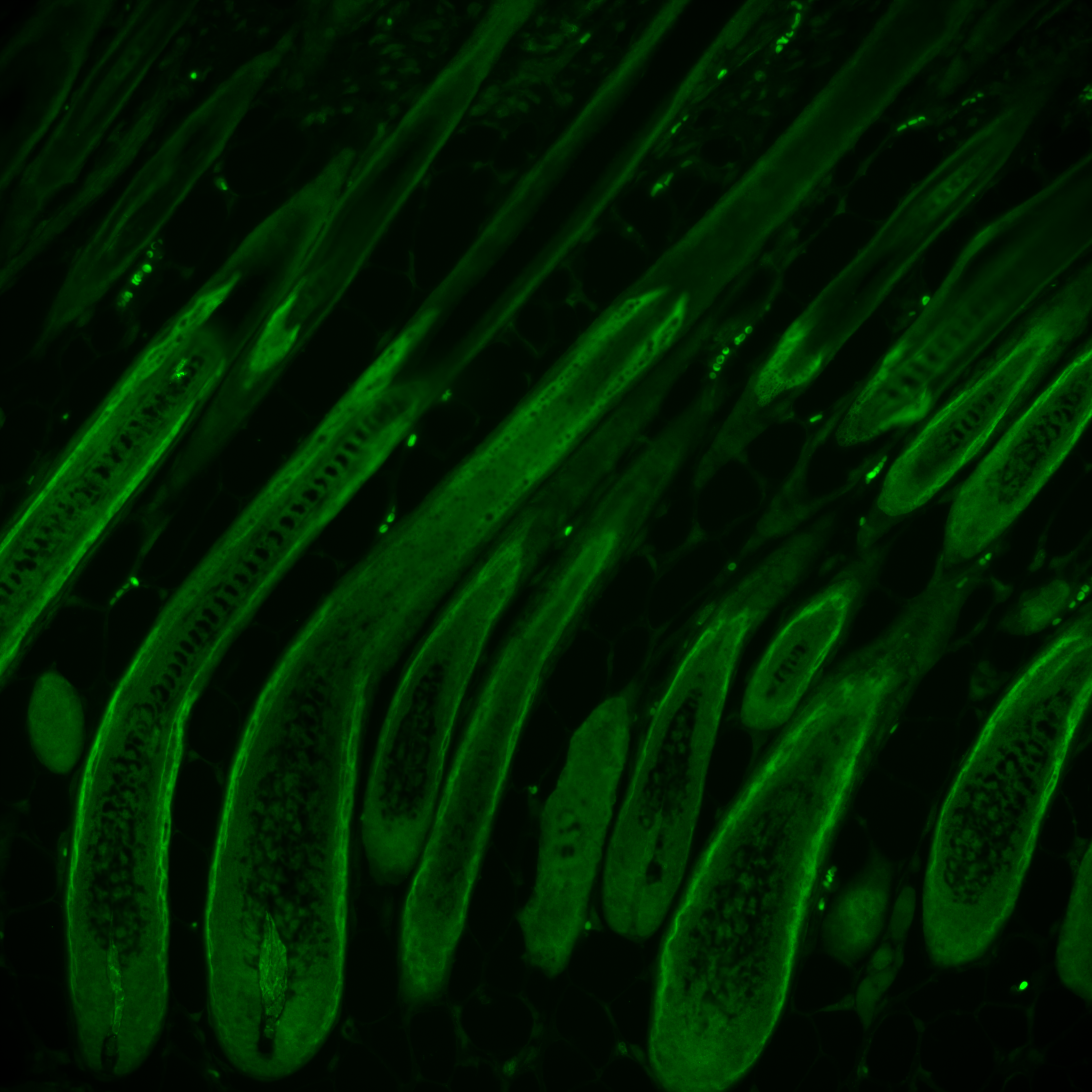

Supplement: Supplementary file 3 — Source data Fig. 2 [file 44318_2025_519_MOESM3_ESM.zip › Figure 2 Source Data/Fig. 2A SD/K71/P6 Control K71.tif]

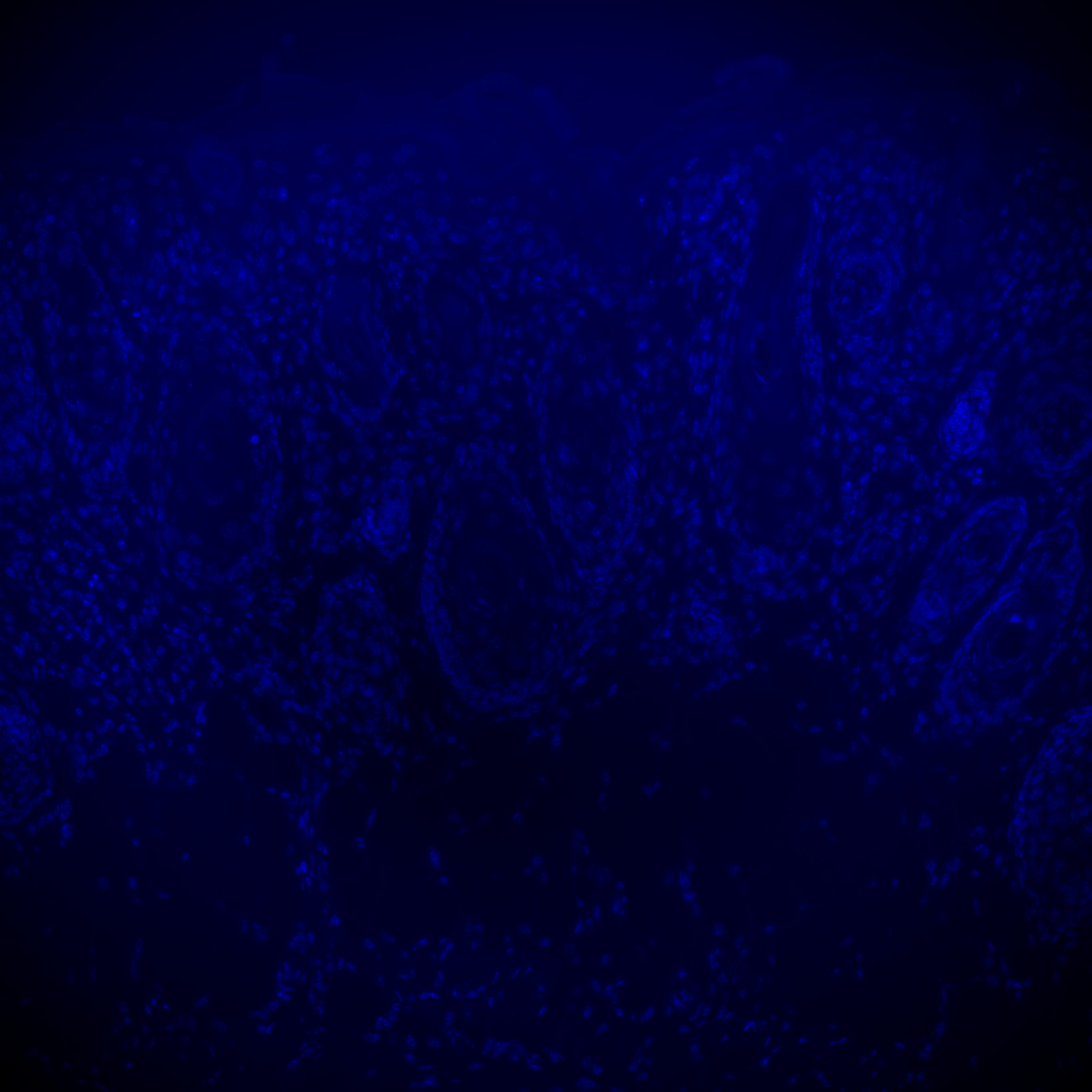

Supplement: Supplementary file 3 — Source data Fig. 2 [file 44318_2025_519_MOESM3_ESM.zip › Figure 2 Source Data/Fig. 2A SD/K71/P6 Gli2-3EKO DAPI.tif]

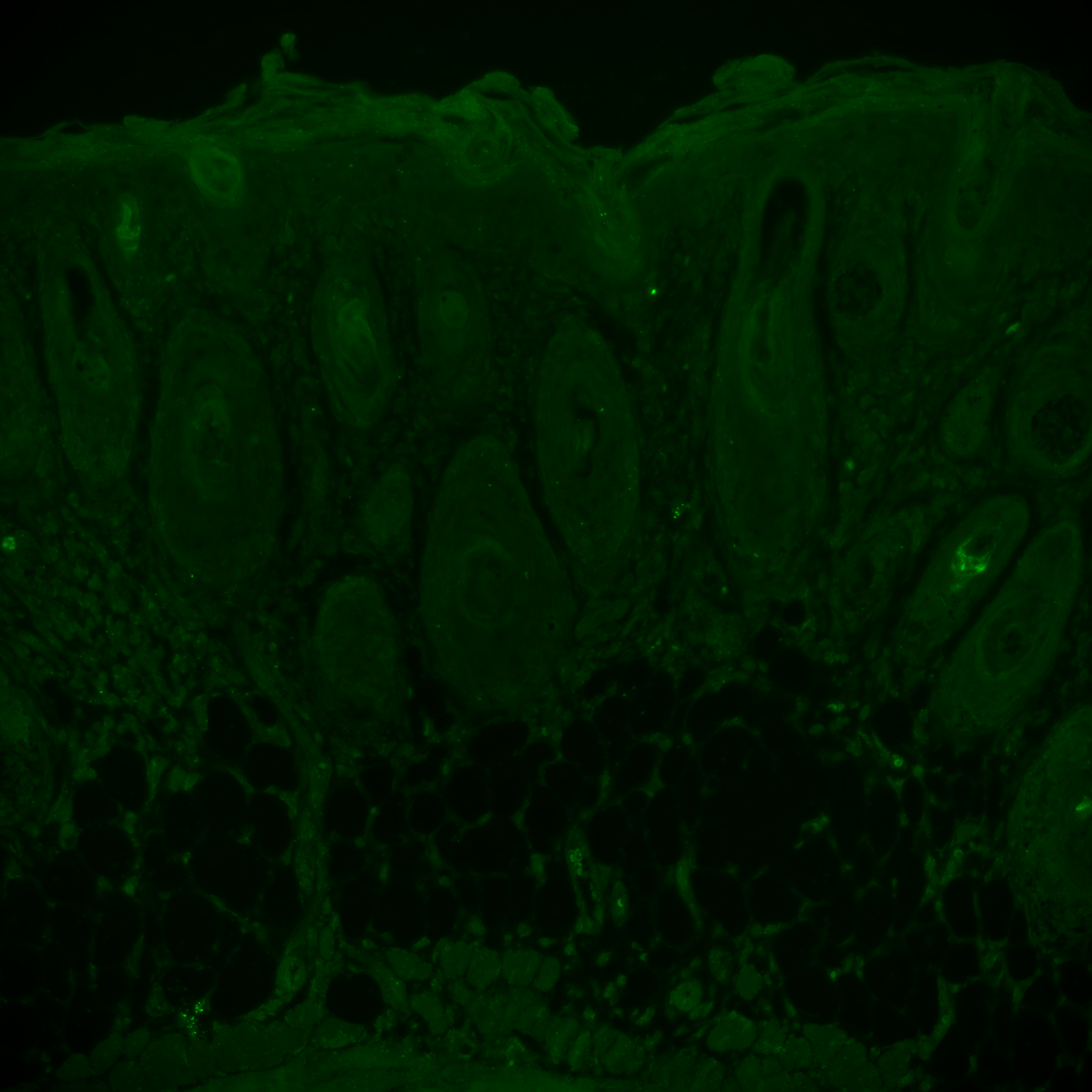

Supplement: Supplementary file 3 — Source data Fig. 2 [file 44318_2025_519_MOESM3_ESM.zip › Figure 2 Source Data/Fig. 2A SD/K71/P6 Gli2-3EKO K71.tif]

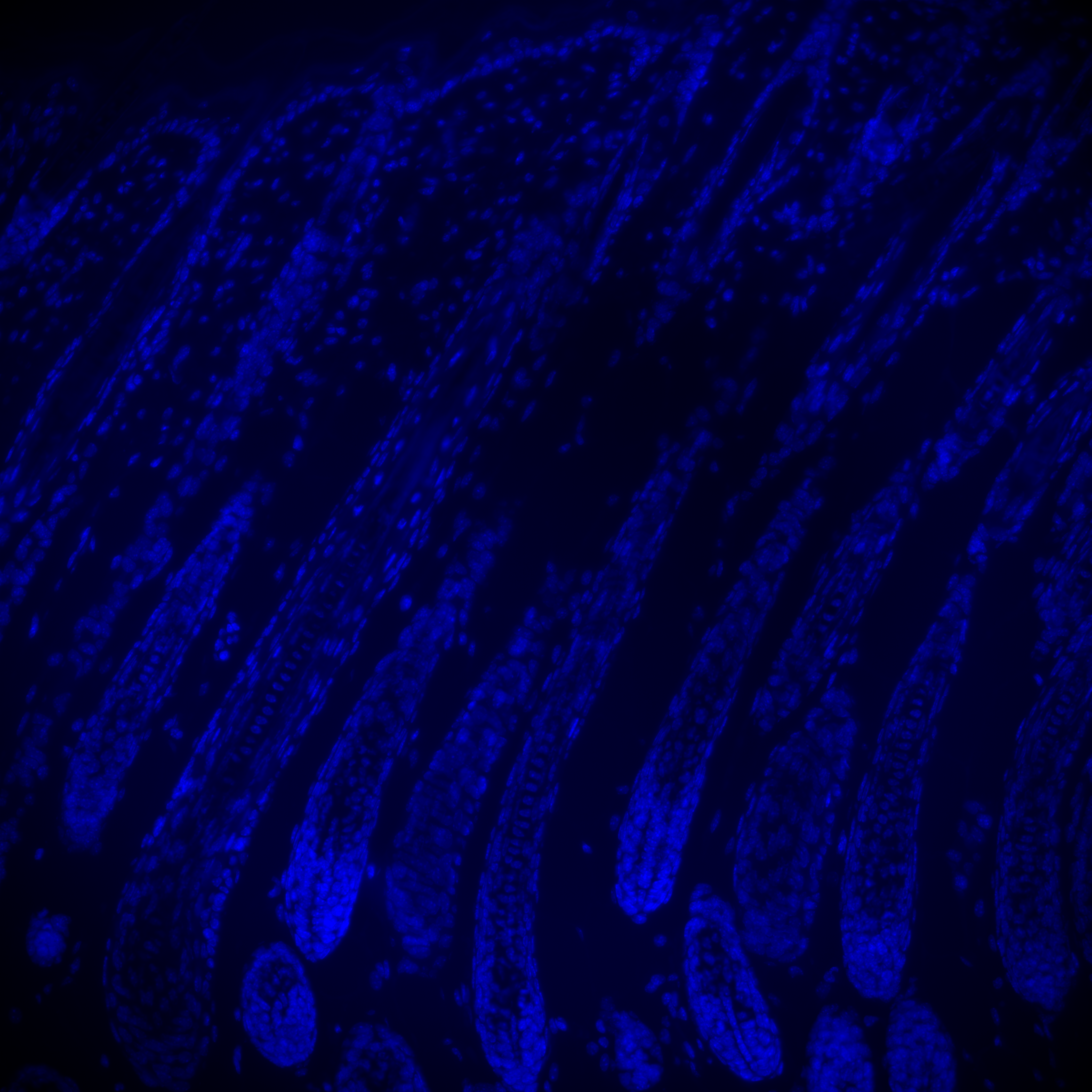

Supplement: Supplementary file 3 — Source data Fig. 2 [file 44318_2025_519_MOESM3_ESM.zip › Figure 2 Source Data/Fig. 2A SD/K75/P6 Control DAPI.tif]

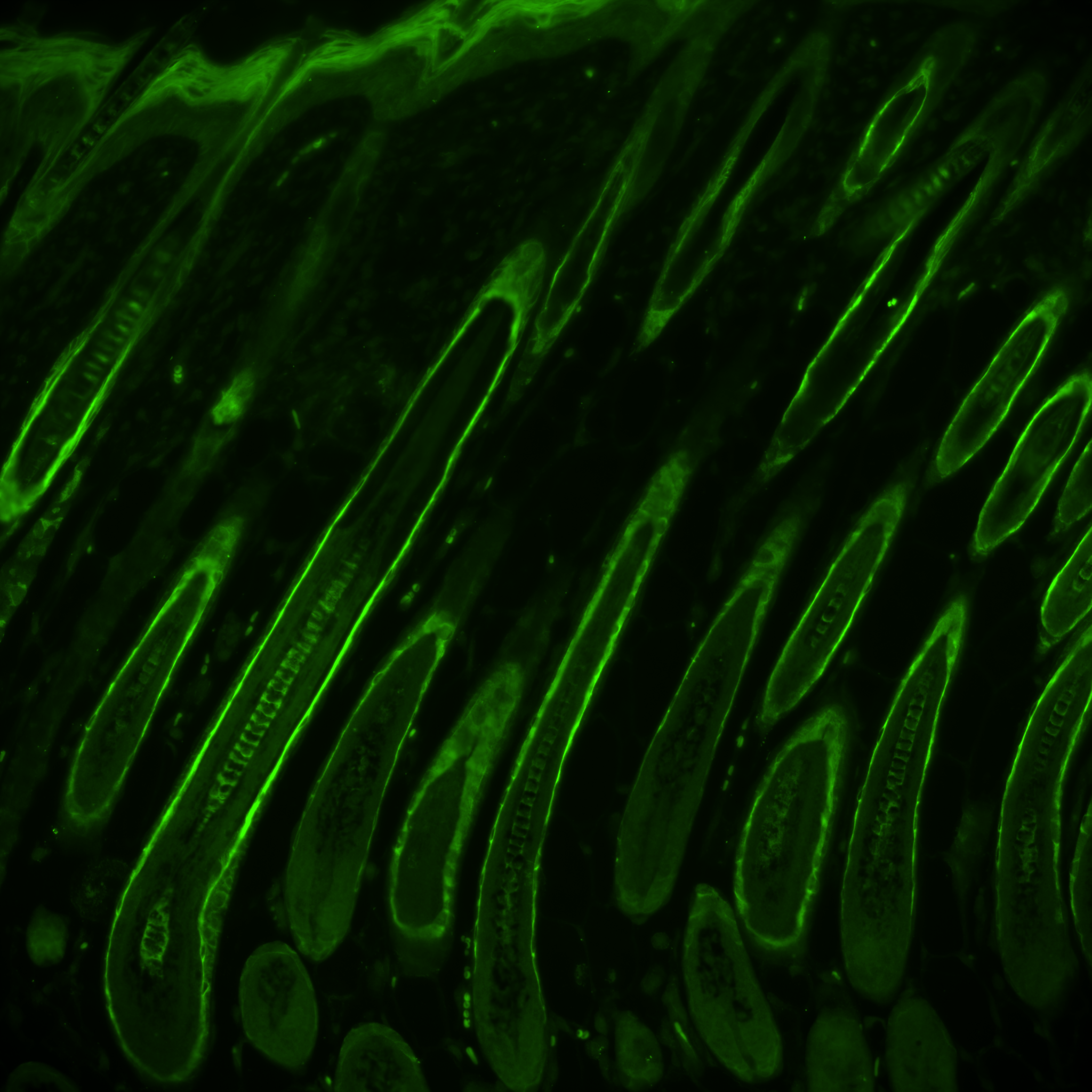

Supplement: Supplementary file 3 — Source data Fig. 2 [file 44318_2025_519_MOESM3_ESM.zip › Figure 2 Source Data/Fig. 2A SD/K75/P6 Control K75.tif]

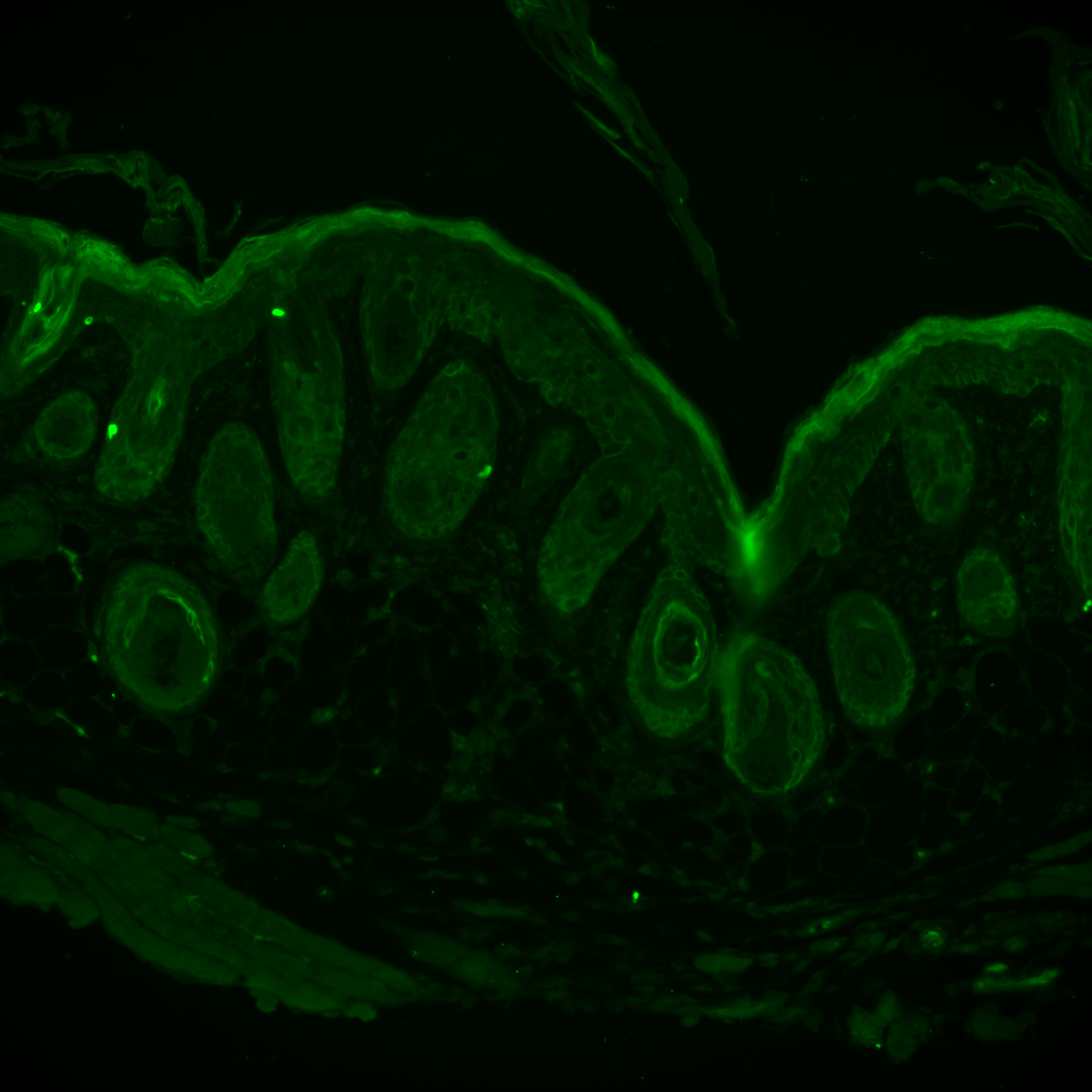

Supplement: Supplementary file 3 — Source data Fig. 2 [file 44318_2025_519_MOESM3_ESM.zip › Figure 2 Source Data/Fig. 2A SD/K75/P6 Gli2-3EKO K75.tif]

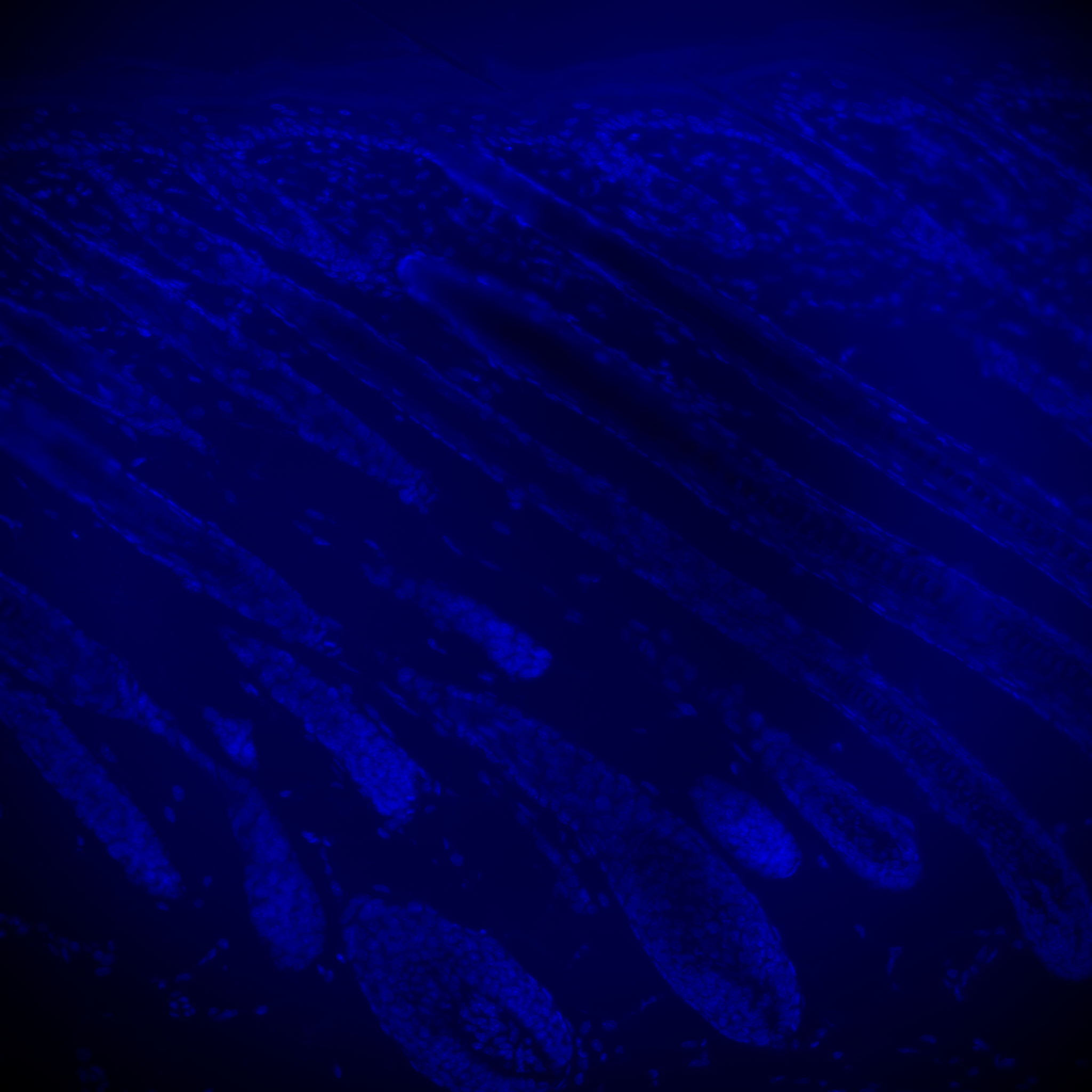

Supplement: Supplementary file 3 — Source data Fig. 2 [file 44318_2025_519_MOESM3_ESM.zip › Figure 2 Source Data/Fig. 2A SD/K86/P6 Control DAPI.tif]

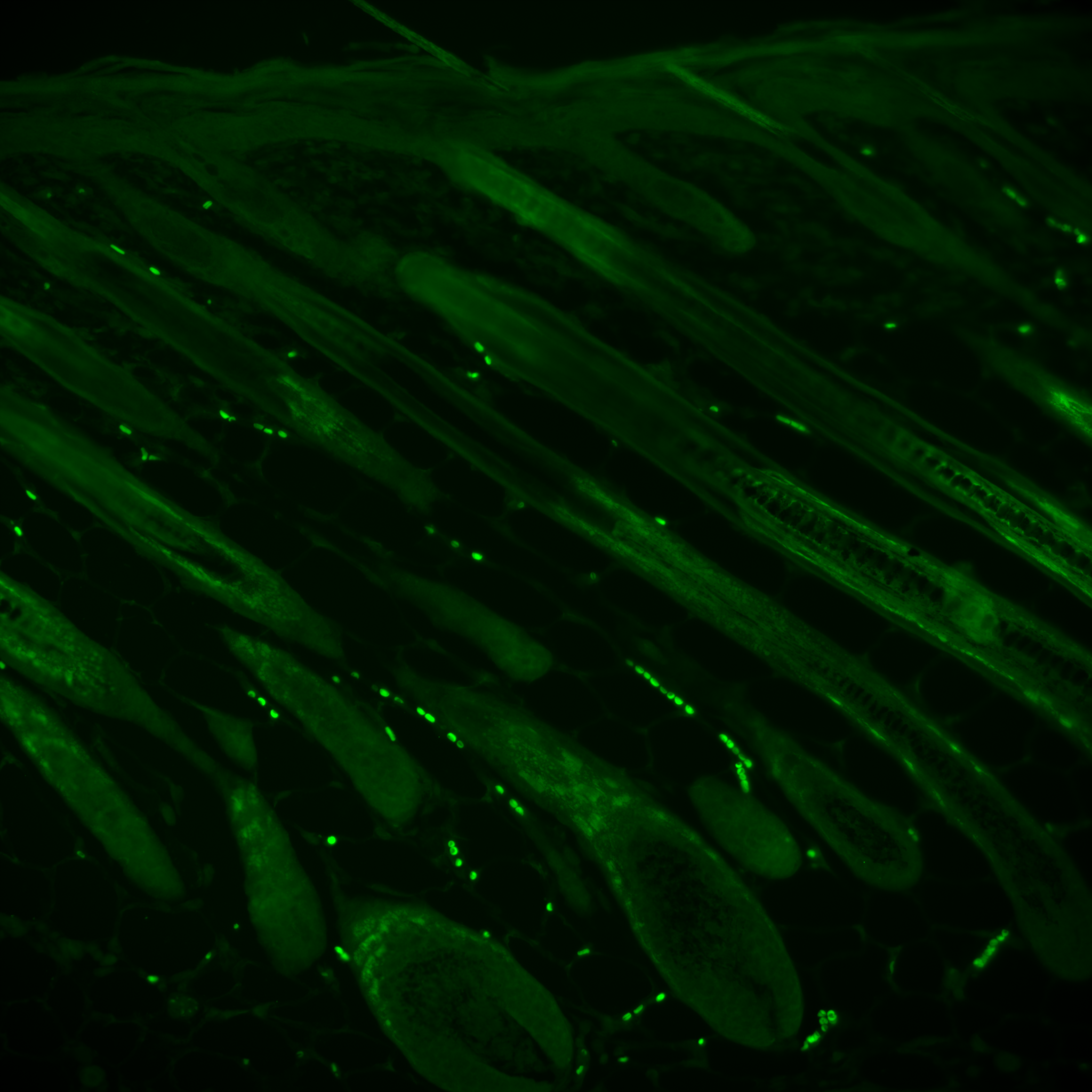

Supplement: Supplementary file 3 — Source data Fig. 2 [file 44318_2025_519_MOESM3_ESM.zip › Figure 2 Source Data/Fig. 2A SD/K86/P6 Control K86.tif]

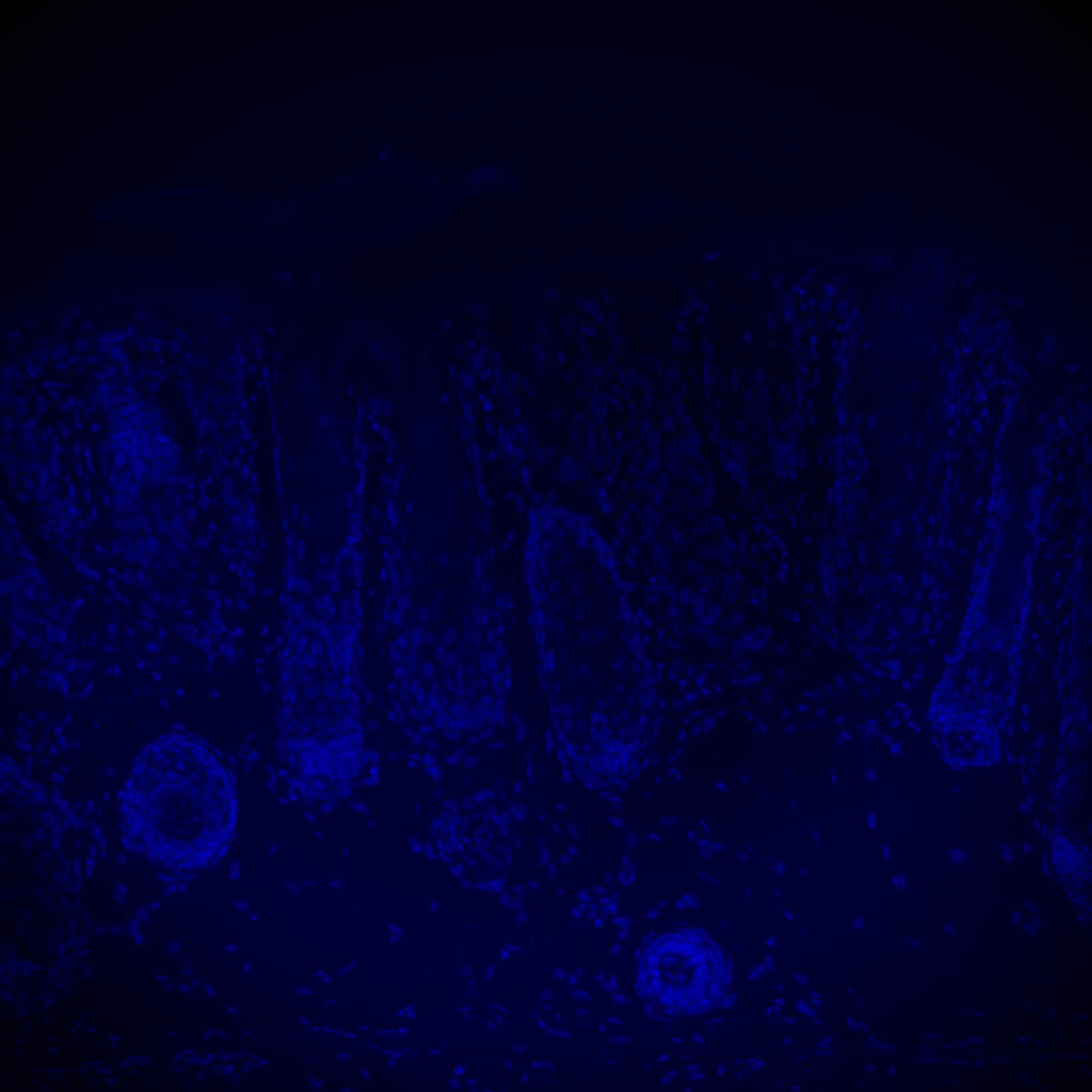

Supplement: Supplementary file 3 — Source data Fig. 2 [file 44318_2025_519_MOESM3_ESM.zip › Figure 2 Source Data/Fig. 2A SD/K86/P6 Gli2-3EKO DAPI.tif]

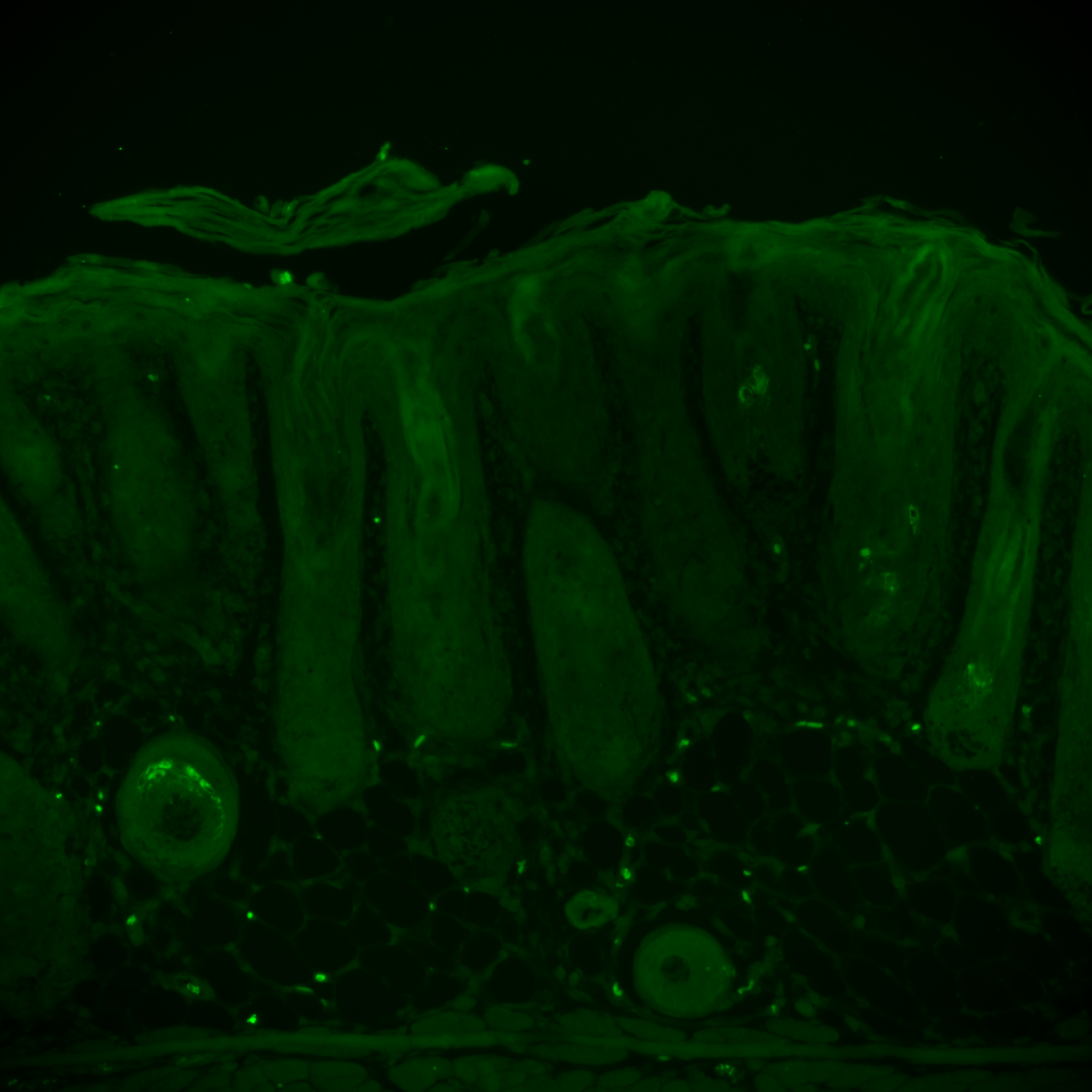

Supplement: Supplementary file 3 — Source data Fig. 2 [file 44318_2025_519_MOESM3_ESM.zip › Figure 2 Source Data/Fig. 2A SD/K86/P6 Gli2-3EKO K86.tif]

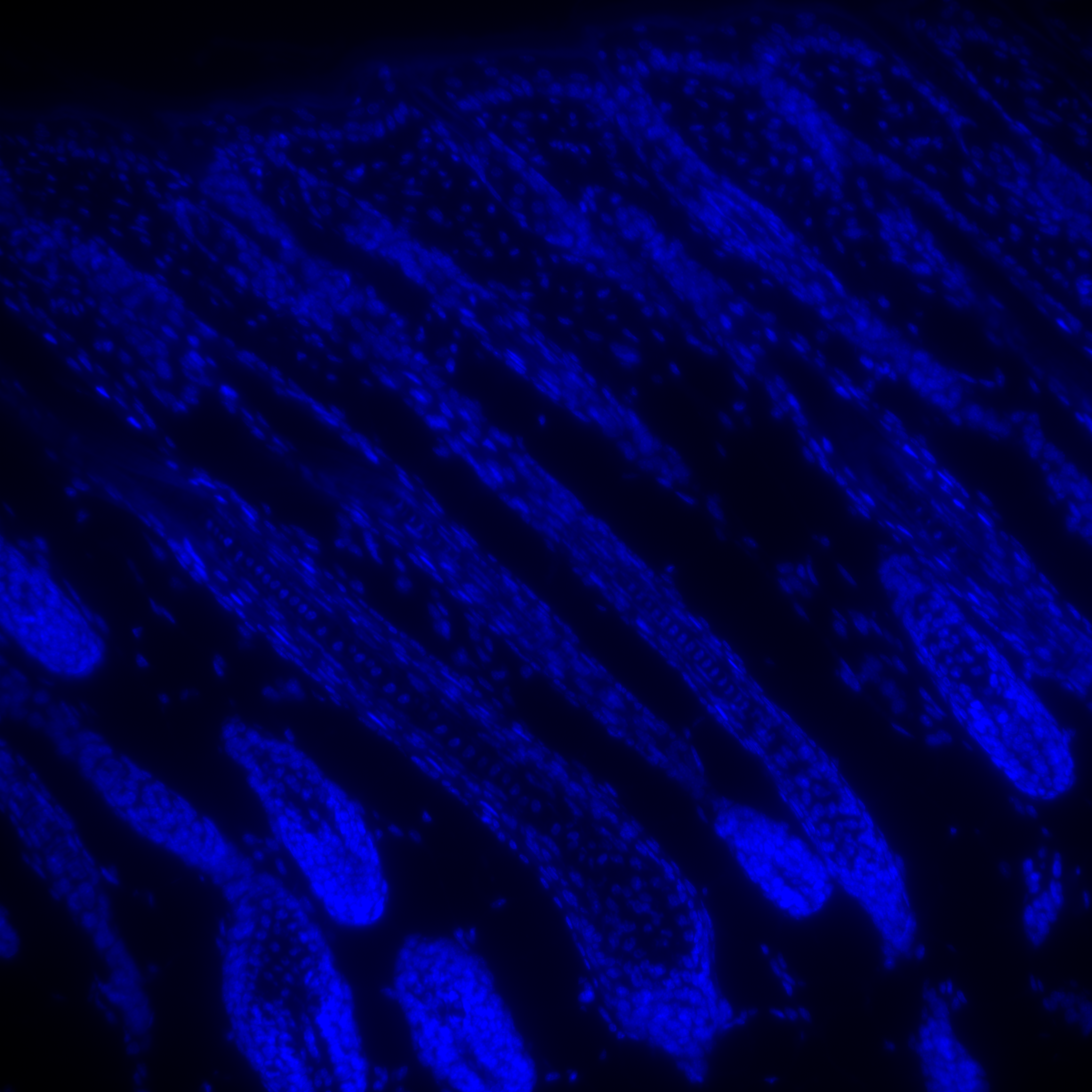

Supplement: Supplementary file 3 — Source data Fig. 2 [file 44318_2025_519_MOESM3_ESM.zip › Figure 2 Source Data/Fig. 2C SD/P6 Control DAPI.tif]

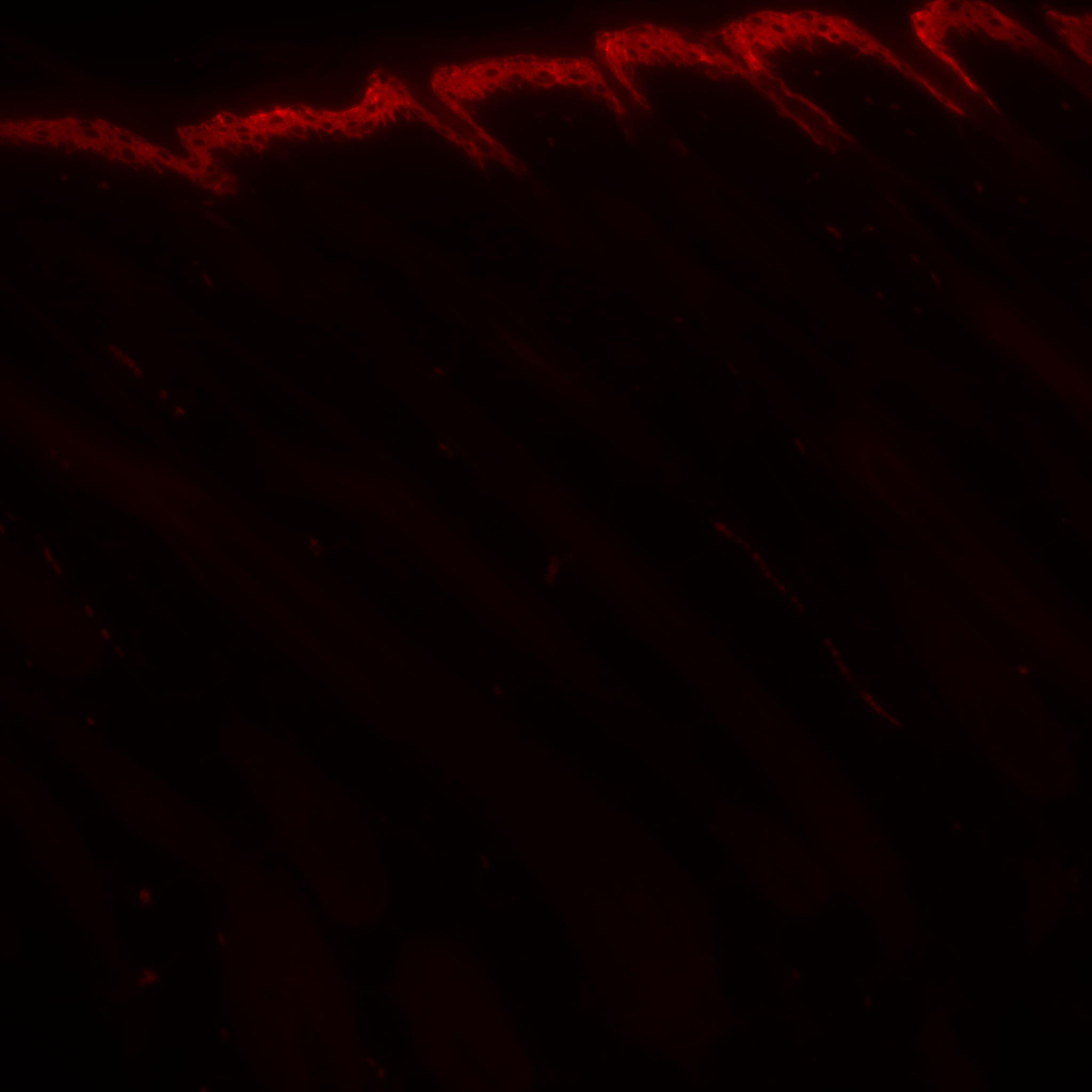

Supplement: Supplementary file 3 — Source data Fig. 2 [file 44318_2025_519_MOESM3_ESM.zip › Figure 2 Source Data/Fig. 2C SD/P6 Control K10.tif]

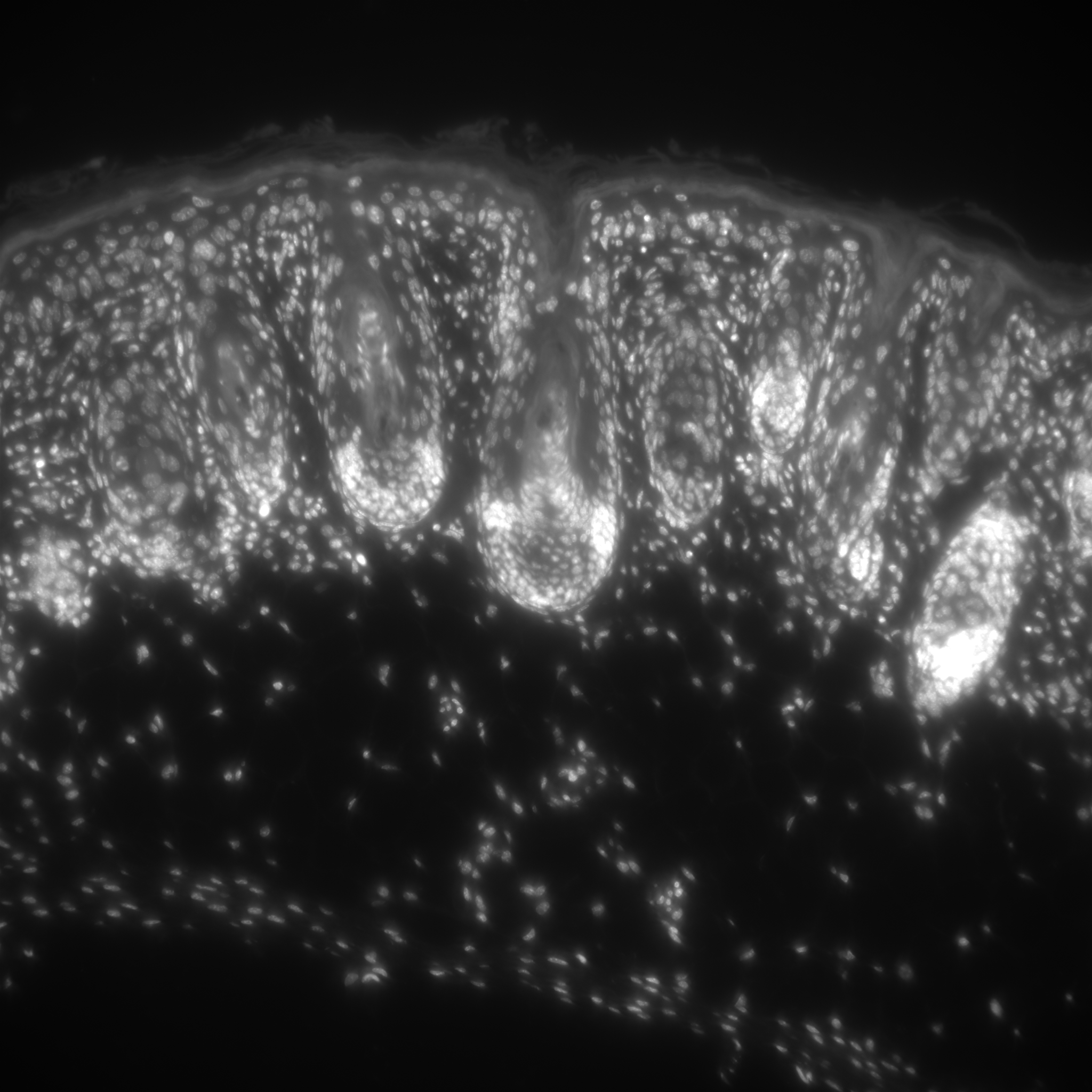

Supplement: Supplementary file 3 — Source data Fig. 2 [file 44318_2025_519_MOESM3_ESM.zip › Figure 2 Source Data/Fig. 2C SD/P6 Gli2-3EKO DAPI.tif]

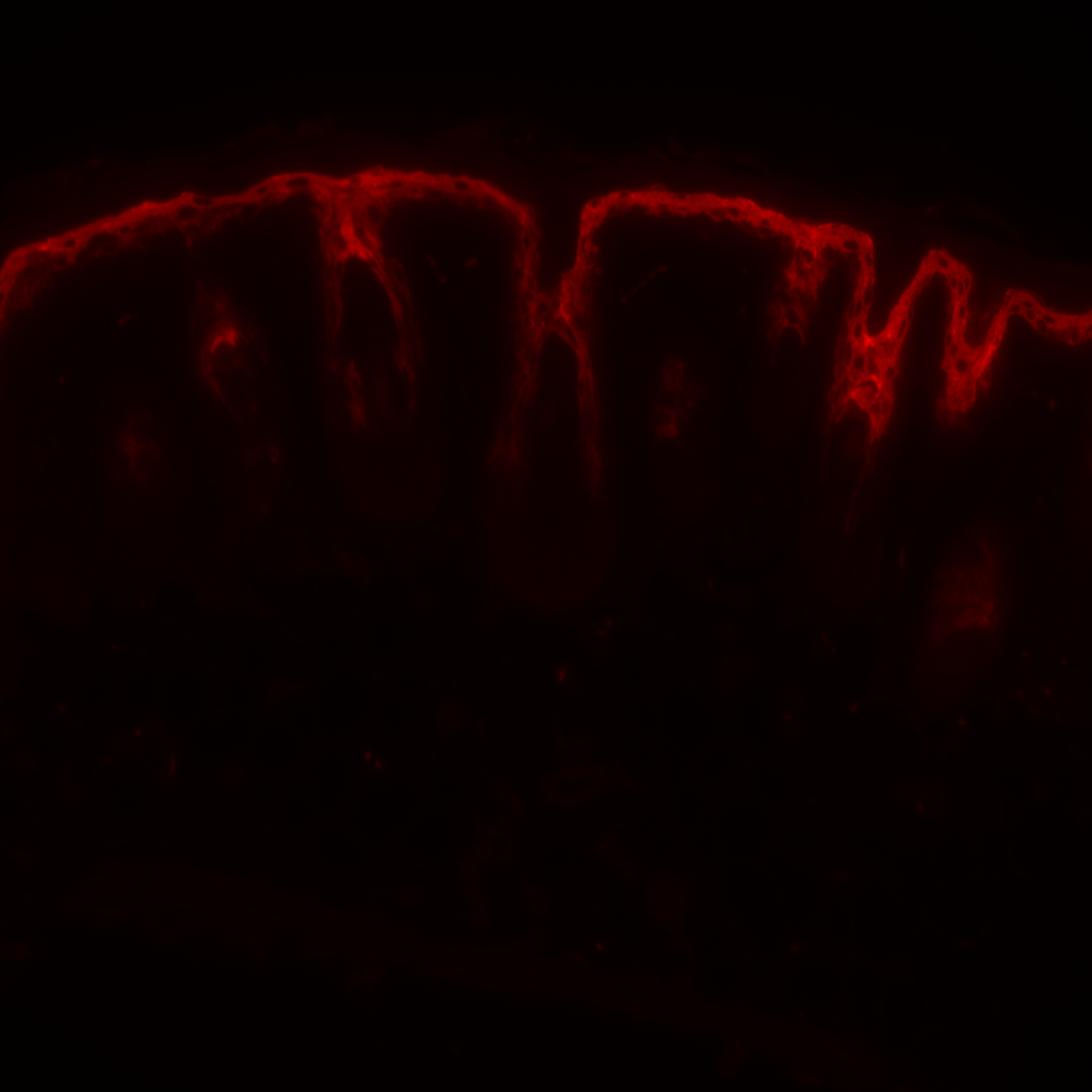

Supplement: Supplementary file 3 — Source data Fig. 2 [file 44318_2025_519_MOESM3_ESM.zip › Figure 2 Source Data/Fig. 2C SD/P6 Gli2-3EKO K10.tif]

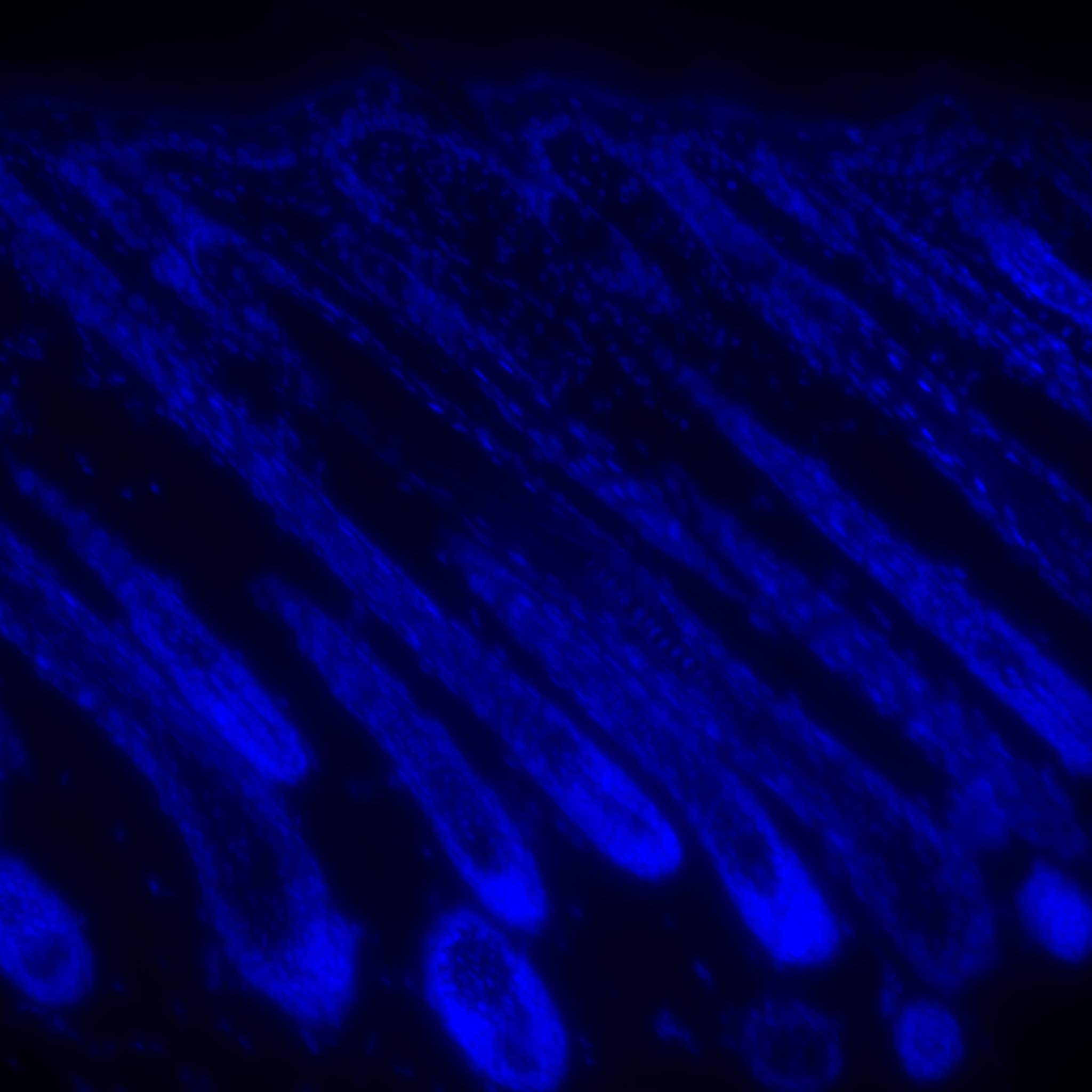

Supplement: Supplementary file 3 — Source data Fig. 2 [file 44318_2025_519_MOESM3_ESM.zip › Figure 2 Source Data/Fig. 2F SD/P6 Control DAPI.tif]

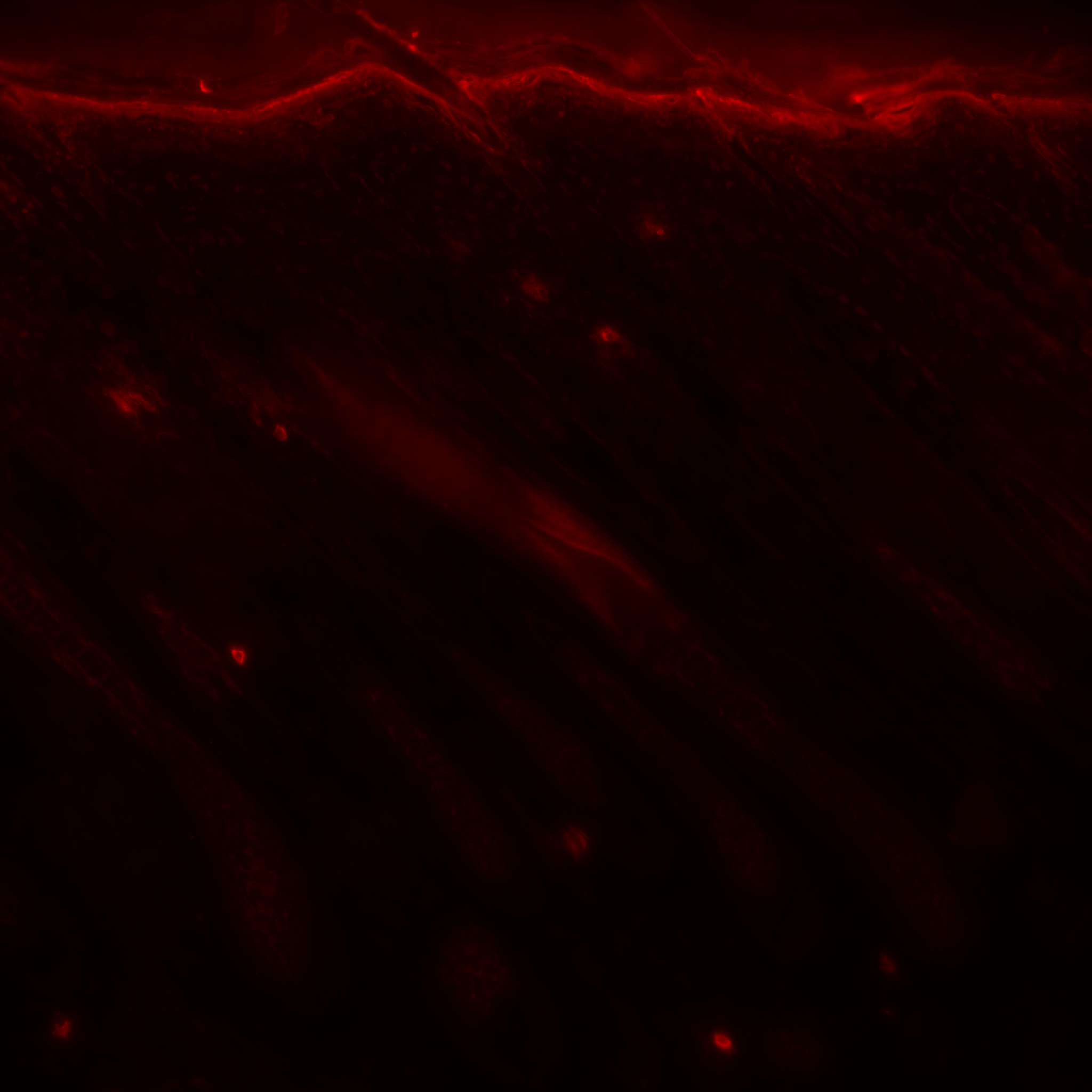

Supplement: Supplementary file 3 — Source data Fig. 2 [file 44318_2025_519_MOESM3_ESM.zip › Figure 2 Source Data/Fig. 2F SD/P6 Control Filaggrin.tif]

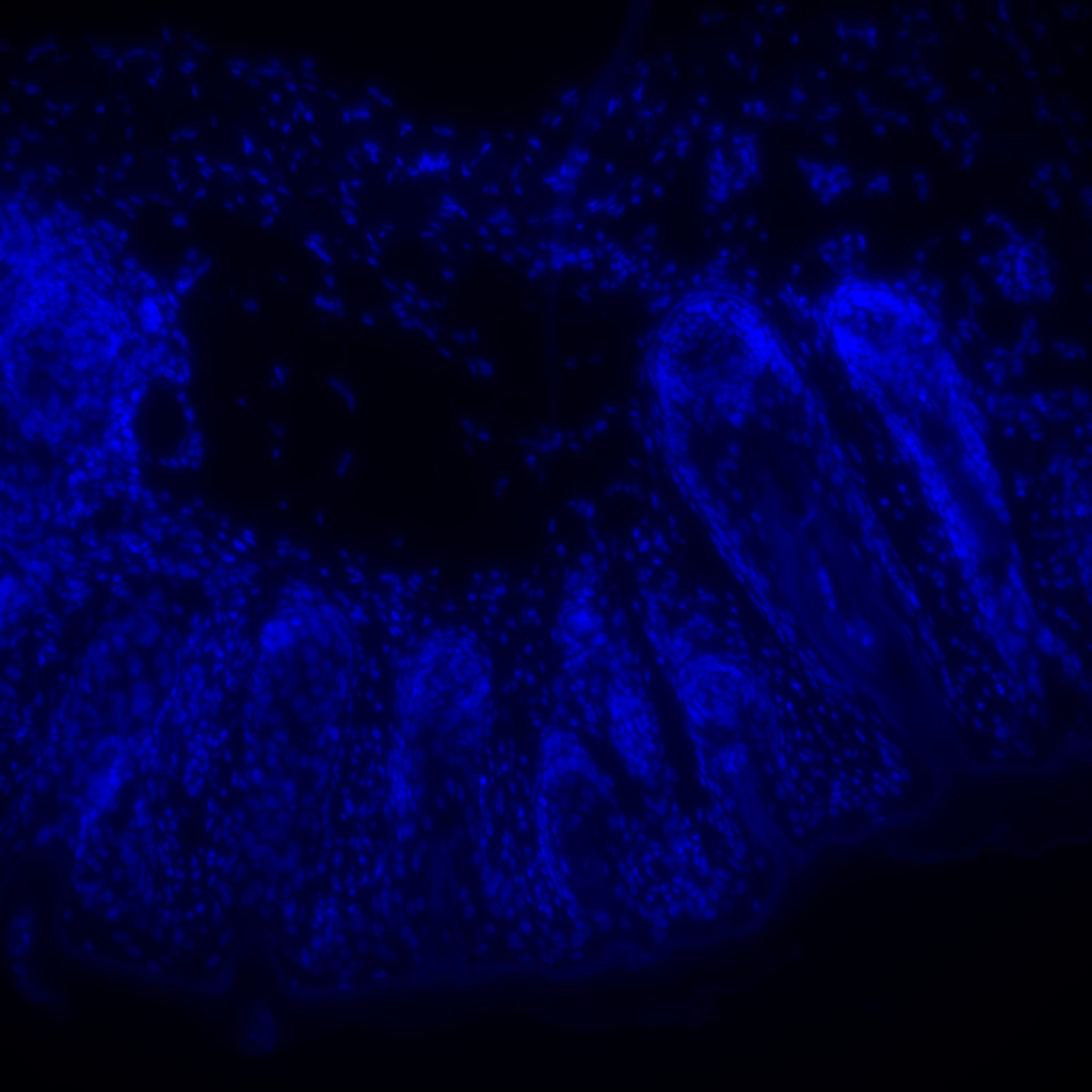

Supplement: Supplementary file 3 — Source data Fig. 2 [file 44318_2025_519_MOESM3_ESM.zip › Figure 2 Source Data/Fig. 2F SD/P6 Gli2-3EKO DAPI.tif]

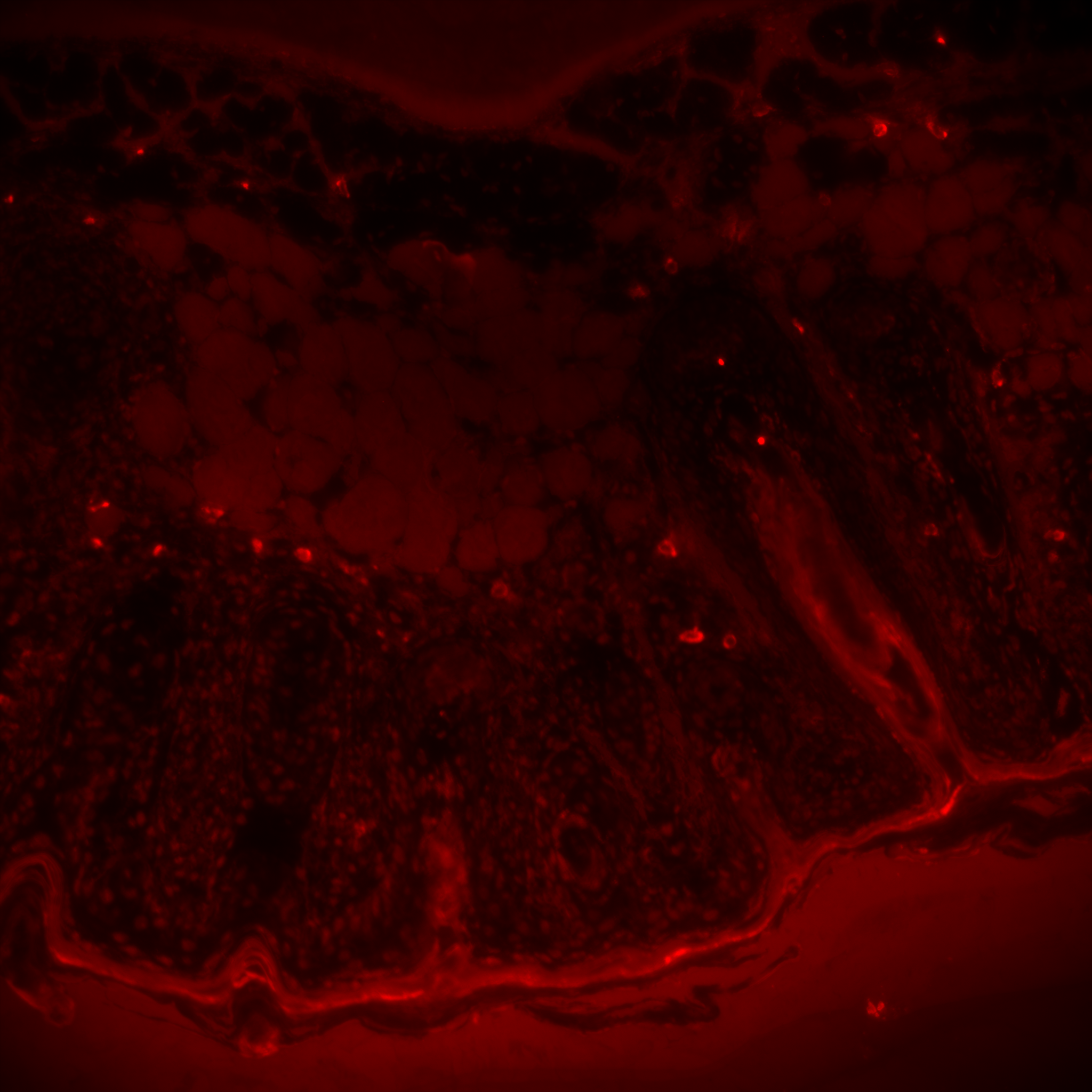

Supplement: Supplementary file 3 — Source data Fig. 2 [file 44318_2025_519_MOESM3_ESM.zip › Figure 2 Source Data/Fig. 2F SD/P6 Gli2-3EKO Filaggrin.tif]

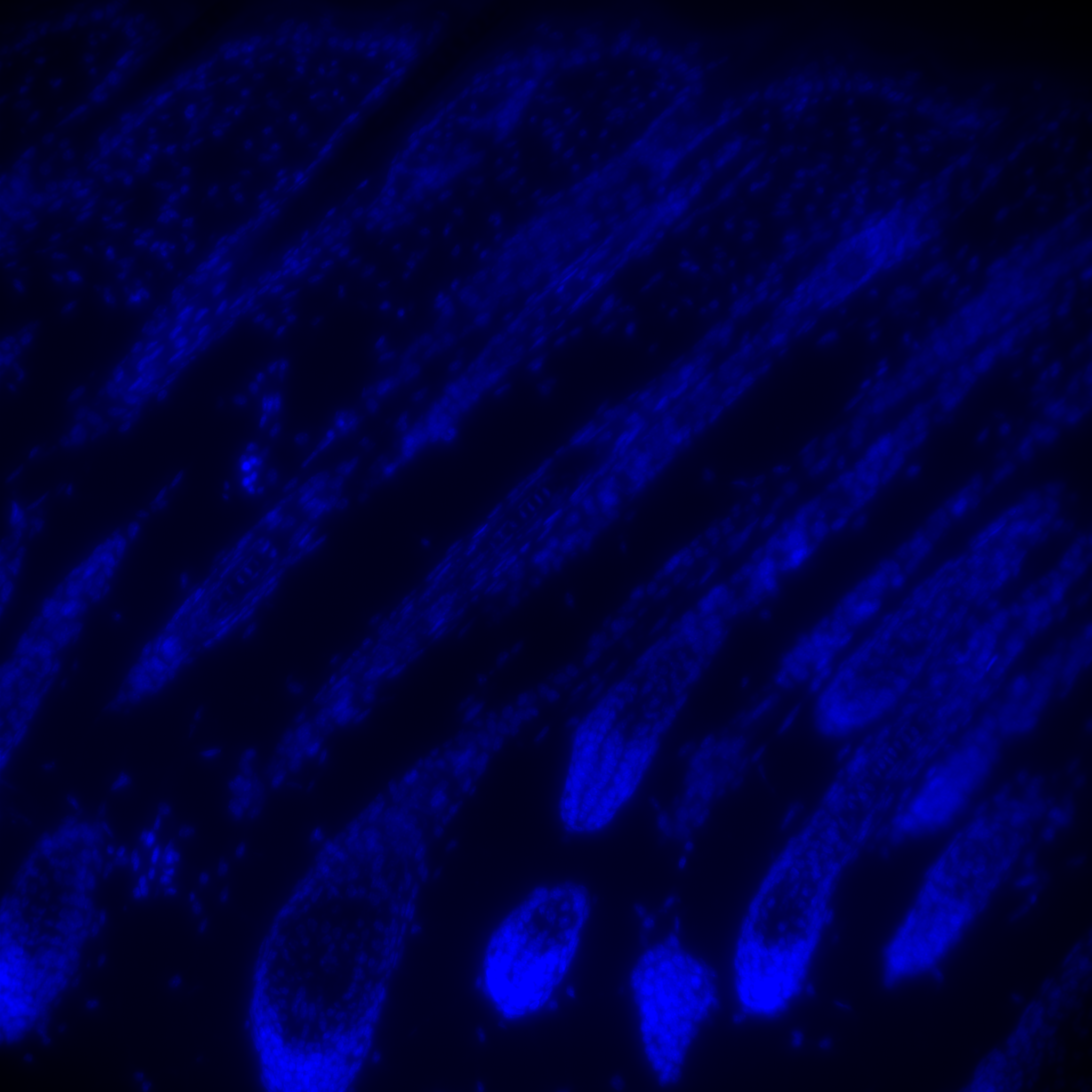

Supplement: Supplementary file 3 — Source data Fig. 2 [file 44318_2025_519_MOESM3_ESM.zip › Figure 2 Source Data/Fig. 2I SD/P6 Control DAPI.tif]

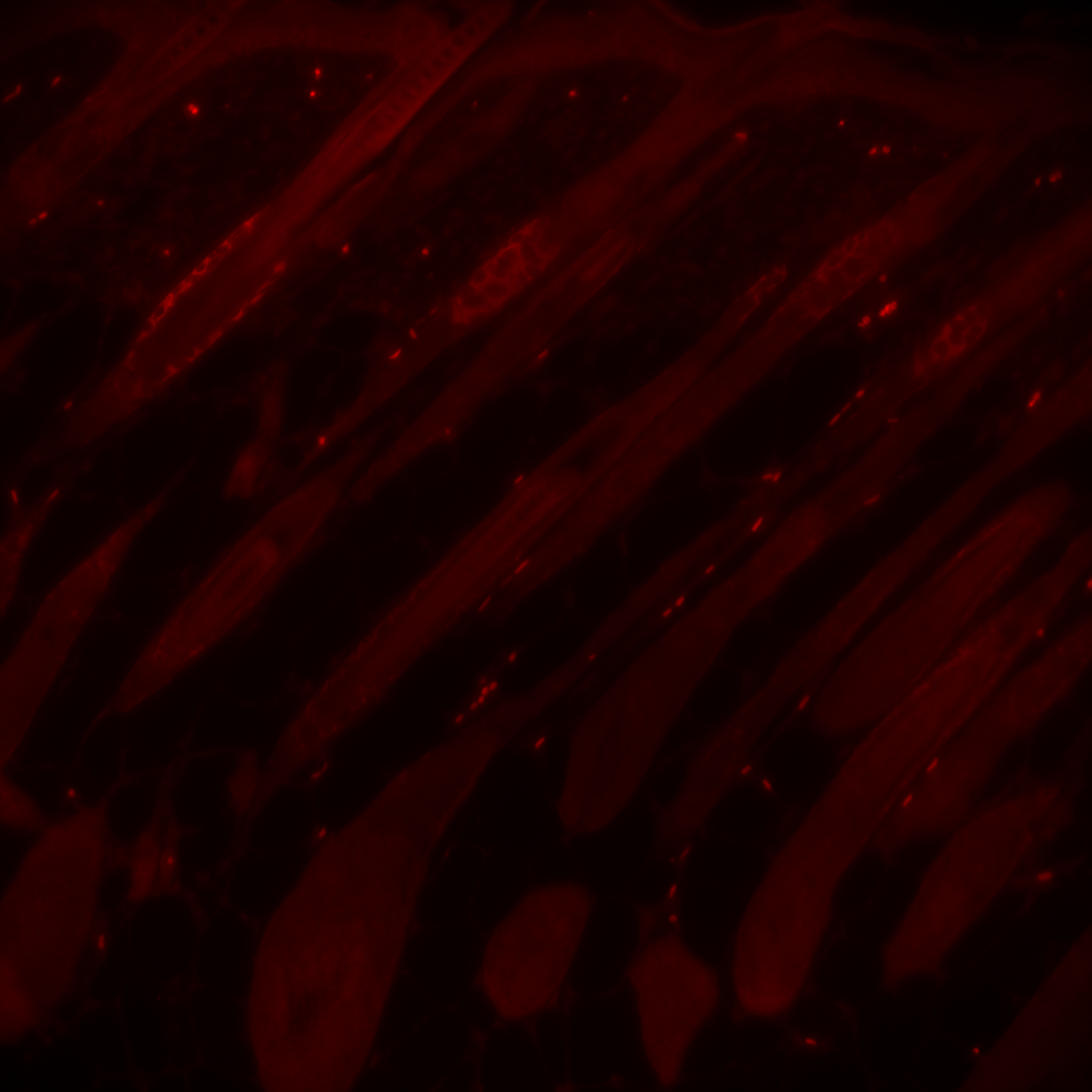

Supplement: Supplementary file 3 — Source data Fig. 2 [file 44318_2025_519_MOESM3_ESM.zip › Figure 2 Source Data/Fig. 2I SD/P6 Control K15.tif]

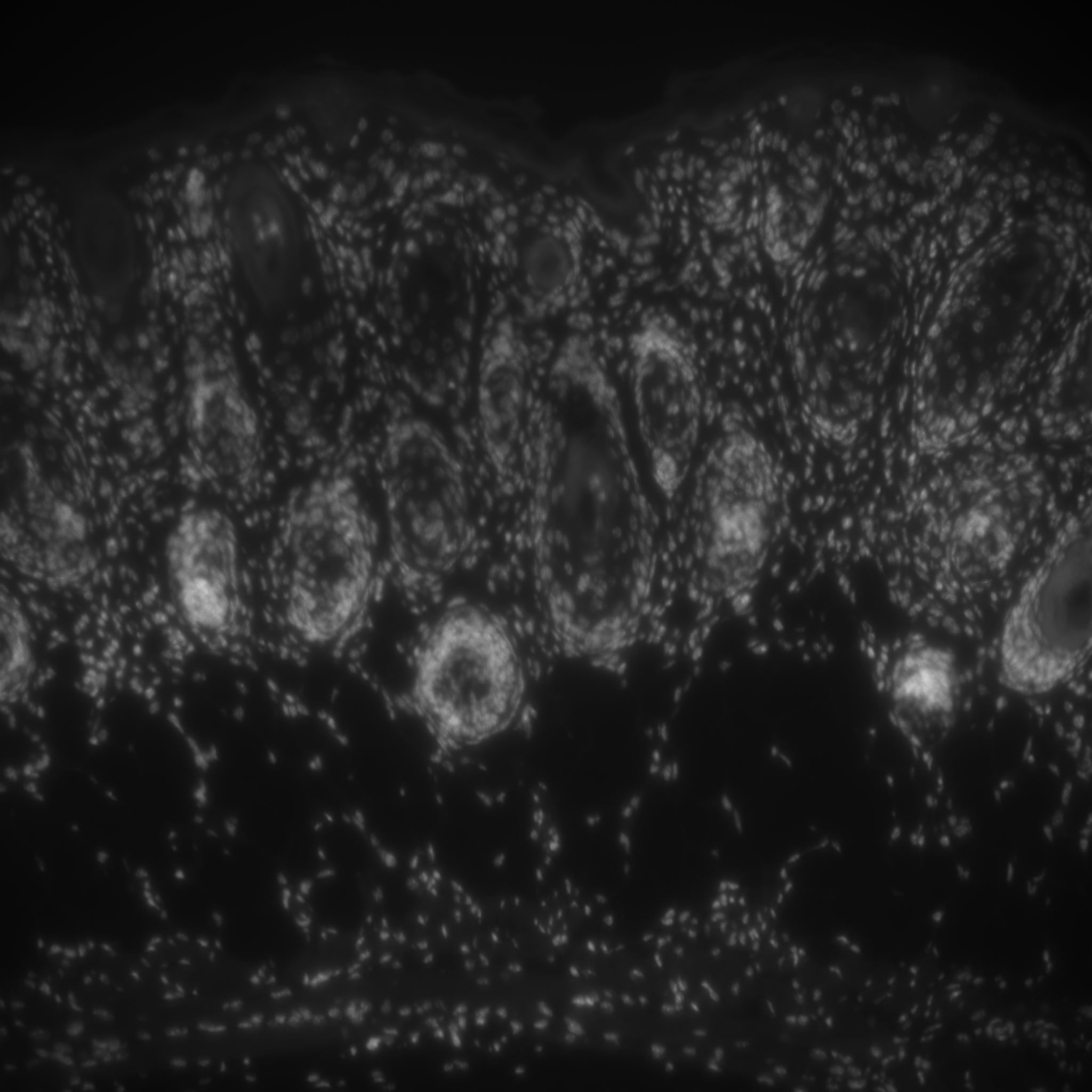

Supplement: Supplementary file 3 — Source data Fig. 2 [file 44318_2025_519_MOESM3_ESM.zip › Figure 2 Source Data/Fig. 2I SD/P6 Gli2-3EKO DAPI.tif]

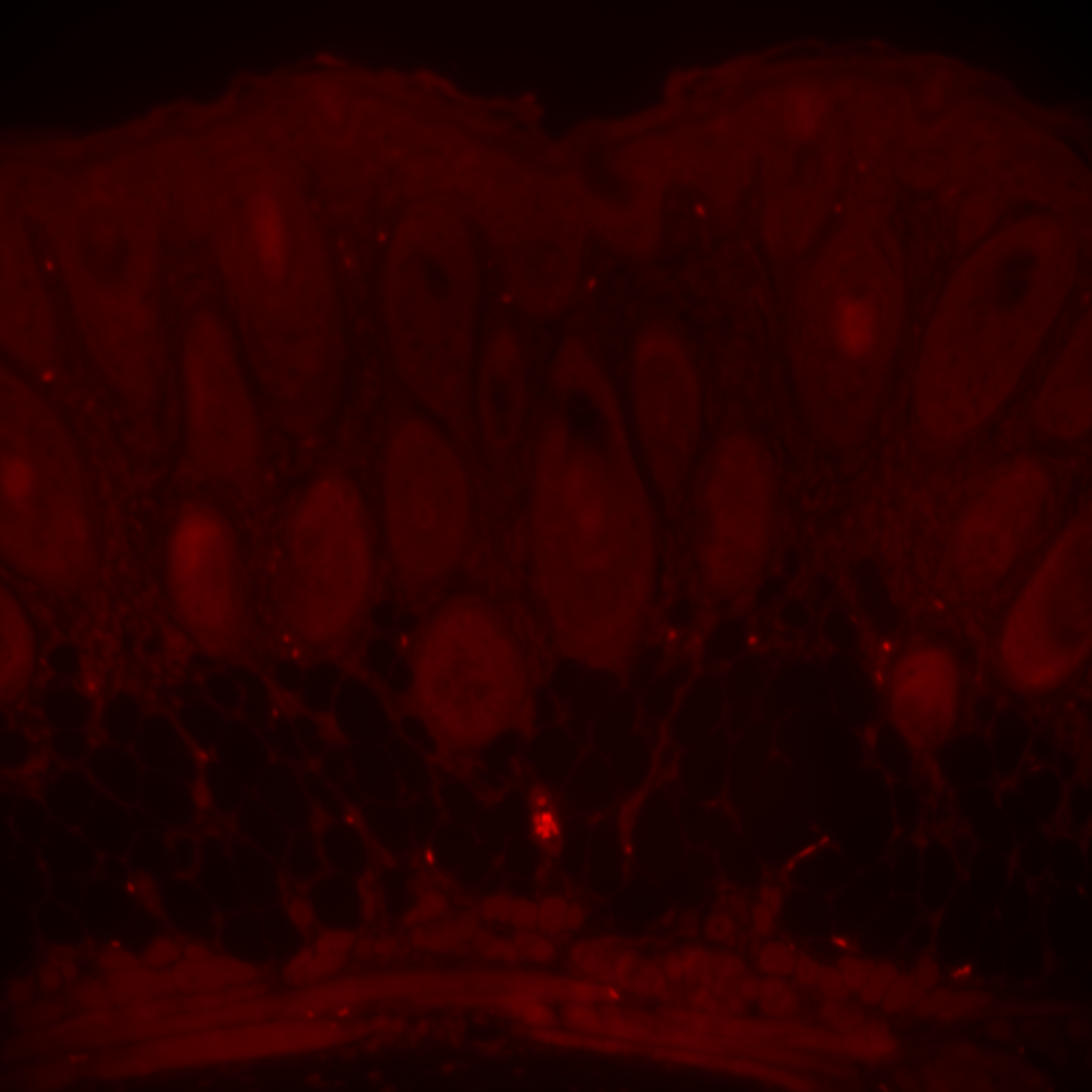

Supplement: Supplementary file 3 — Source data Fig. 2 [file 44318_2025_519_MOESM3_ESM.zip › Figure 2 Source Data/Fig. 2I SD/P6 Gli2-3EKO K15.tif]

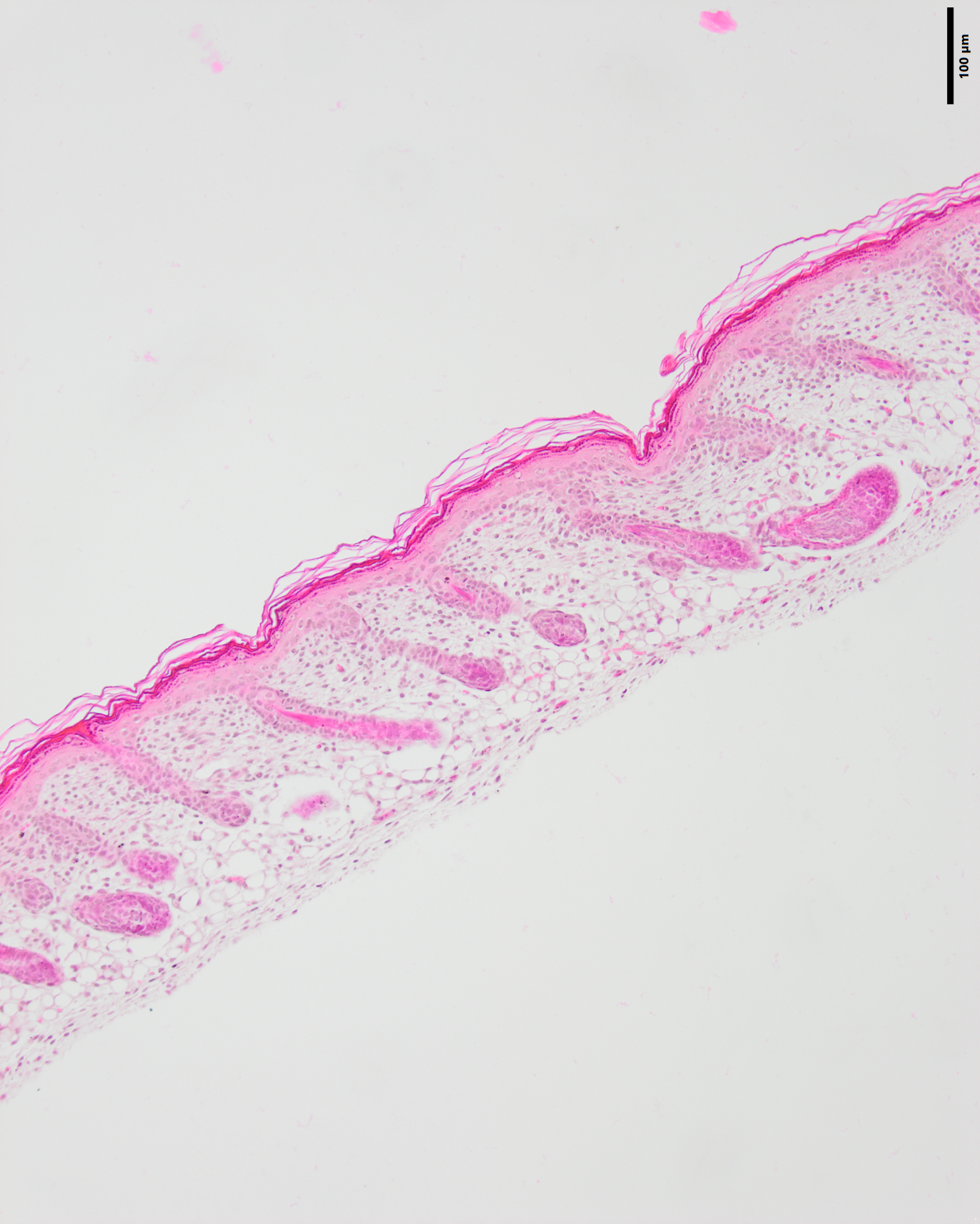

Supplement: Supplementary file 4 — Source data Fig. 3 [file 44318_2025_519_MOESM4_ESM.zip › Figure 3 Source Data/Fig. 3A SD/P6 Control.tif]

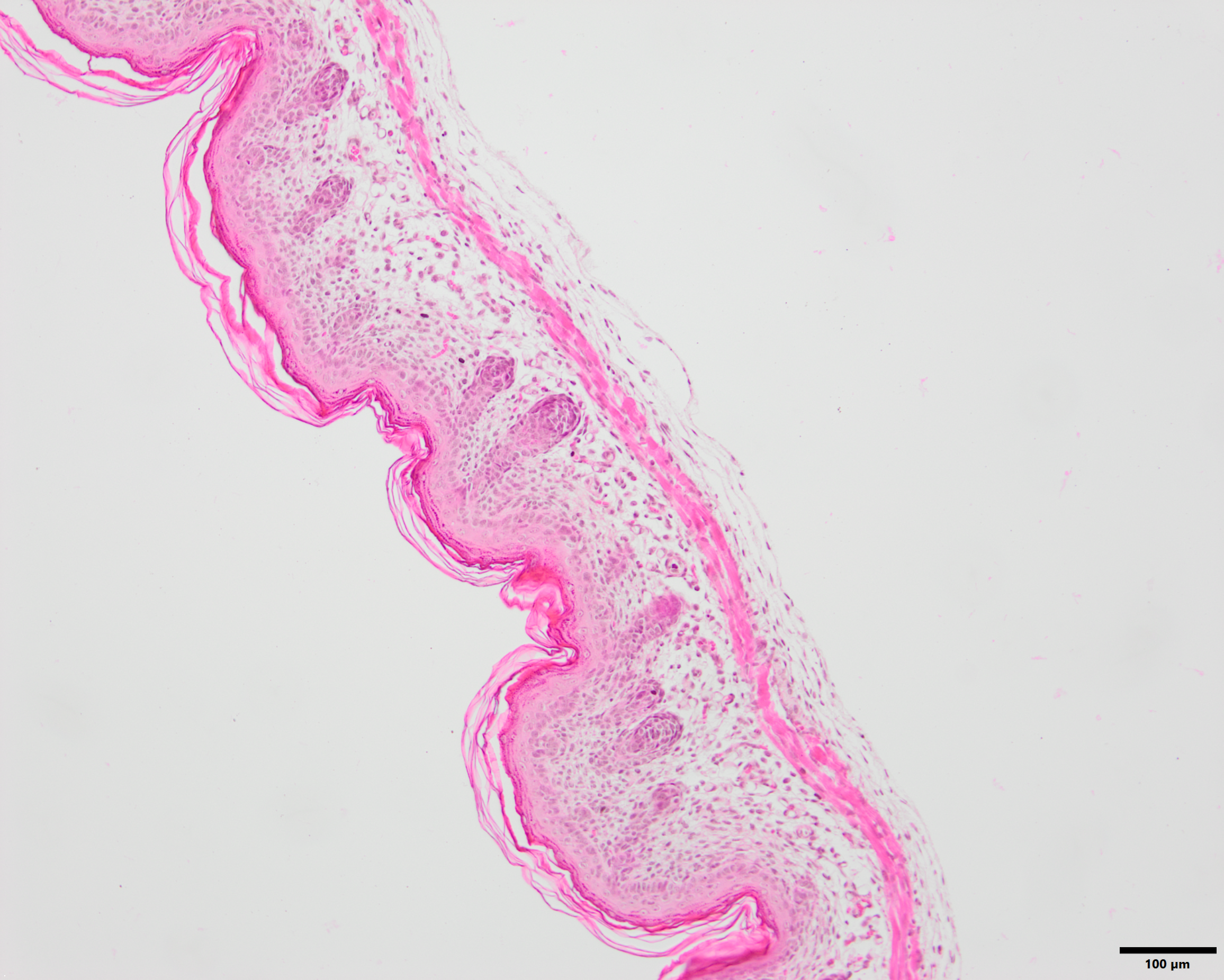

Supplement: Supplementary file 4 — Source data Fig. 3 [file 44318_2025_519_MOESM4_ESM.zip › Figure 3 Source Data/Fig. 3A SD/P6 Ift88EKO.tif]

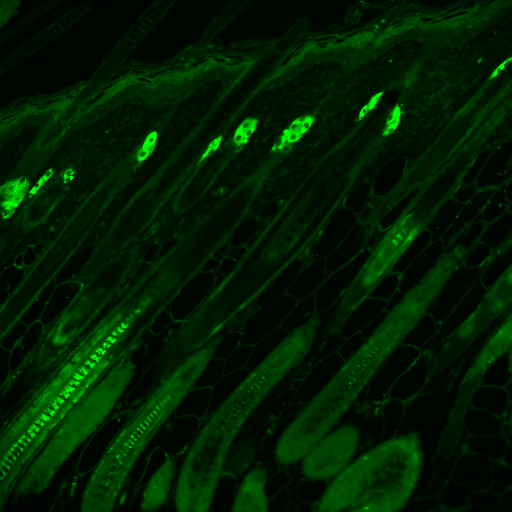

Supplement: Supplementary file 4 — Source data Fig. 3 [file 44318_2025_519_MOESM4_ESM.zip › Figure 3 Source Data/Fig. 3C SD/P6 Control Adipophilin.tif]

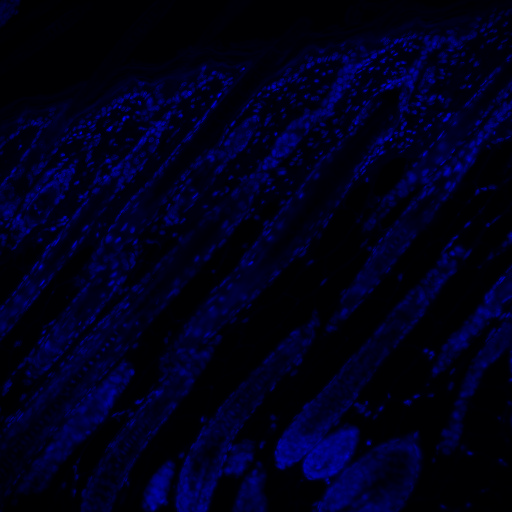

Supplement: Supplementary file 4 — Source data Fig. 3 [file 44318_2025_519_MOESM4_ESM.zip › Figure 3 Source Data/Fig. 3C SD/P6 Control DAPI.tif]

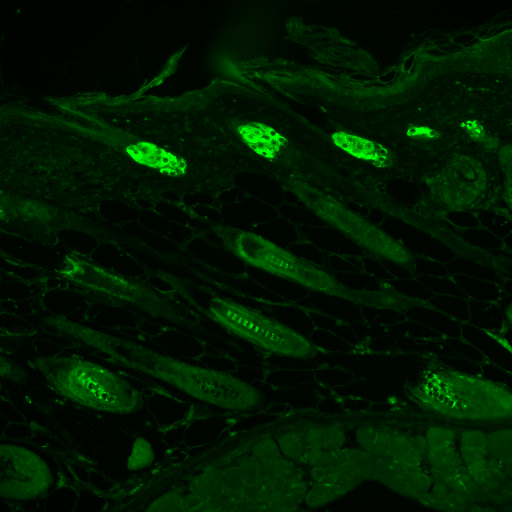

Supplement: Supplementary file 4 — Source data Fig. 3 [file 44318_2025_519_MOESM4_ESM.zip › Figure 3 Source Data/Fig. 3C SD/P6 Gli2EKO Adipophilin.tif]

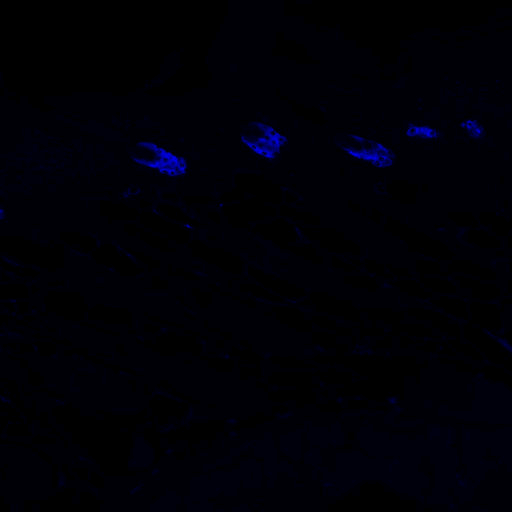

Supplement: Supplementary file 4 — Source data Fig. 3 [file 44318_2025_519_MOESM4_ESM.zip › Figure 3 Source Data/Fig. 3C SD/P6 Gli2EKO DAPI.tif]

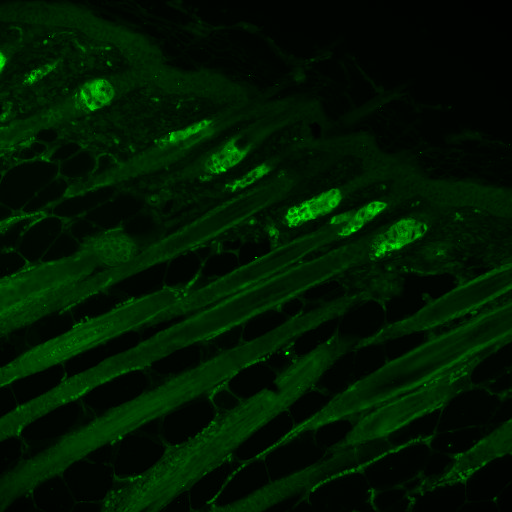

Supplement: Supplementary file 4 — Source data Fig. 3 [file 44318_2025_519_MOESM4_ESM.zip › Figure 3 Source Data/Fig. 3C SD/P6 Ift88EKO Adipophilin.tif]

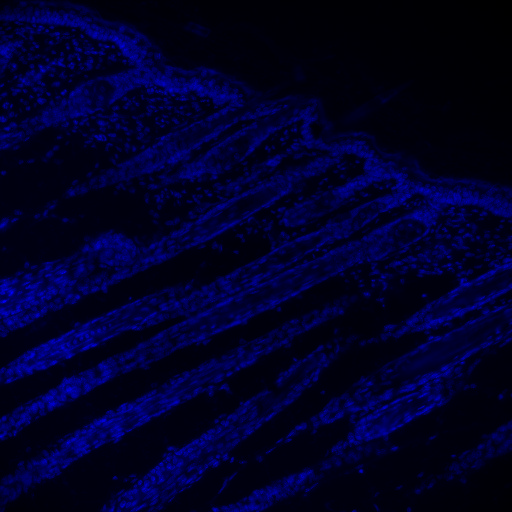

Supplement: Supplementary file 4 — Source data Fig. 3 [file 44318_2025_519_MOESM4_ESM.zip › Figure 3 Source Data/Fig. 3C SD/P6 Ift88EKO DAPI.tif]

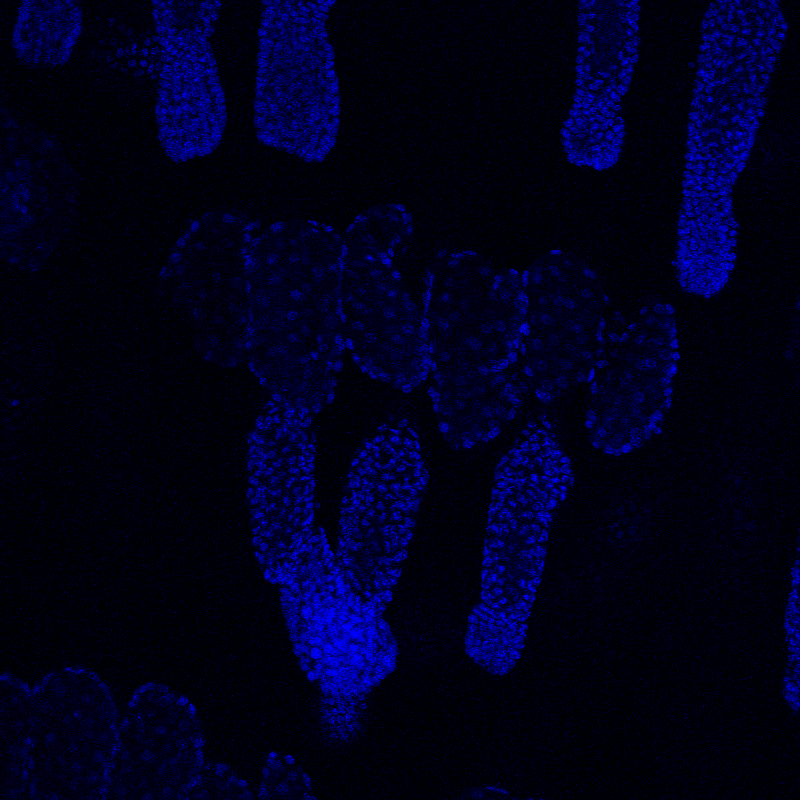

Supplement: Supplementary file 4 — Source data Fig. 3 [file 44318_2025_519_MOESM4_ESM.zip › Figure 3 Source Data/Fig. 3D SD/P49 Control DAPI.tif]

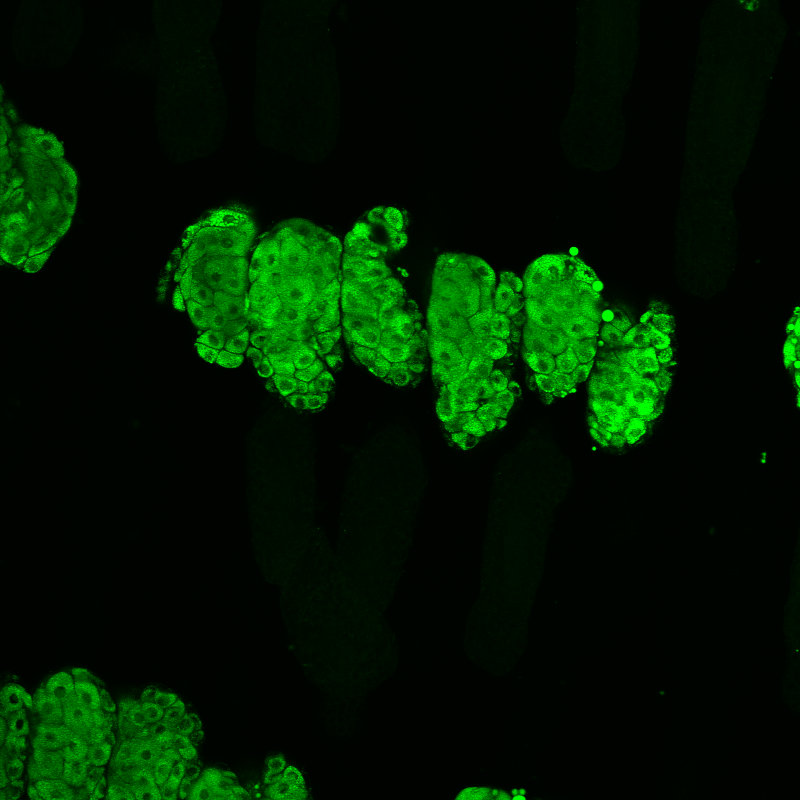

Supplement: Supplementary file 4 — Source data Fig. 3 [file 44318_2025_519_MOESM4_ESM.zip › Figure 3 Source Data/Fig. 3D SD/P49 Control Nile red - green.tif]

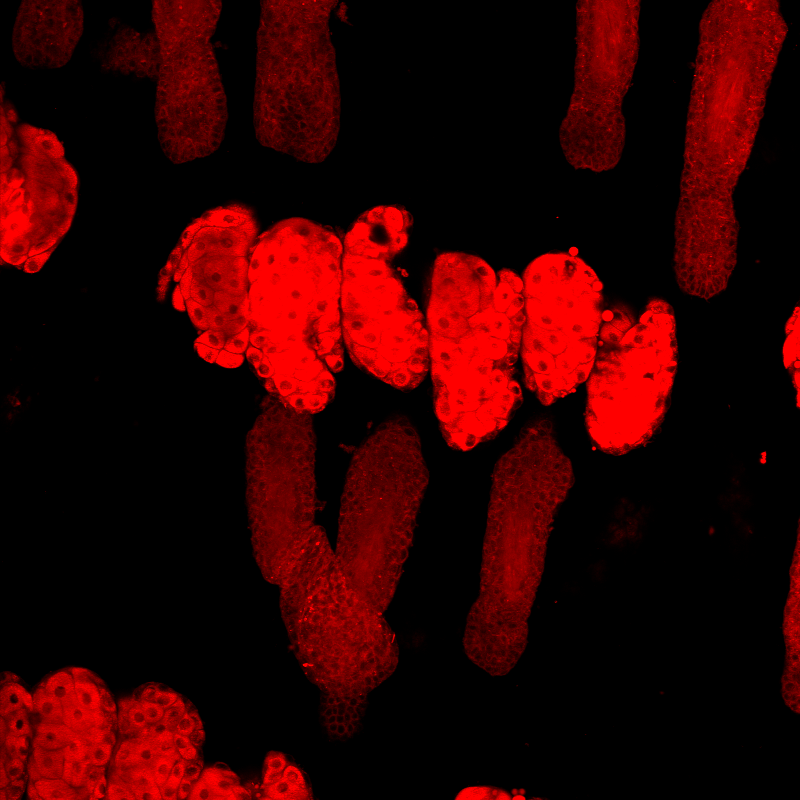

Supplement: Supplementary file 4 — Source data Fig. 3 [file 44318_2025_519_MOESM4_ESM.zip › Figure 3 Source Data/Fig. 3D SD/P49 Control Nile red- red.tif]

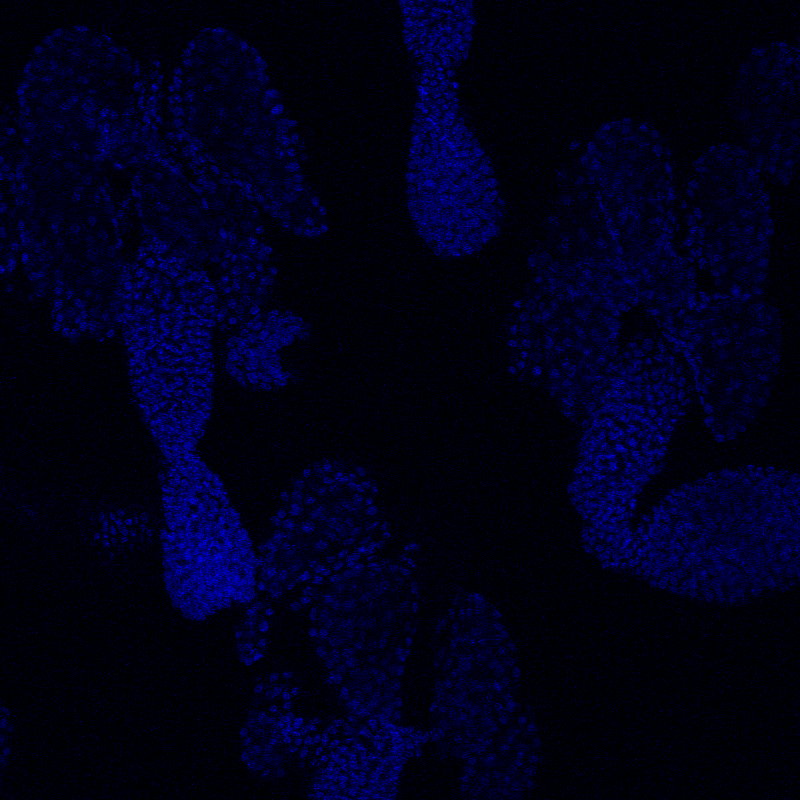

Supplement: Supplementary file 4 — Source data Fig. 3 [file 44318_2025_519_MOESM4_ESM.zip › Figure 3 Source Data/Fig. 3D SD/P49 Gli2EKO DAPI.tif]

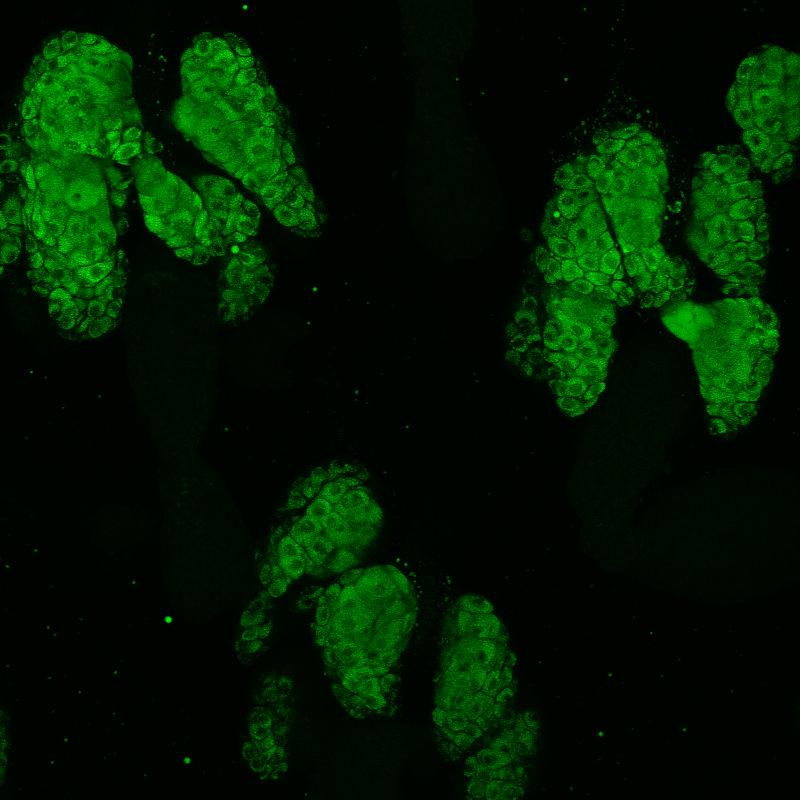

Supplement: Supplementary file 4 — Source data Fig. 3 [file 44318_2025_519_MOESM4_ESM.zip › Figure 3 Source Data/Fig. 3D SD/P49 Gli2EKO Nile red - green.tif]

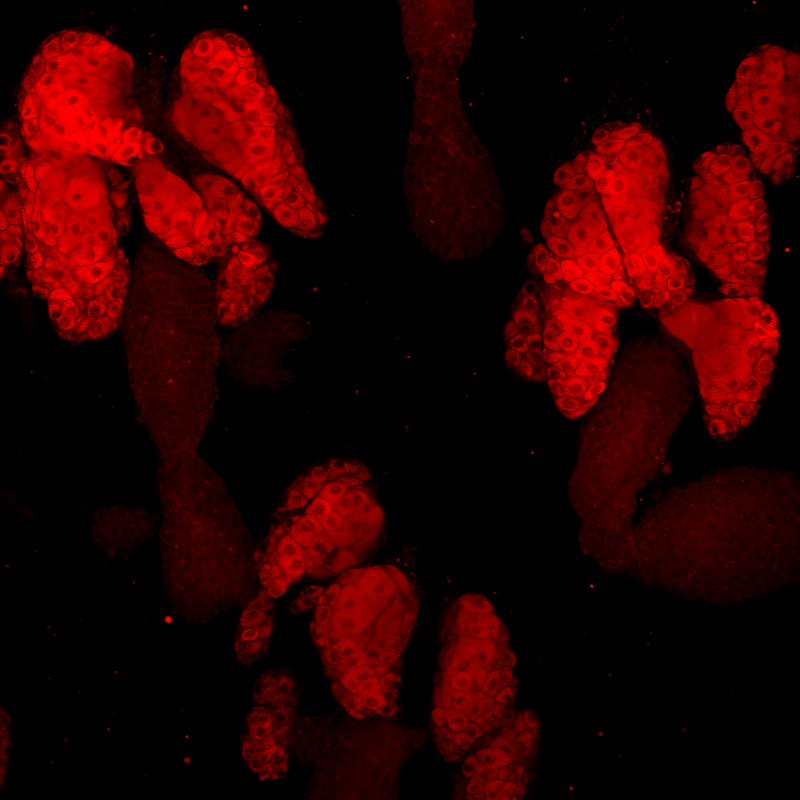

Supplement: Supplementary file 4 — Source data Fig. 3 [file 44318_2025_519_MOESM4_ESM.zip › Figure 3 Source Data/Fig. 3D SD/P49 Gli2EKO Nile red - red.tif]

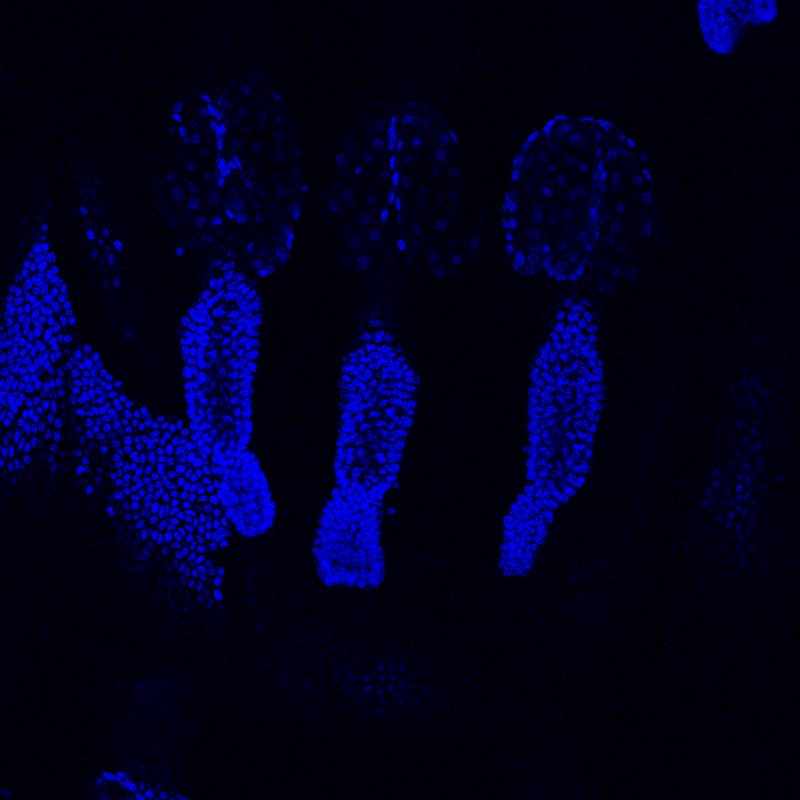

Supplement: Supplementary file 4 — Source data Fig. 3 [file 44318_2025_519_MOESM4_ESM.zip › Figure 3 Source Data/Fig. 3D SD/P49 Gli3EKO DAPI.tif]

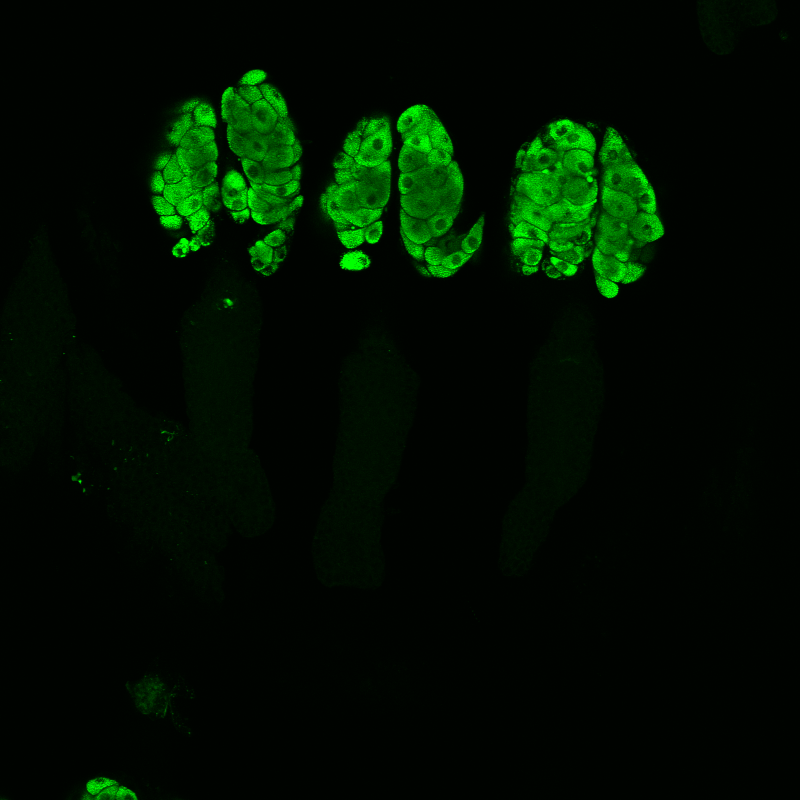

Supplement: Supplementary file 4 — Source data Fig. 3 [file 44318_2025_519_MOESM4_ESM.zip › Figure 3 Source Data/Fig. 3D SD/P49 Gli3EKO Nile red - green.tif]

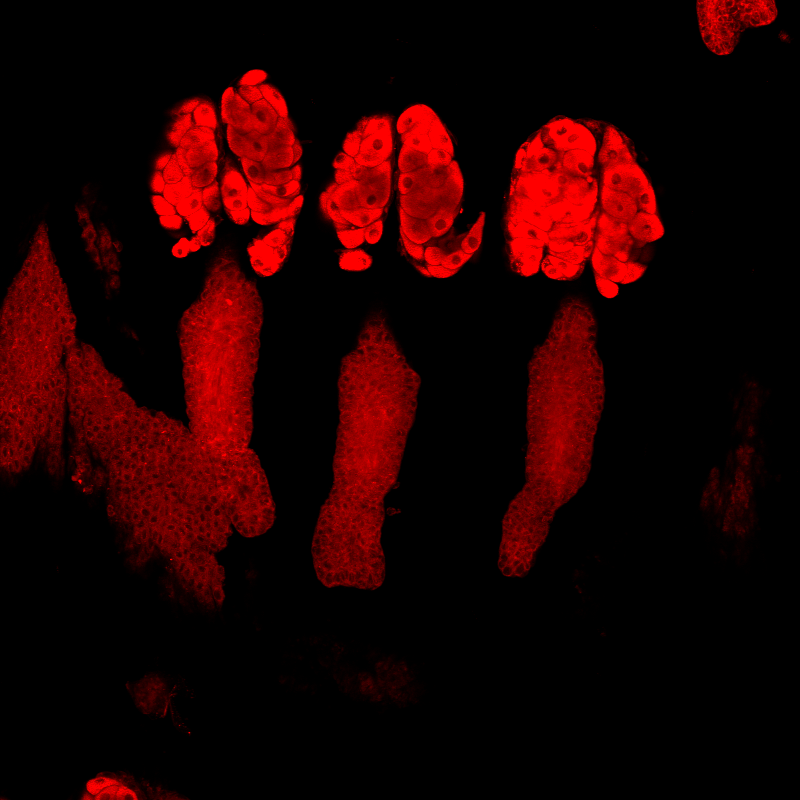

Supplement: Supplementary file 4 — Source data Fig. 3 [file 44318_2025_519_MOESM4_ESM.zip › Figure 3 Source Data/Fig. 3D SD/P49 Gli3EKO Nile red- red.tif]

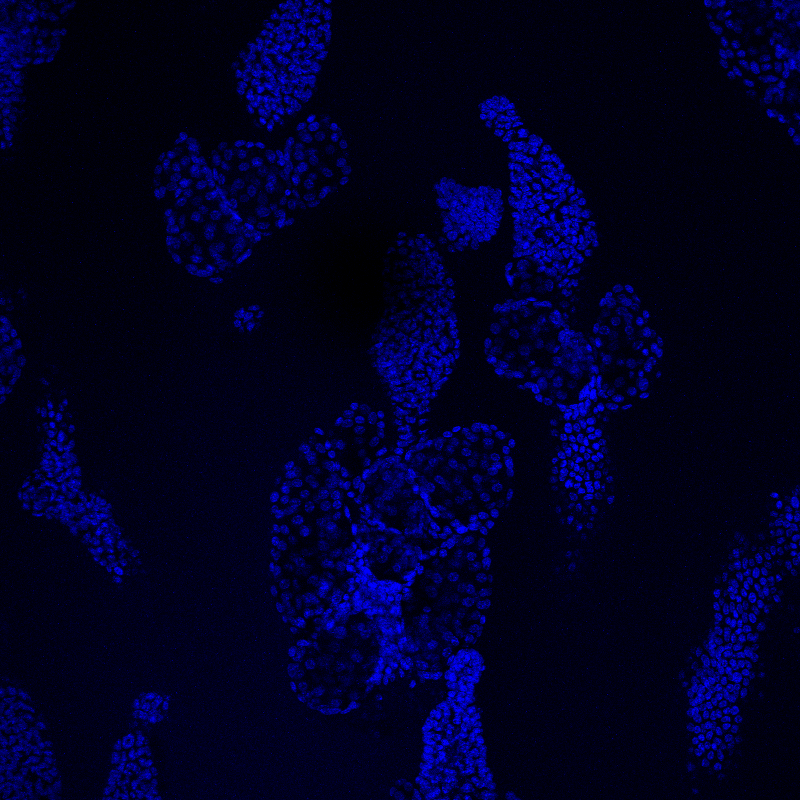

Supplement: Supplementary file 4 — Source data Fig. 3 [file 44318_2025_519_MOESM4_ESM.zip › Figure 3 Source Data/Fig. 3D SD/P49 Ift88EKO DAPI.tif]

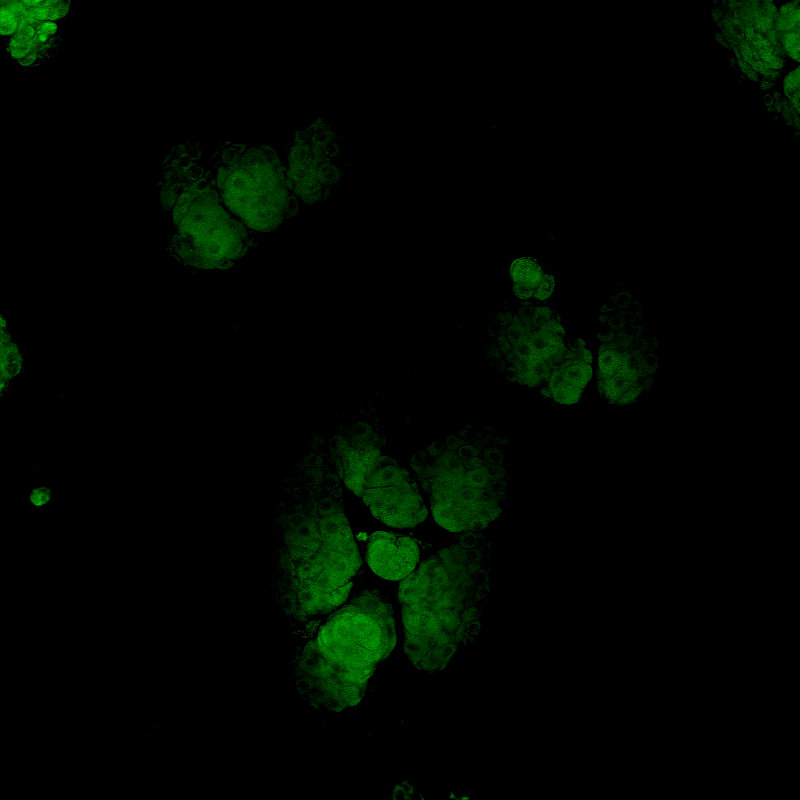

Supplement: Supplementary file 4 — Source data Fig. 3 [file 44318_2025_519_MOESM4_ESM.zip › Figure 3 Source Data/Fig. 3D SD/P49 Ift88EKO Nile red - green.tif]

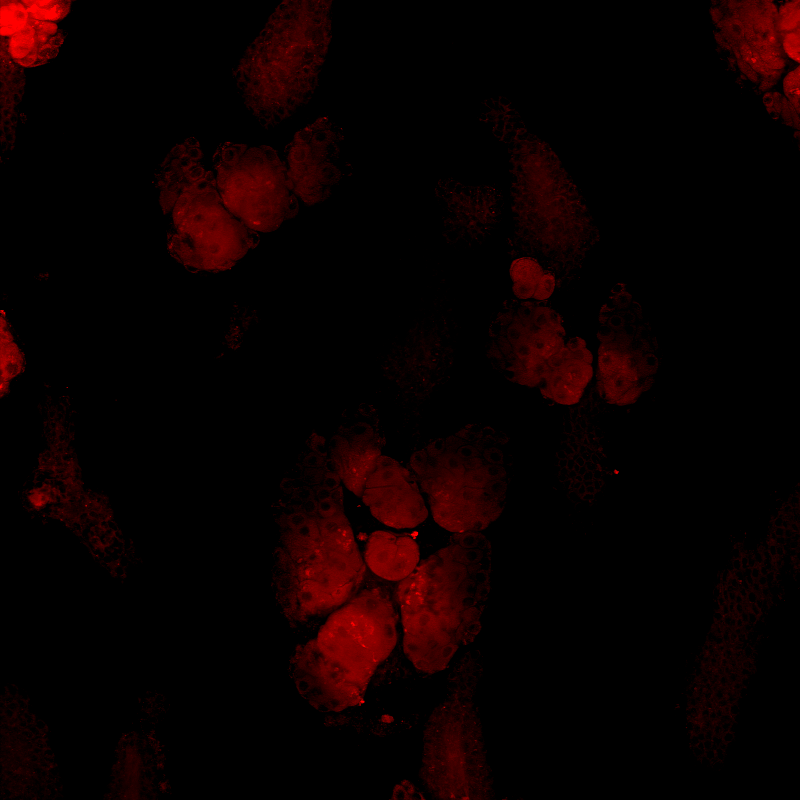

Supplement: Supplementary file 4 — Source data Fig. 3 [file 44318_2025_519_MOESM4_ESM.zip › Figure 3 Source Data/Fig. 3D SD/P49 Ift88EKO Nile red - red.tif]

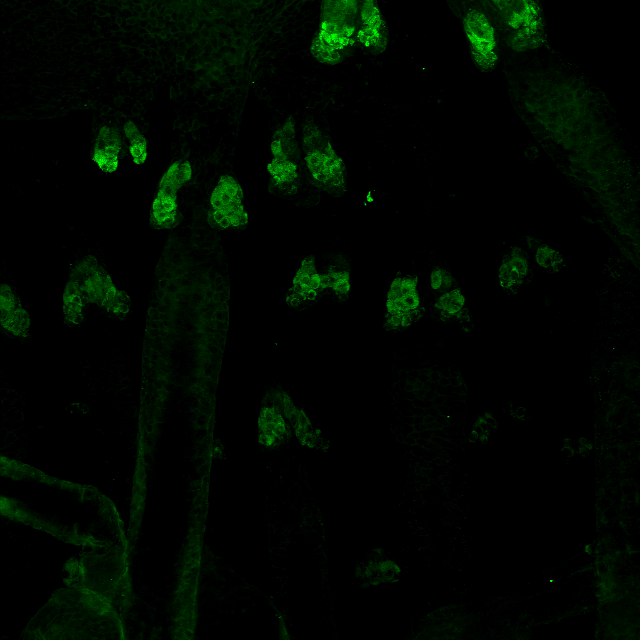

Supplement: Supplementary file 5 — Source data Fig. 4 [file 44318_2025_519_MOESM5_ESM.zip › Figure 4 Source Data/Fig. 4A SD/P6 Control Adipophilin.tif]

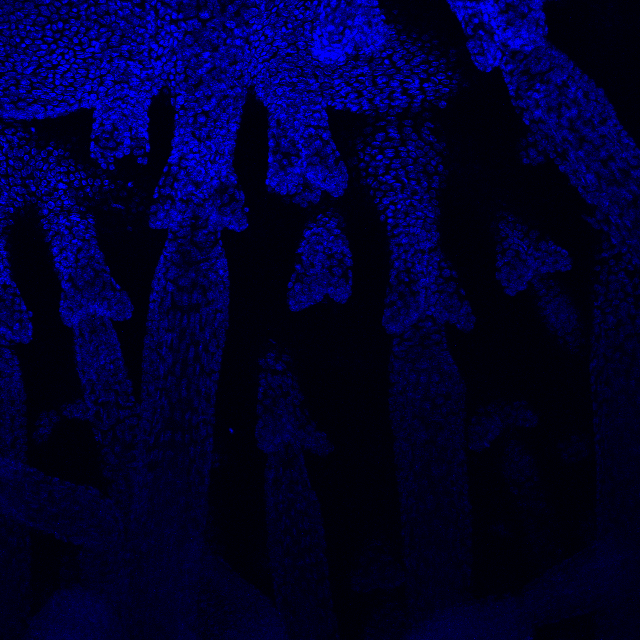

Supplement: Supplementary file 5 — Source data Fig. 4 [file 44318_2025_519_MOESM5_ESM.zip › Figure 4 Source Data/Fig. 4A SD/P6 Control DAPI.tif]

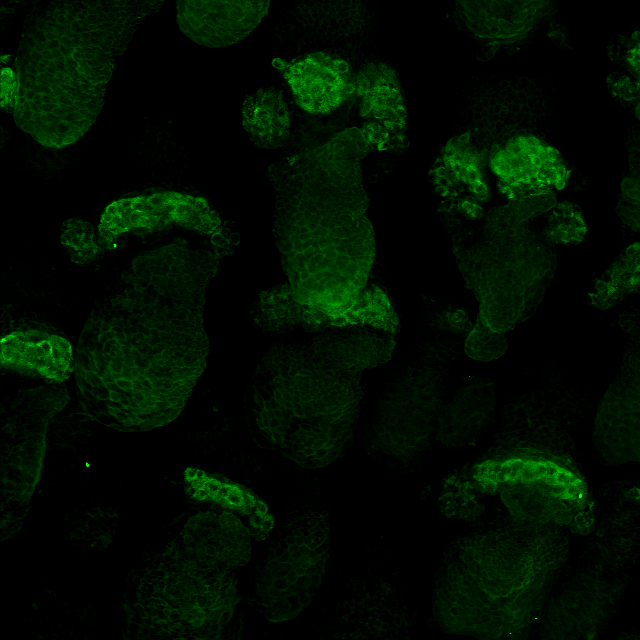

Supplement: Supplementary file 5 — Source data Fig. 4 [file 44318_2025_519_MOESM5_ESM.zip › Figure 4 Source Data/Fig. 4A SD/P6 Gli2EKO Adipophilin.tif]

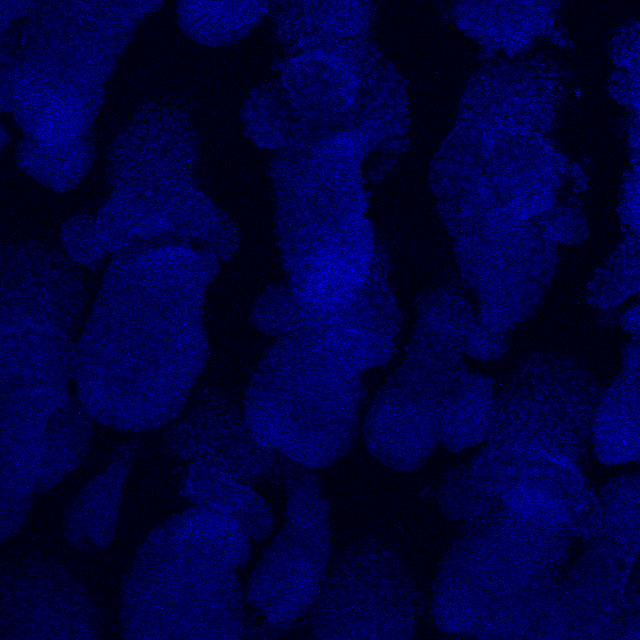

Supplement: Supplementary file 5 — Source data Fig. 4 [file 44318_2025_519_MOESM5_ESM.zip › Figure 4 Source Data/Fig. 4A SD/P6 Gli2EKO DAPI.tif]

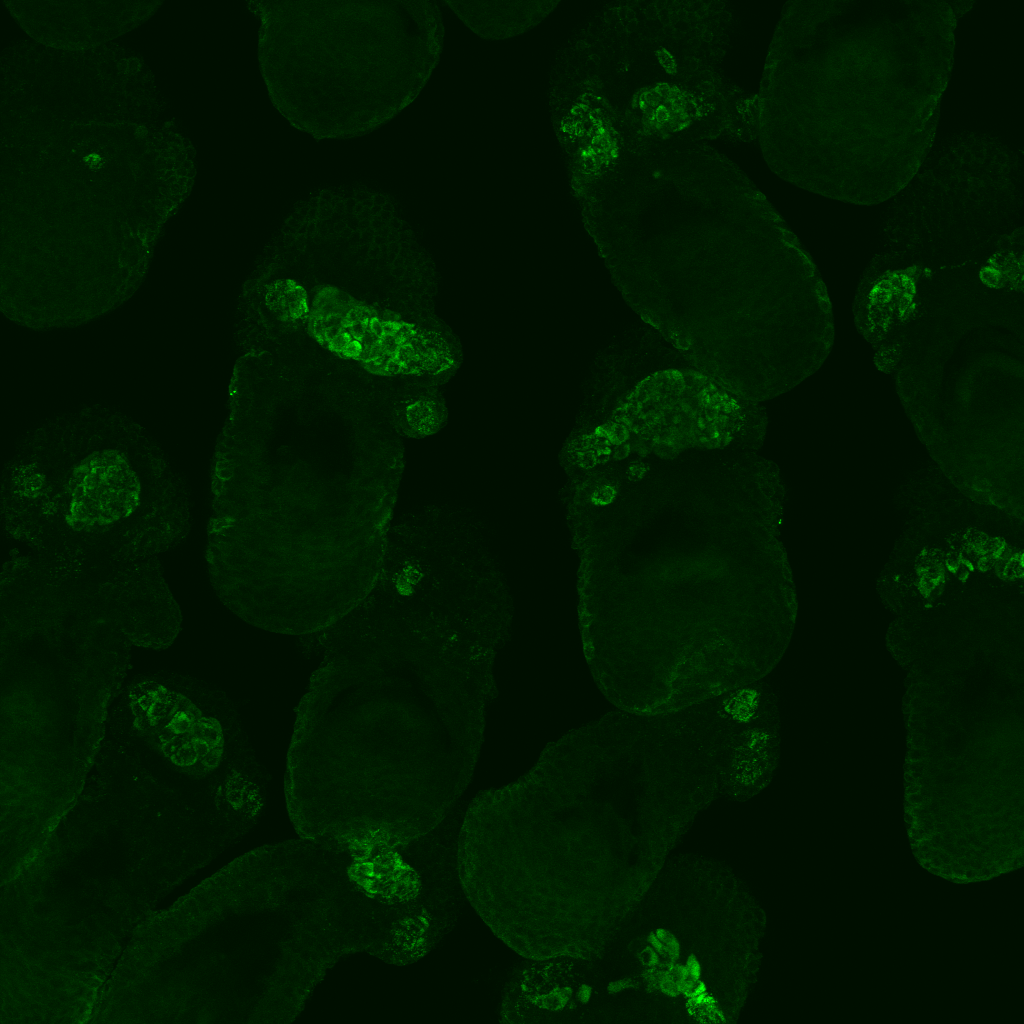

Supplement: Supplementary file 5 — Source data Fig. 4 [file 44318_2025_519_MOESM5_ESM.zip › Figure 4 Source Data/Fig. 4A SD/P6 Ift88EKO Adipophilin.tif]

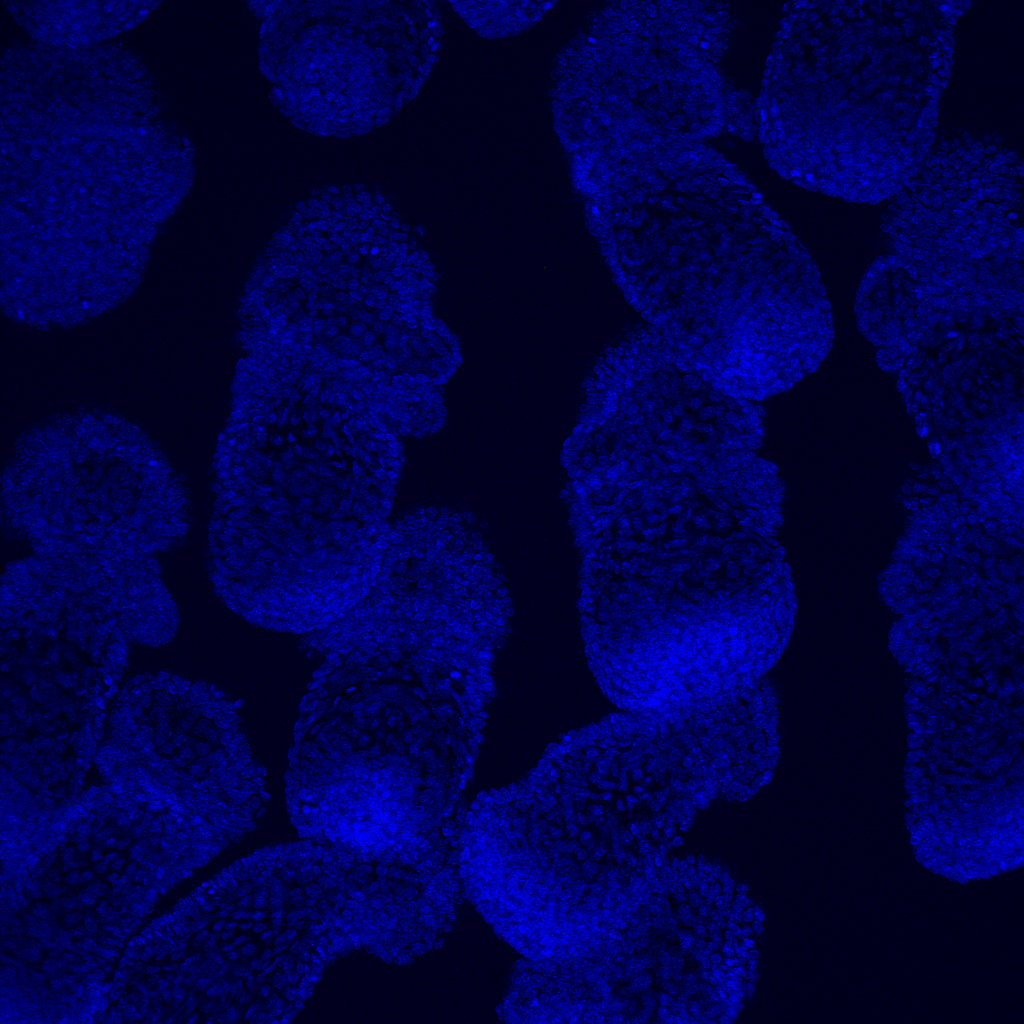

Supplement: Supplementary file 5 — Source data Fig. 4 [file 44318_2025_519_MOESM5_ESM.zip › Figure 4 Source Data/Fig. 4A SD/P6 Ift88EKO DAPI.tif]

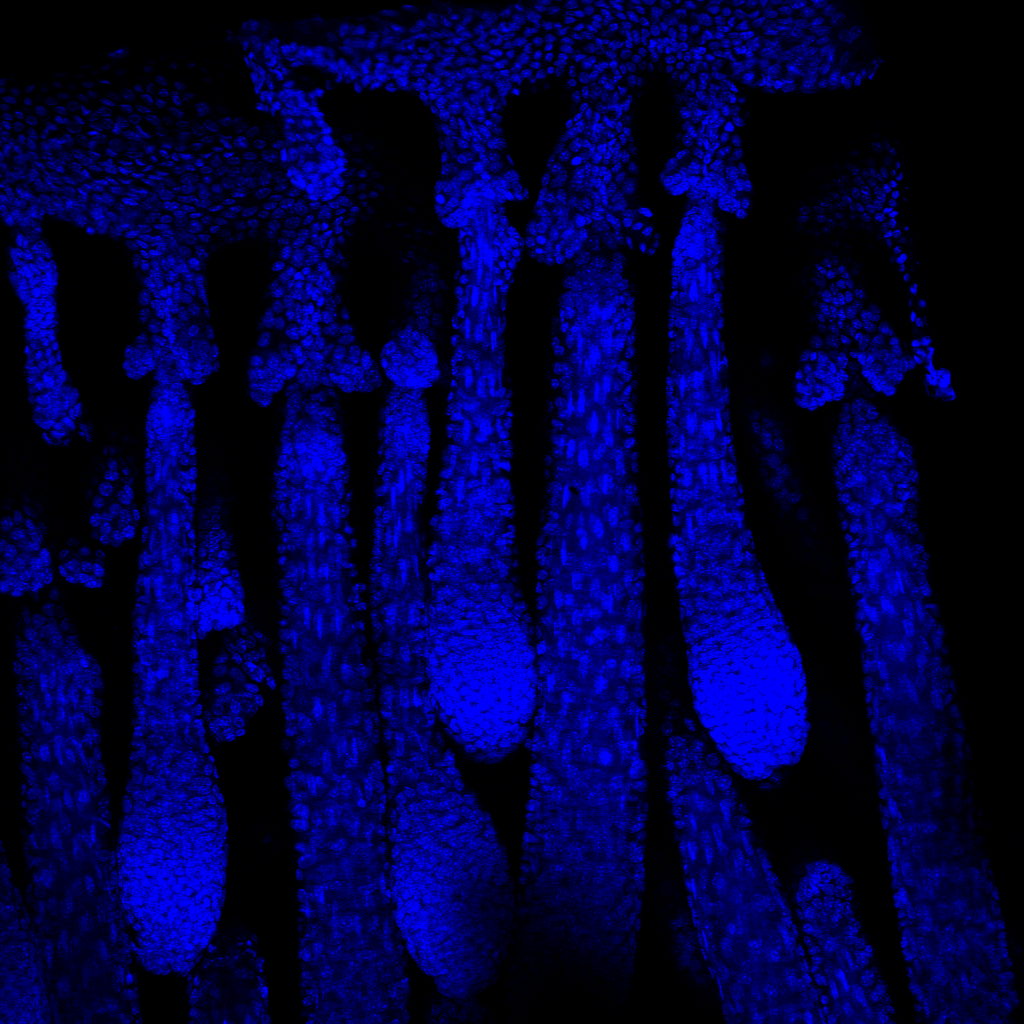

Supplement: Supplementary file 5 — Source data Fig. 4 [file 44318_2025_519_MOESM5_ESM.zip › Figure 4 Source Data/Fig. 4E SD/P6 Control DAPI.tif]

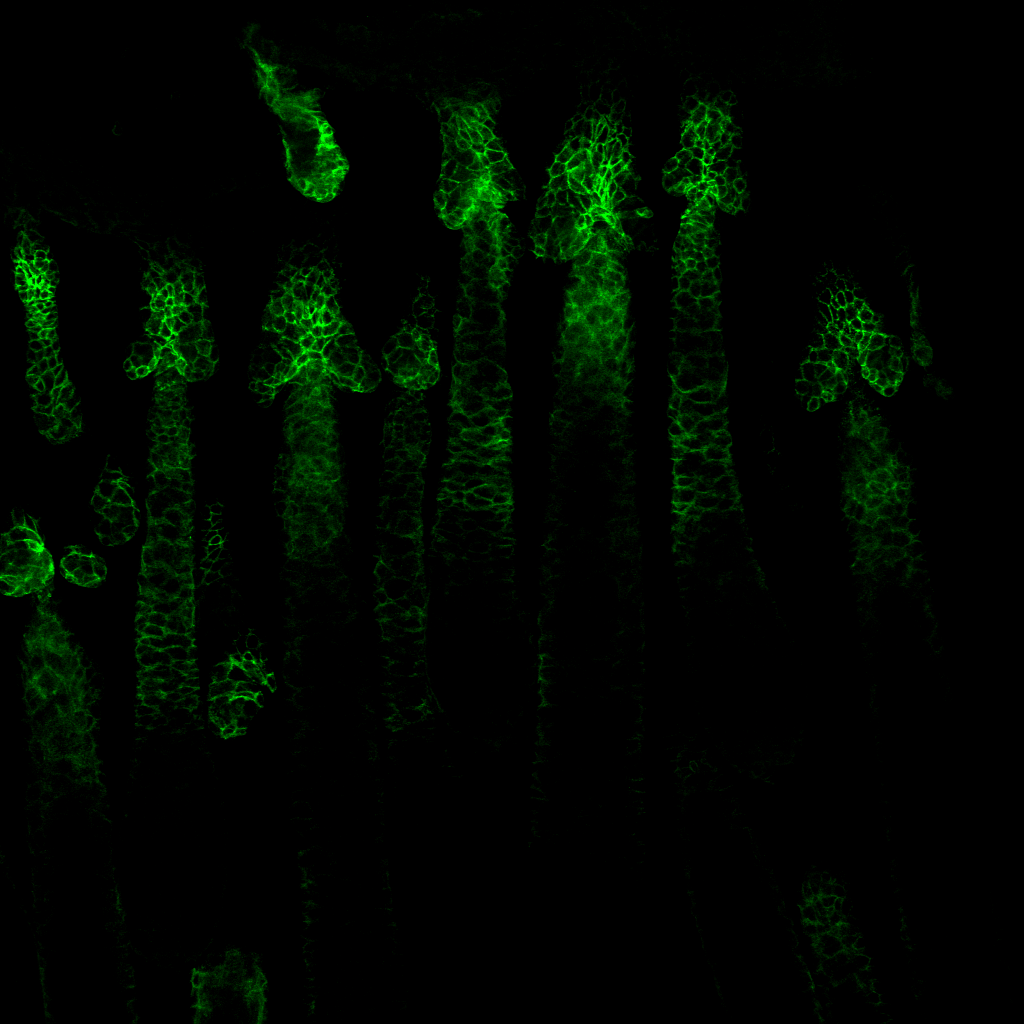

Supplement: Supplementary file 5 — Source data Fig. 4 [file 44318_2025_519_MOESM5_ESM.zip › Figure 4 Source Data/Fig. 4E SD/P6 Control Lrig1.tif]

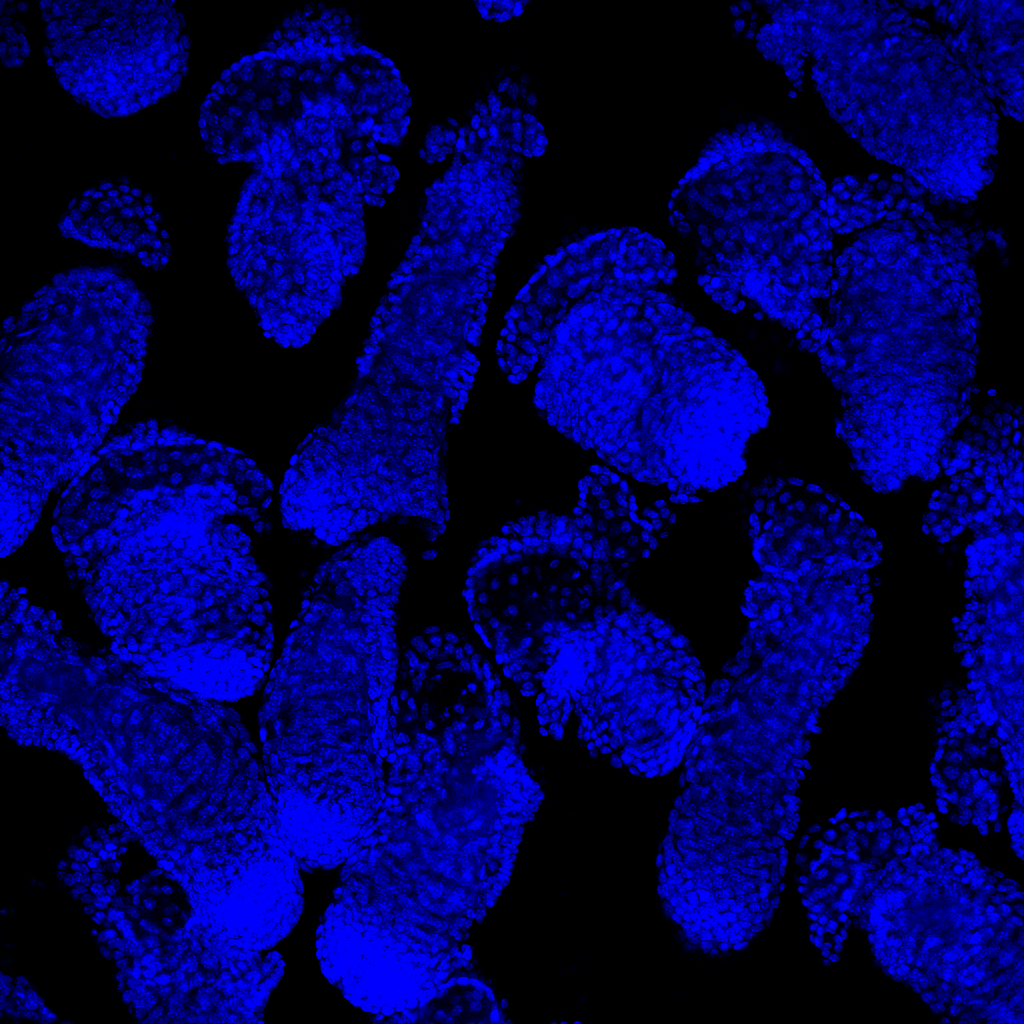

Supplement: Supplementary file 5 — Source data Fig. 4 [file 44318_2025_519_MOESM5_ESM.zip › Figure 4 Source Data/Fig. 4E SD/P6 Gli2EKO DAPI.tif]

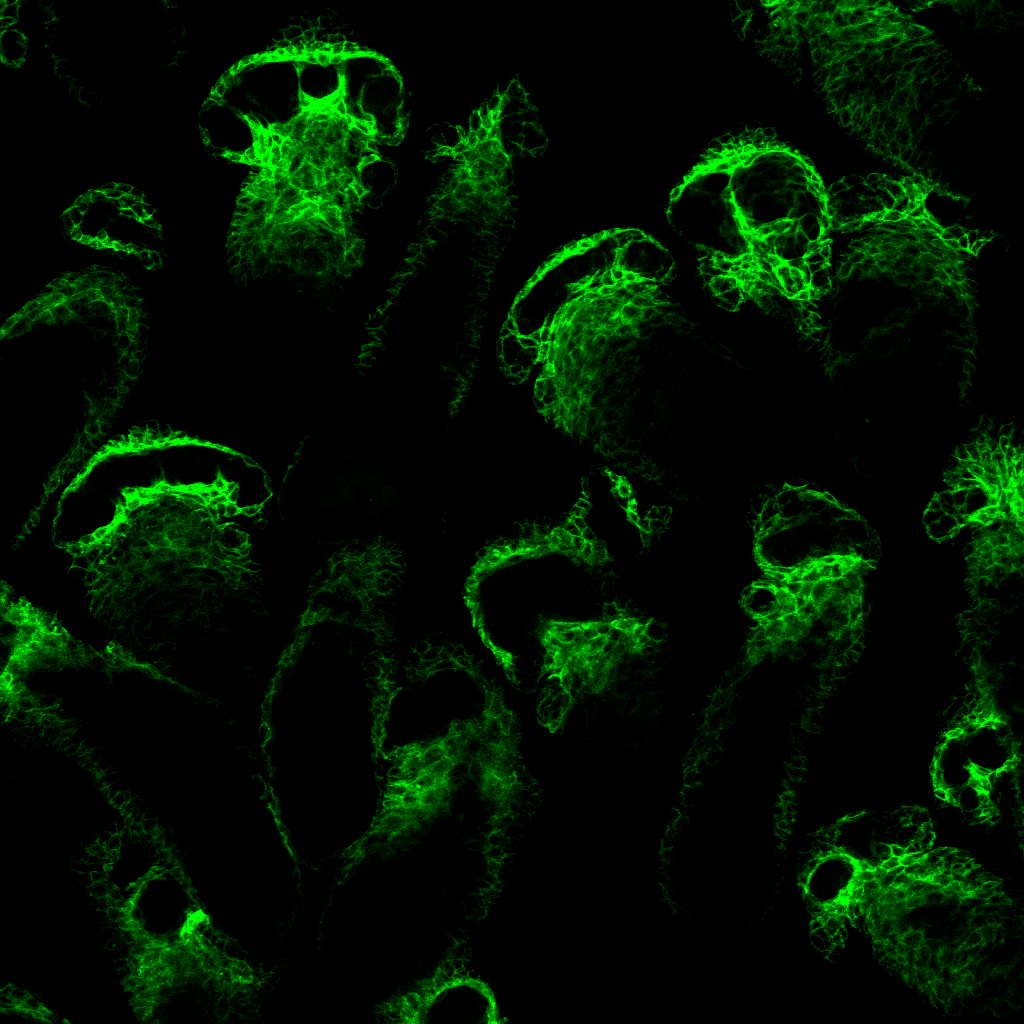

Supplement: Supplementary file 5 — Source data Fig. 4 [file 44318_2025_519_MOESM5_ESM.zip › Figure 4 Source Data/Fig. 4E SD/P6 Gli2EKO Lrig1.tif]

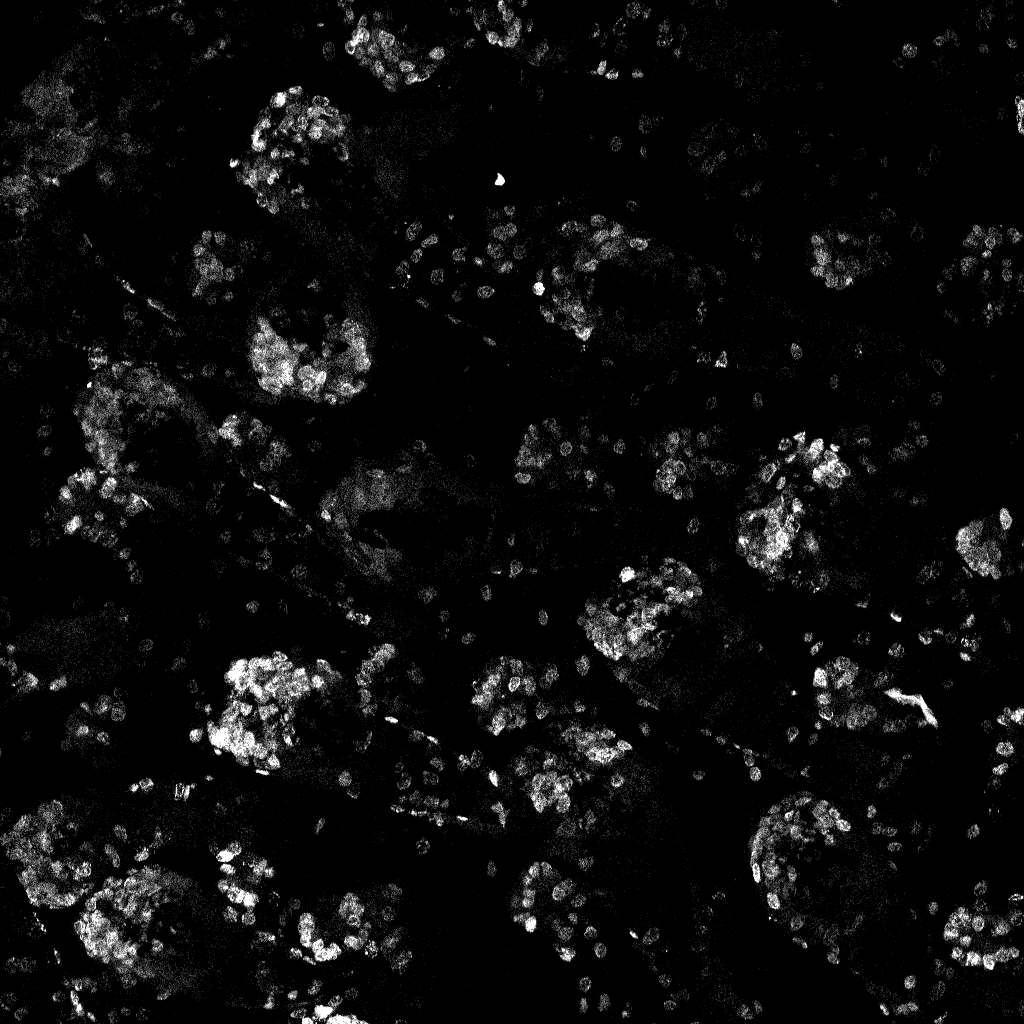

Supplement: Supplementary file 5 — Source data Fig. 4 [file 44318_2025_519_MOESM5_ESM.zip › Figure 4 Source Data/Fig. 4G SD/P0 Control BrdU.tif]

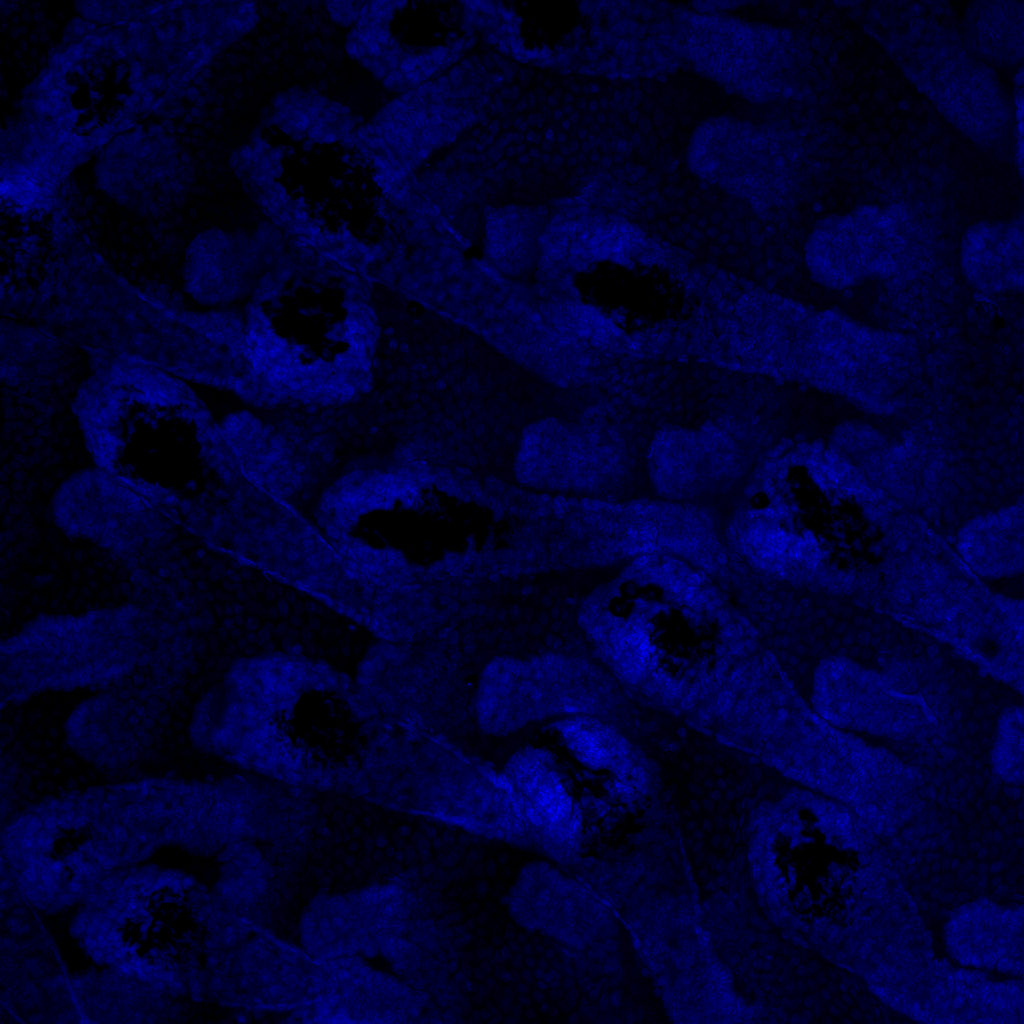

Supplement: Supplementary file 5 — Source data Fig. 4 [file 44318_2025_519_MOESM5_ESM.zip › Figure 4 Source Data/Fig. 4G SD/P0 Control DAPI.tif]

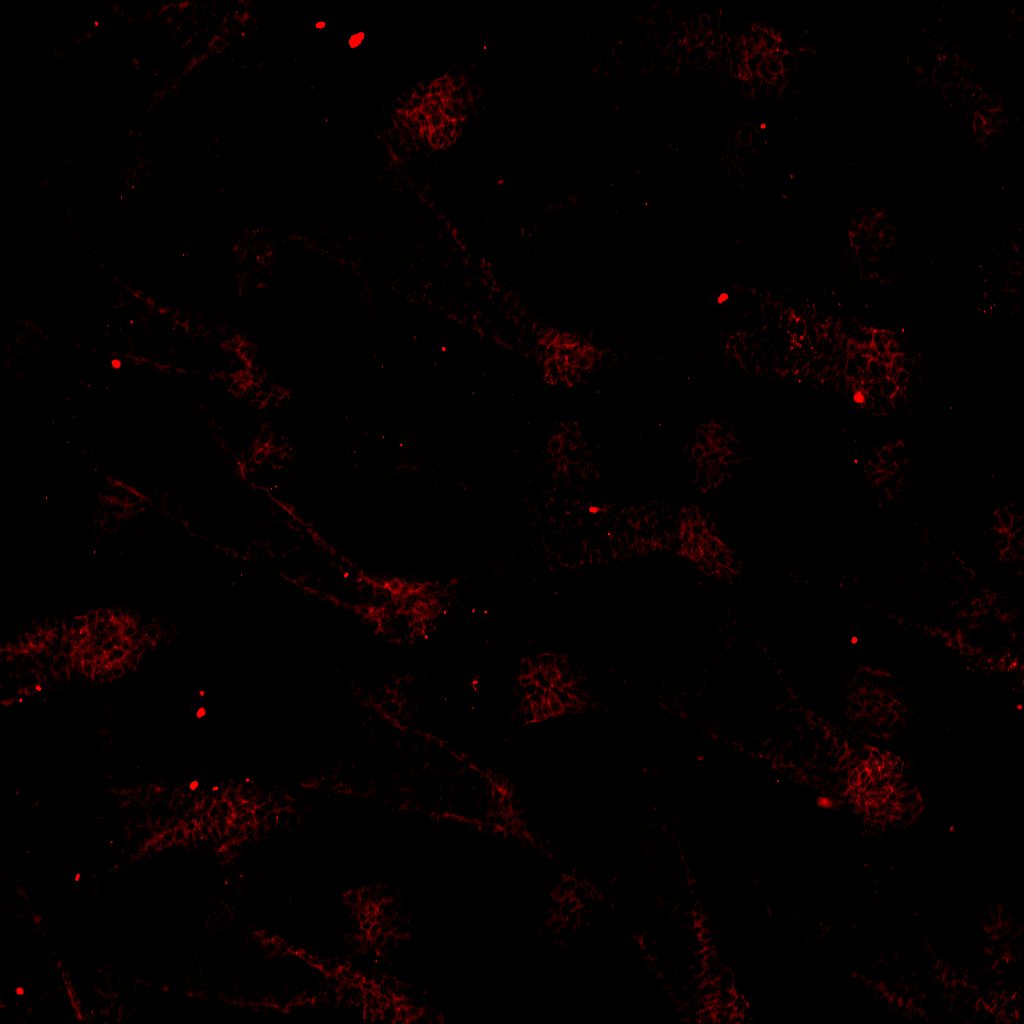

Supplement: Supplementary file 5 — Source data Fig. 4 [file 44318_2025_519_MOESM5_ESM.zip › Figure 4 Source Data/Fig. 4G SD/P0 Control Lrig1.tif]

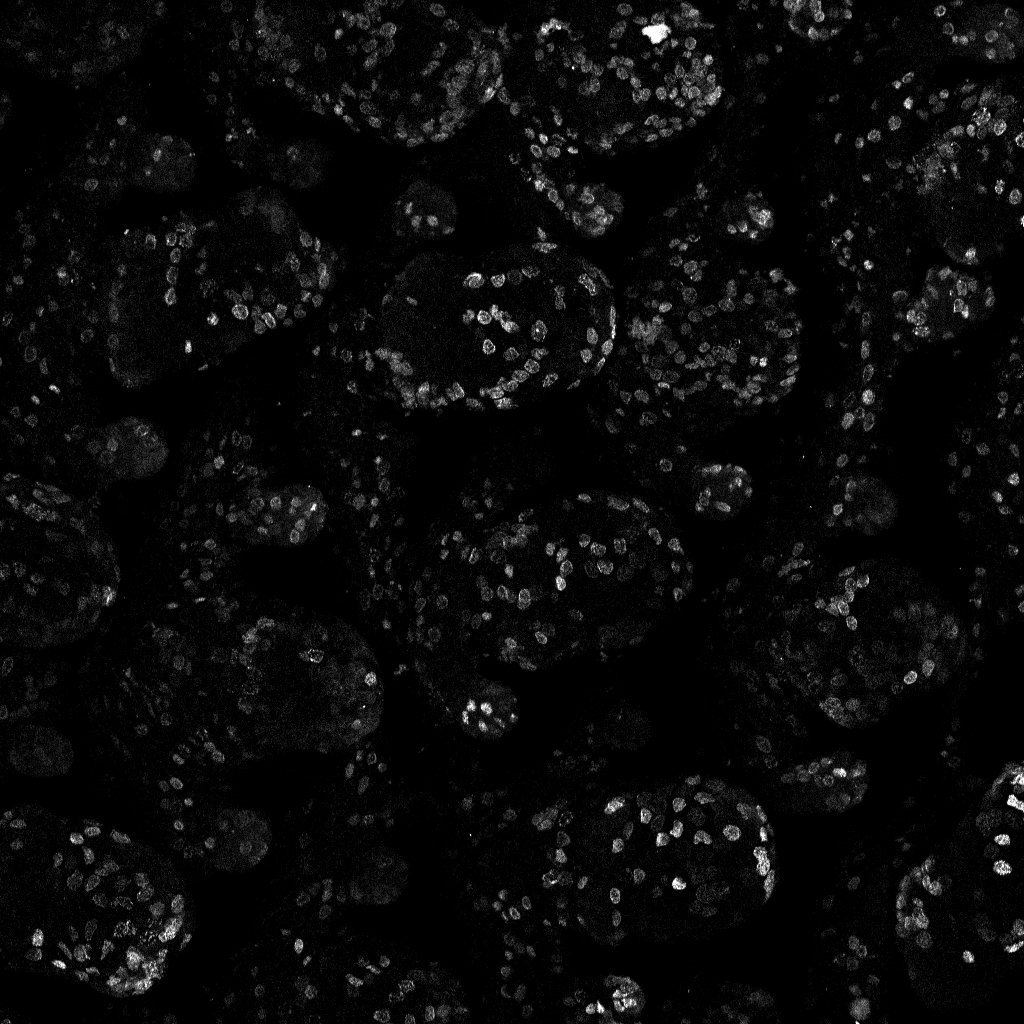

Supplement: Supplementary file 5 — Source data Fig. 4 [file 44318_2025_519_MOESM5_ESM.zip › Figure 4 Source Data/Fig. 4G SD/P0 Gli2EKO BrdU.tif]

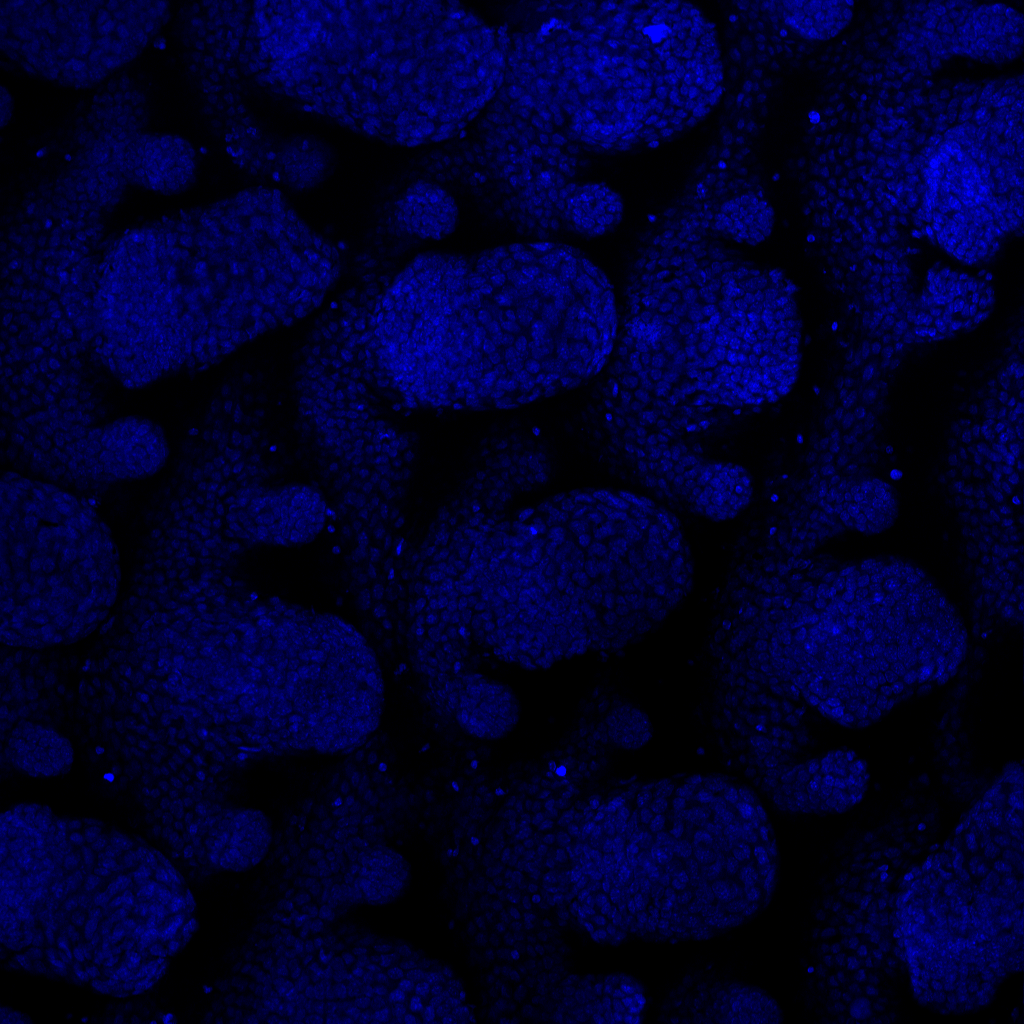

Supplement: Supplementary file 5 — Source data Fig. 4 [file 44318_2025_519_MOESM5_ESM.zip › Figure 4 Source Data/Fig. 4G SD/P0 Gli2EKO DAPI.tif]

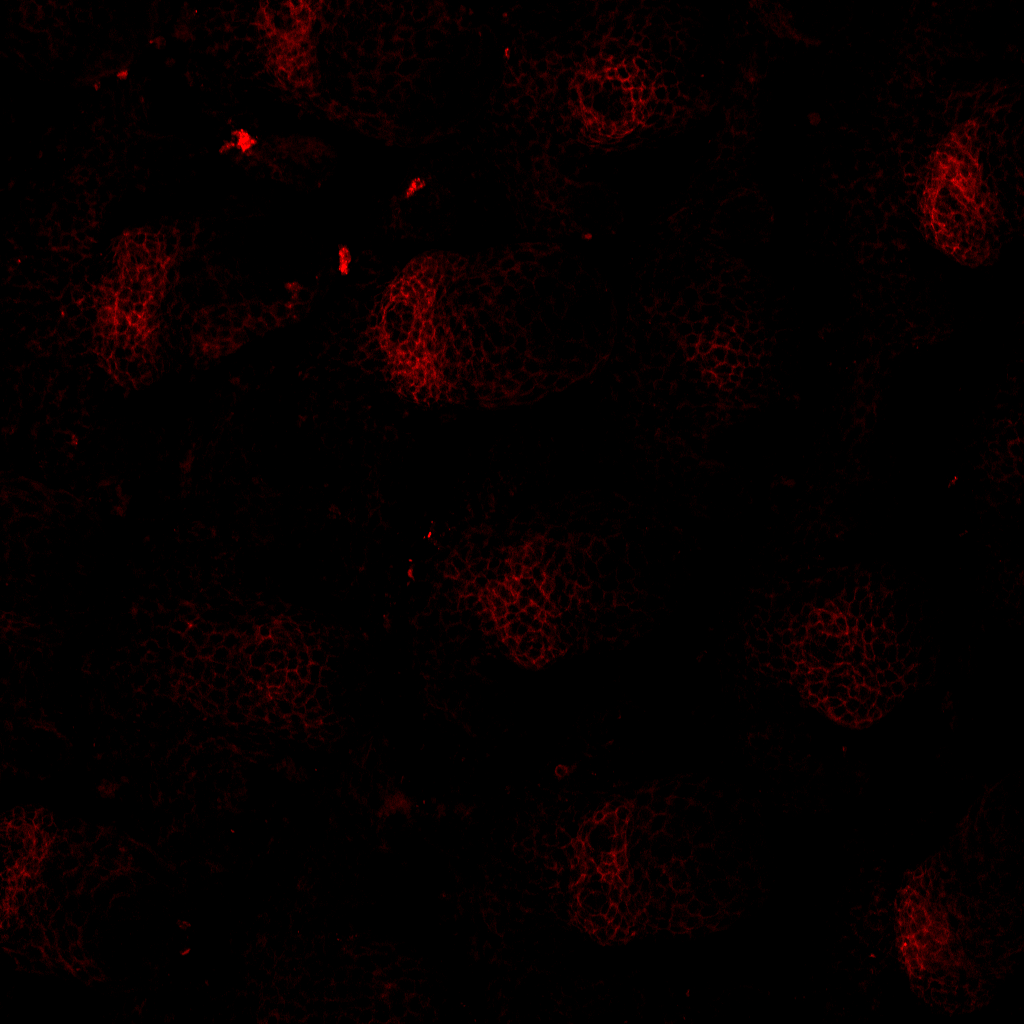

Supplement: Supplementary file 5 — Source data Fig. 4 [file 44318_2025_519_MOESM5_ESM.zip › Figure 4 Source Data/Fig. 4G SD/P0 Gli2EKO Lrig1.tif]

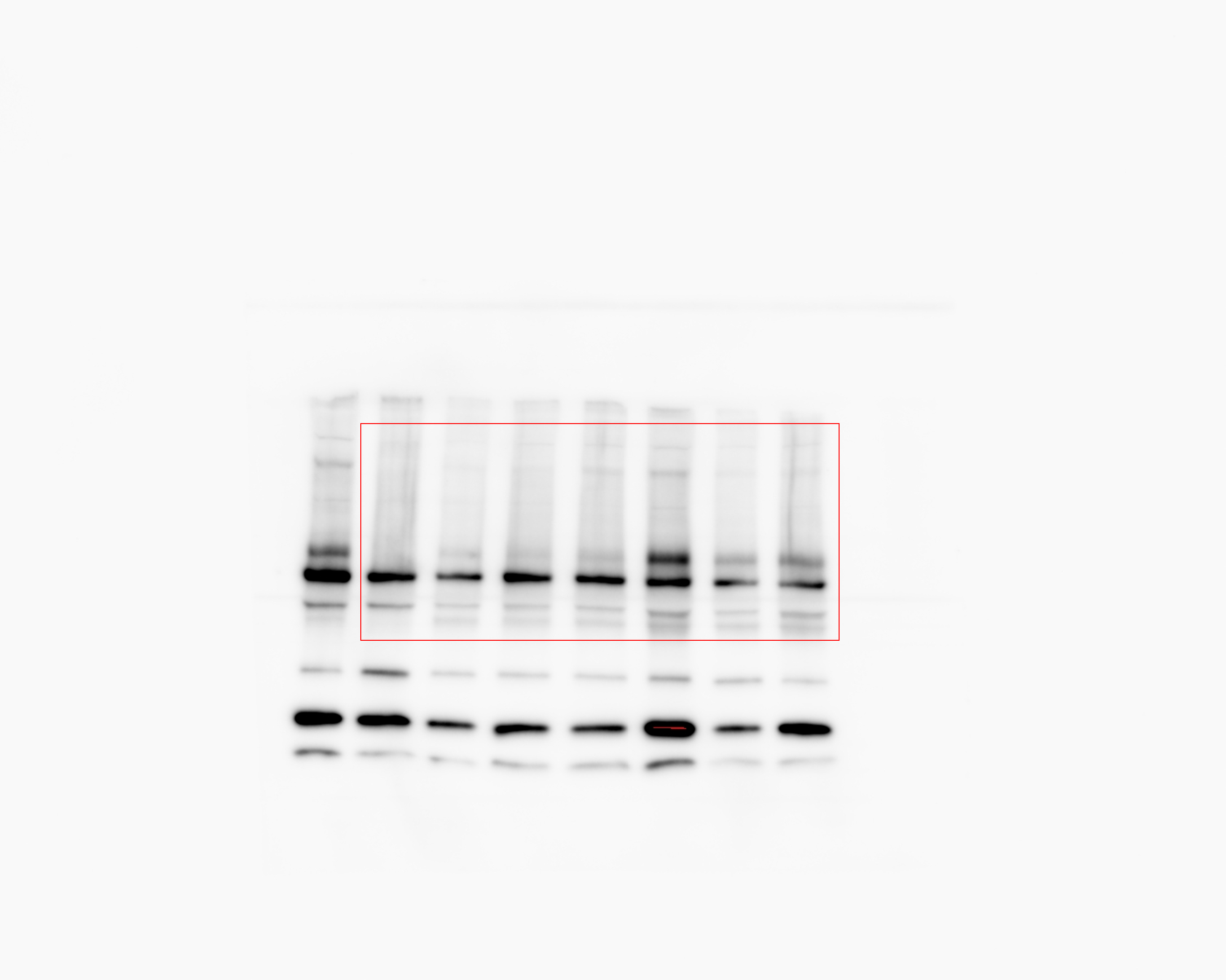

Supplement: Supplementary file 7 — Source data Fig. 6 [file 44318_2025_519_MOESM7_ESM.zip › Figure 6 Source Data/Fig. 6A SD/Western Blot Gli3+Ladder/Western Blot Gli3 ROI.png]

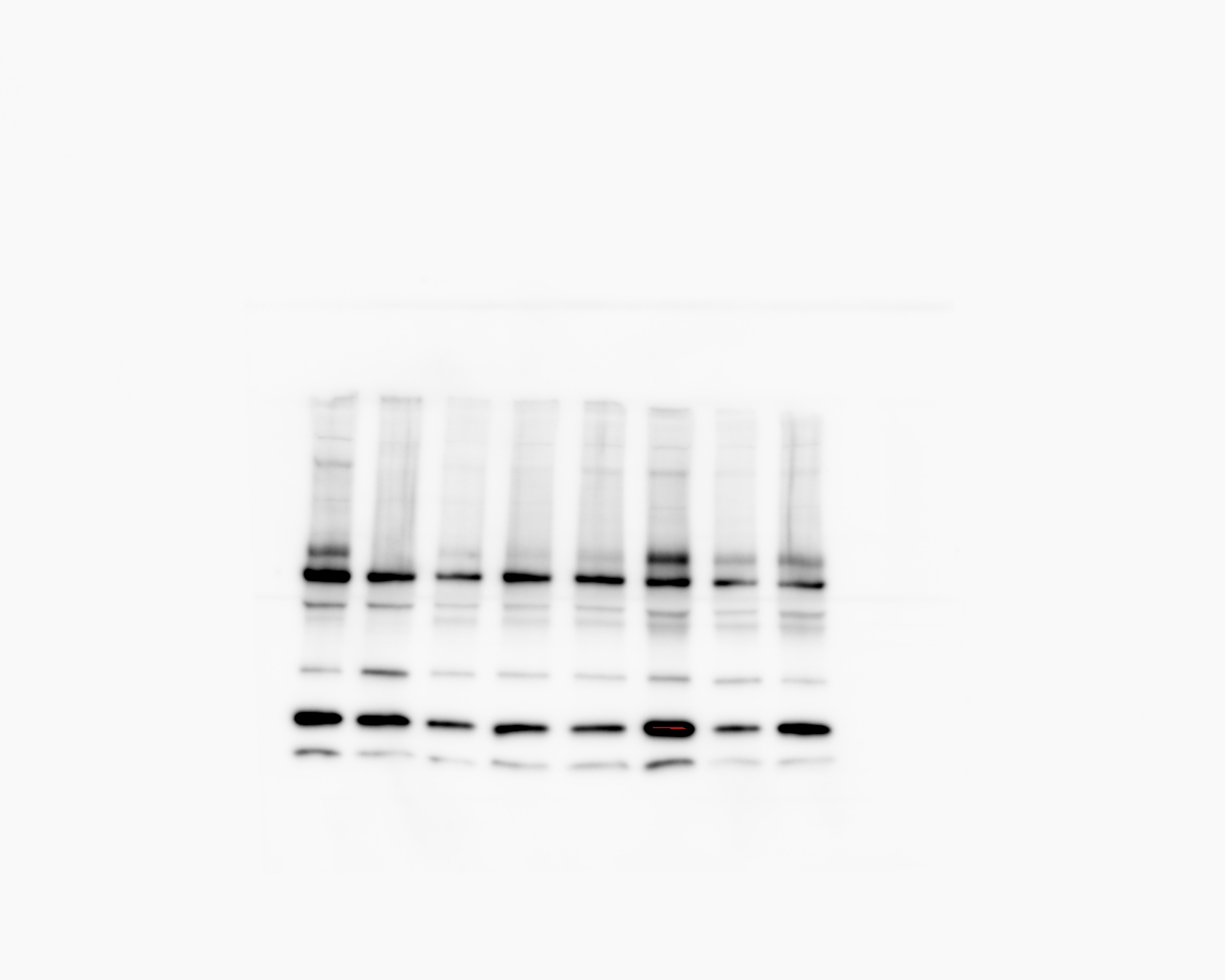

Supplement: Supplementary file 7 — Source data Fig. 6 [file 44318_2025_519_MOESM7_ESM.zip › Figure 6 Source Data/Fig. 6A SD/Western Blot Gli3+Ladder/Western Blot Gli3.jpg]

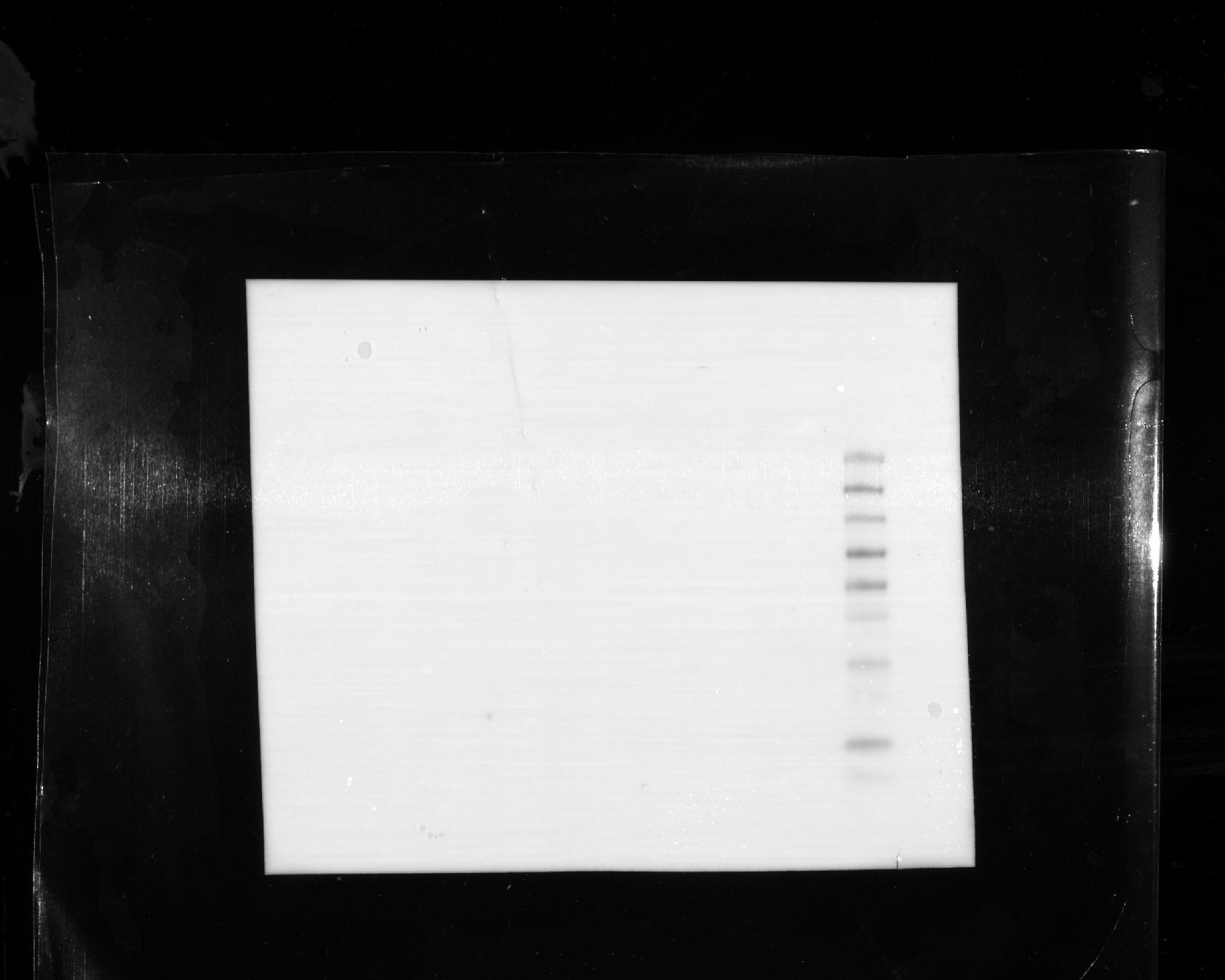

Supplement: Supplementary file 7 — Source data Fig. 6 [file 44318_2025_519_MOESM7_ESM.zip › Figure 6 Source Data/Fig. 6A SD/Western Blot Gli3+Ladder/Western Blot Ladder.tif]

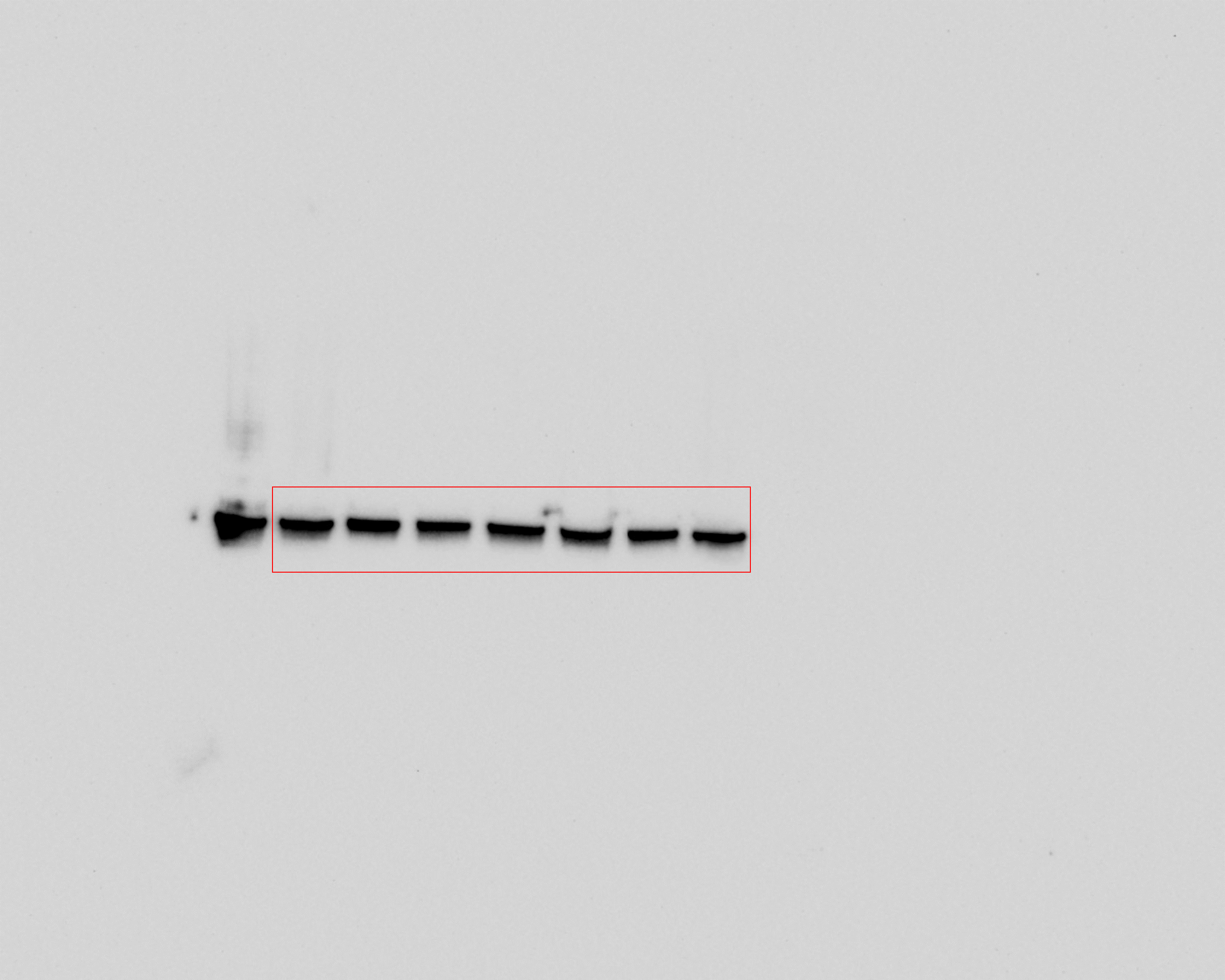

Supplement: Supplementary file 7 — Source data Fig. 6 [file 44318_2025_519_MOESM7_ESM.zip › Figure 6 Source Data/Fig. 6A SD/Western Blot K14+Ladder/Western Blot K14 ROI.png]

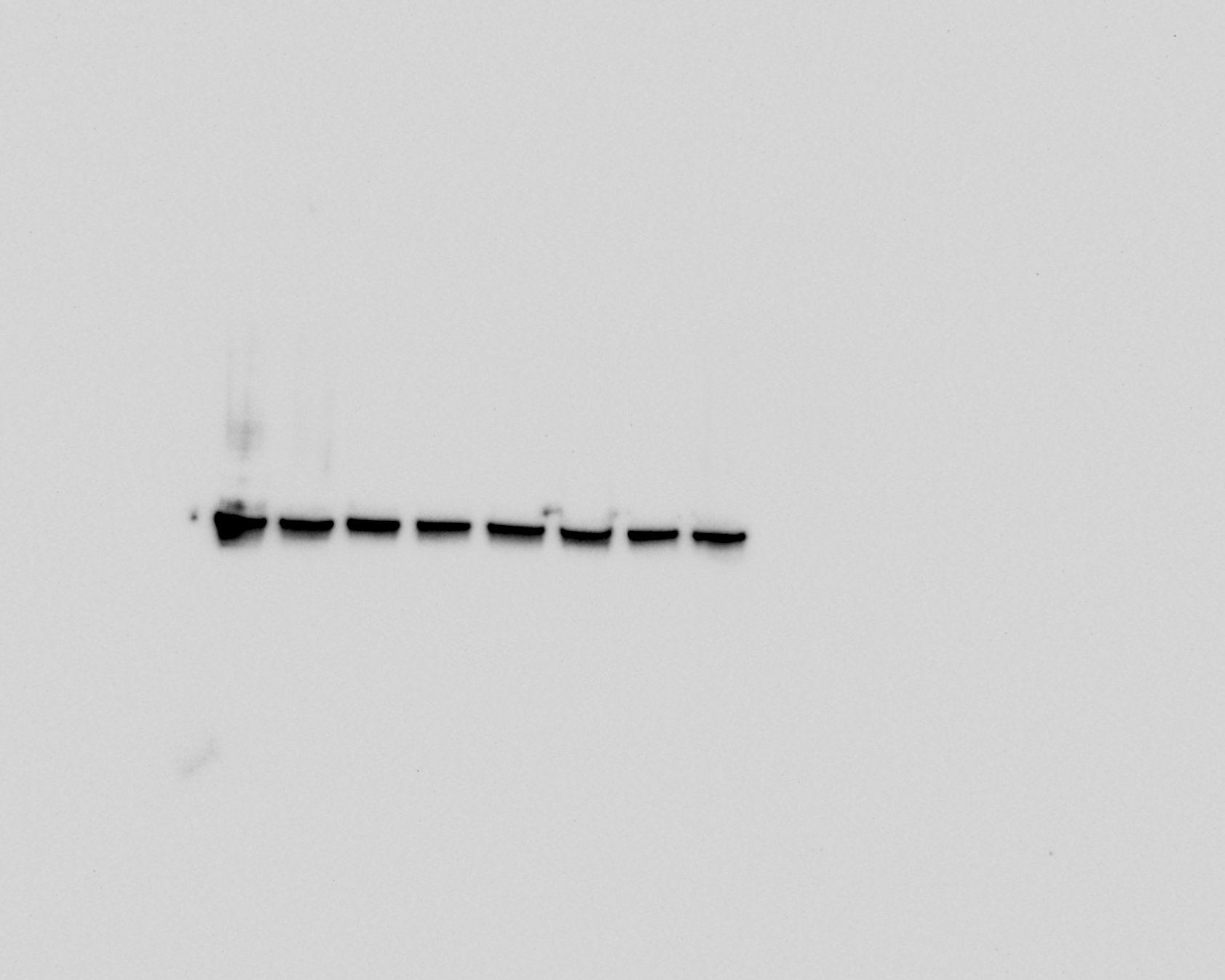

Supplement: Supplementary file 7 — Source data Fig. 6 [file 44318_2025_519_MOESM7_ESM.zip › Figure 6 Source Data/Fig. 6A SD/Western Blot K14+Ladder/Western Blot K14.tif]

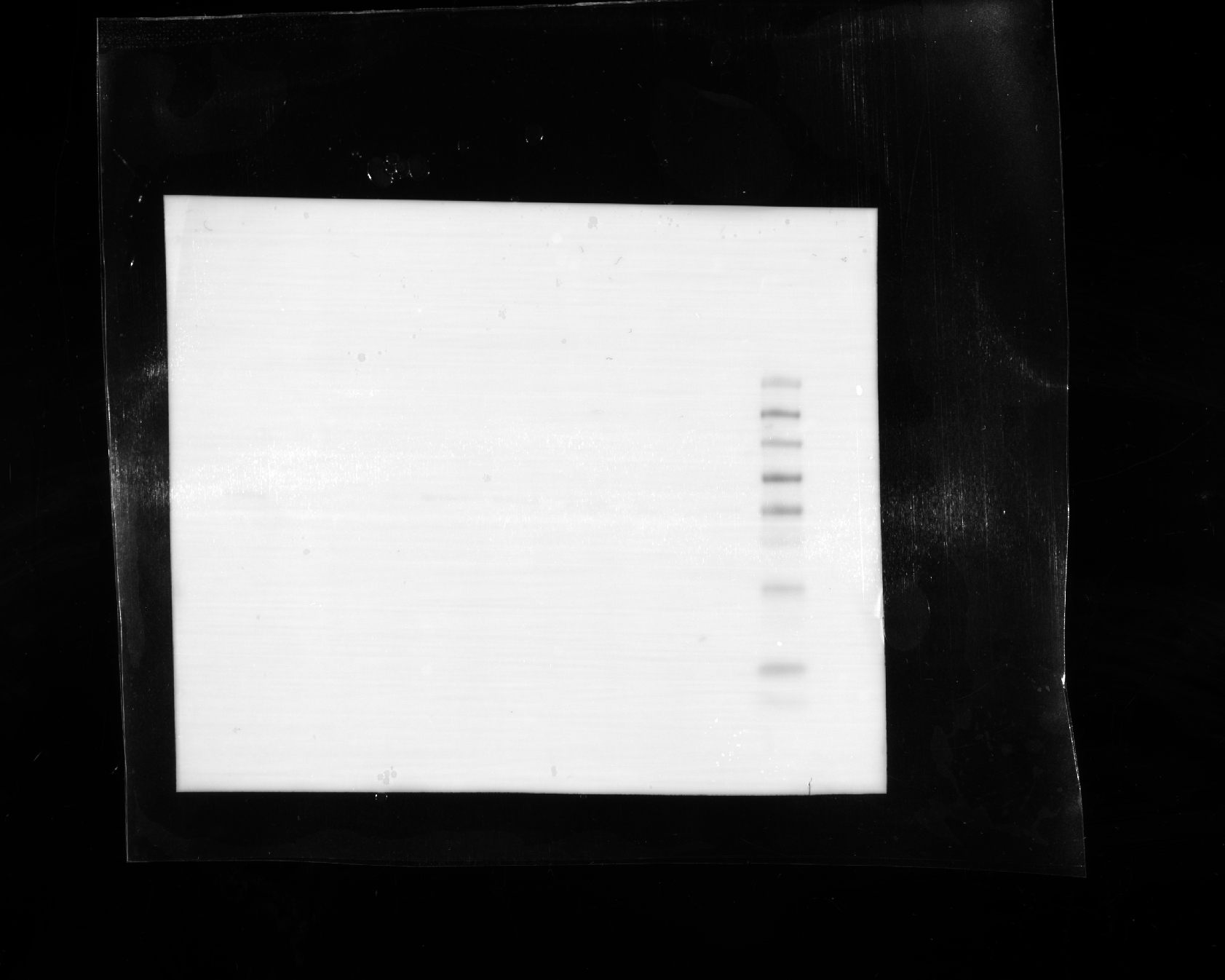

Supplement: Supplementary file 7 — Source data Fig. 6 [file 44318_2025_519_MOESM7_ESM.zip › Figure 6 Source Data/Fig. 6A SD/Western Blot K14+Ladder/Western Blot Ladder.jpg]
