# Supplementary material for: Specific and redundant roles for Gli2 and Gli3 in establishing cell fate during murine hair follicle development
Source: EMBO J. 2025 Aug 26;44(19):5290–314. doi: 10.1038/s44318-025-00519-9 (PMC12488920; doi:10.1038/s44318-025-00519-9)
Supplement: Supplementary file 6 — Source data Fig. 5 [file 44318_2025_519_MOESM6_ESM.zip › Figure 5 Source Data/P2 Control and Gli2EKO back skin whole mounts.pptx]

## Slide 1
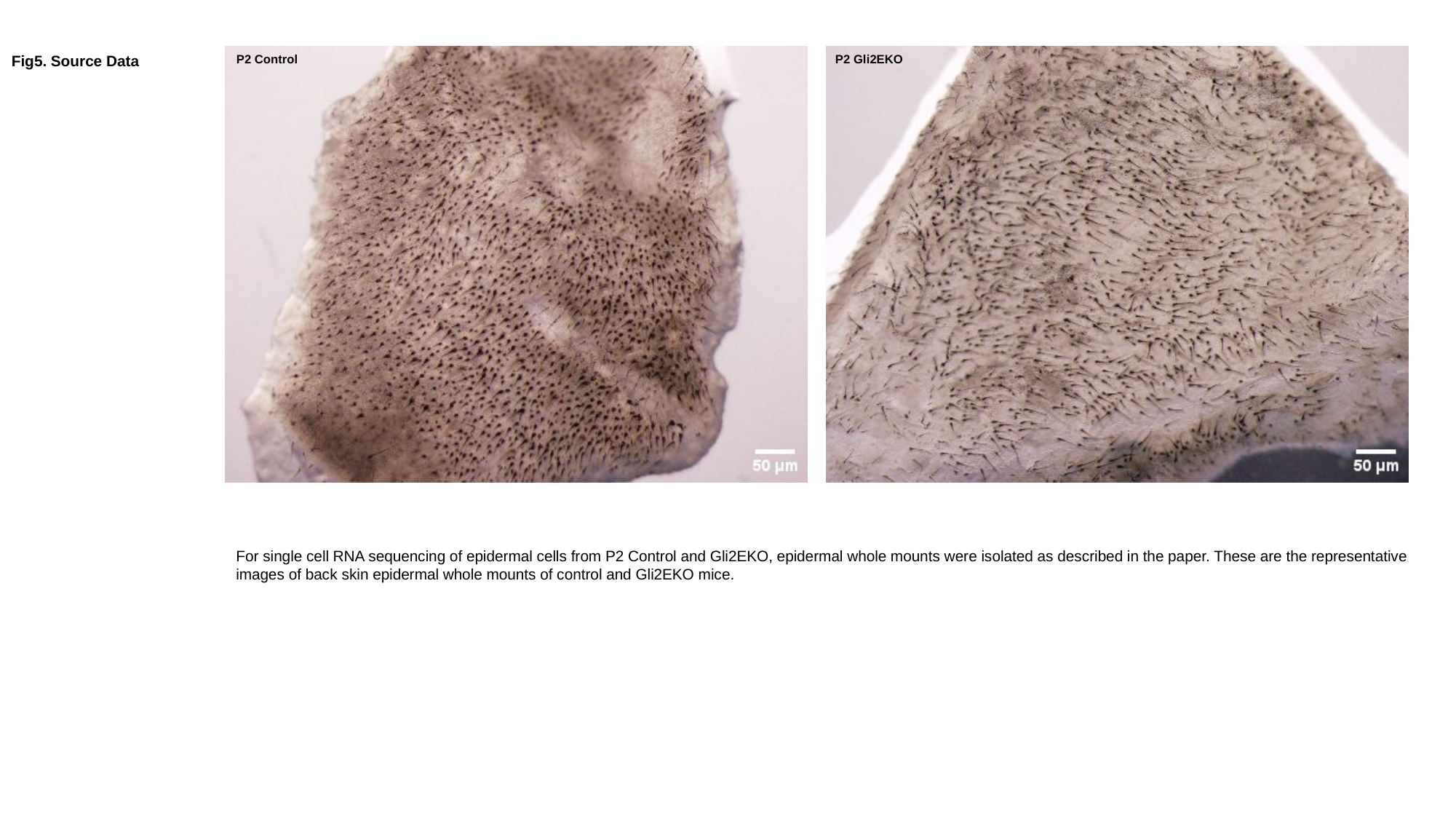

Fig5. Source Data
P2 Control
P2 Gli2EKO
For single cell RNA sequencing of epidermal cells from P2 Control and Gli2EKO, epidermal whole mounts were isolated as described in the paper. These are the representative images of back skin epidermal whole mounts of control and Gli2EKO mice.
