# Supplementary material for: Specific and redundant roles for Gli2 and Gli3 in establishing cell fate during murine hair follicle development
Source: EMBO J. 2025 Aug 26;44(19):5290–314. doi: 10.1038/s44318-025-00519-9 (PMC12488920; doi:10.1038/s44318-025-00519-9)
Supplement: Supplementary file 8 — Expanded View Figures [file 44318_2025_519_MOESM8_ESM.pdf]

Expanded View Figures

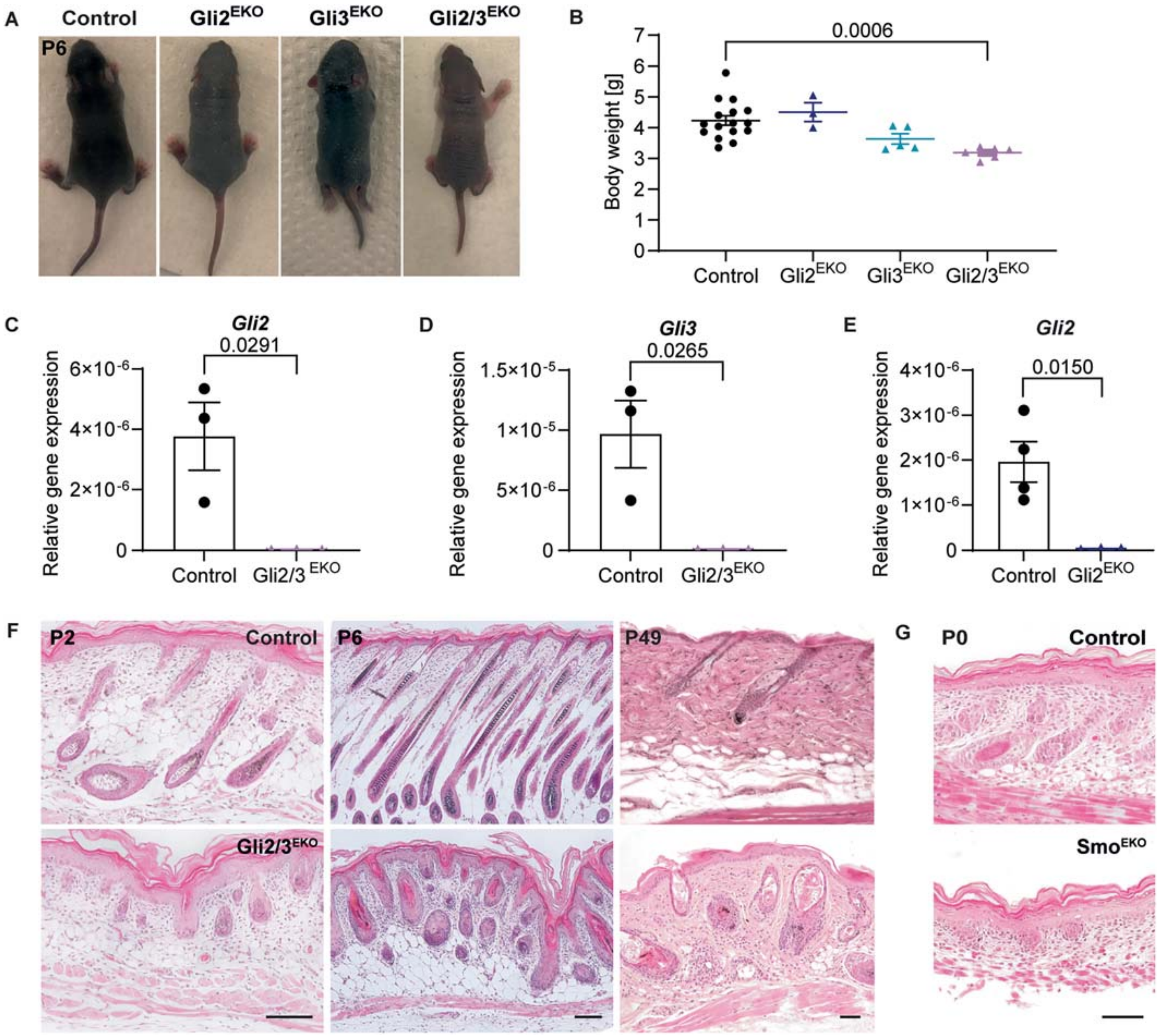

Figure EV1. Characterisation of Gli^{EKO} and Smo^{EKO} mouse models.

(**A**, **B**) Representative images (**A**) and body weight (**B**) of P6 Gli2^{EKO}, Gli3^{EKO}, Gli2/3^{EKO} and control mice. ($n = 3$-16mice/genotype). (**C**, **D**) qRT-PCR analysis for Gli2 (**C**) and Gli3 (**D**) mRNA expression in tail epidermis from P6 Gli2/3^{EKO} and control littermates. Each datapoint represents one animal. ($n = 3$ mice/genotype). (**E**) qRT-PCR analysis for Gli2 mRNA expression in back skin epidermis from P3 Gli2^{EKO} and control littermates. Each datapoint represents one animal. ($n = 3$–4 mice/genotype). (**F**) Representative H&E staining of back skin sections from P2, P6 and P49 Gli2/3^{EKO} and control littermates ($n = 3$ mice/genotype). (**G**) Representative H&E staining of back skin sections from P0 Smo^{EKO} and control littermates. Each datapoint represents one animal. ($n = 3$ mice/genotype). Scale bars 50 µm (**F**, **G**). Data were presented as mean ± SEM. *P* value was calculated using an unpaired Student's *t*-test.

 © The Author(s)

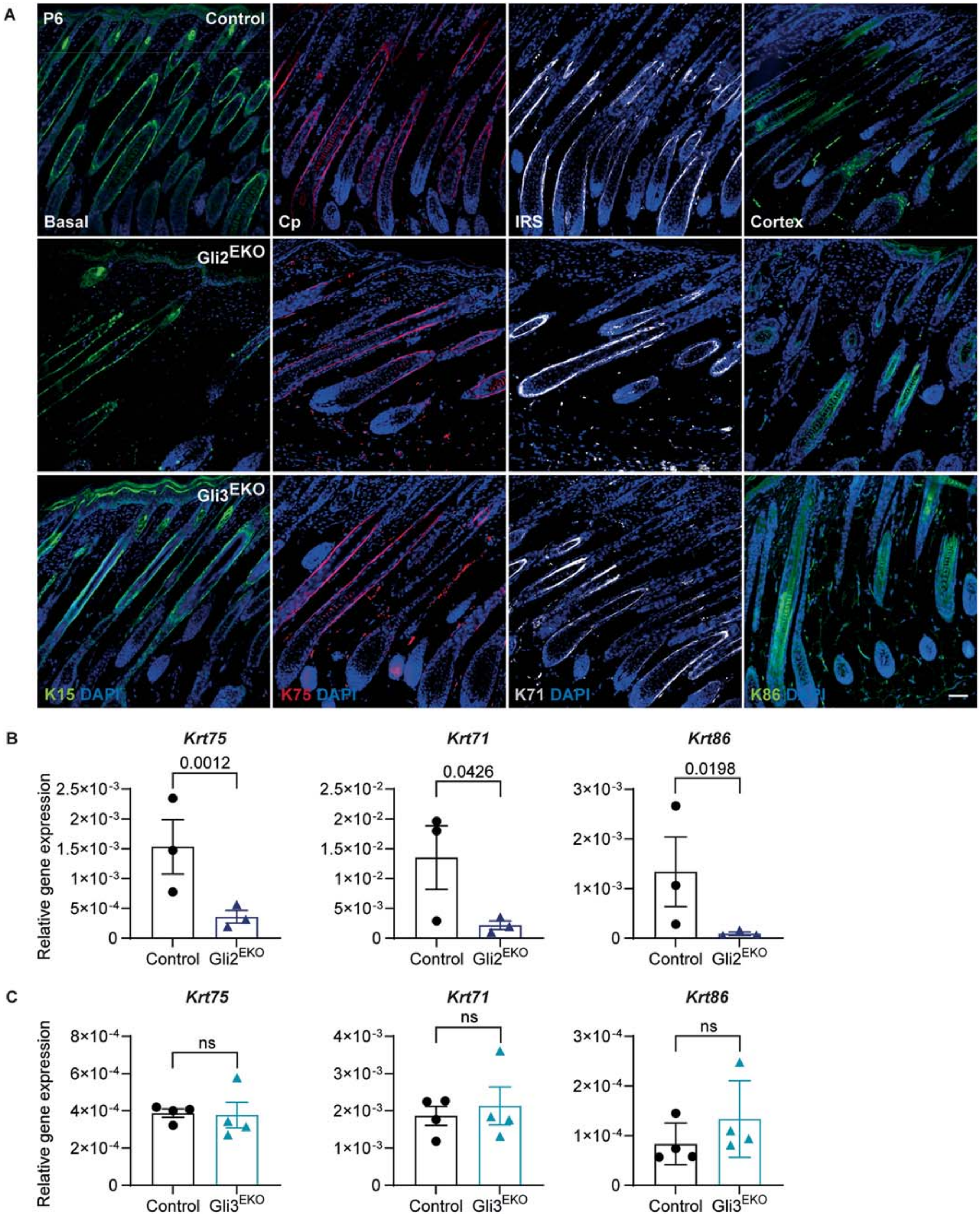
A
P6
Control
Basal
Cp
IRS
Cortex
Gli2EKO
Gli3EKO
K15 DAPI
K75 DAPI
K71 DAPI
K86 DAPI
B
Krt75
0.0012
Krt71
0.0426
Krt86
0.0198
Relative gene expression
Control
Gli2EKO
C
Krt75
Krt71
Krt86
ns
Relative gene expression
Control
Gli3EKO

Figure EV2. Analysis of hair lineage differentiation in Gli2^{EKO} and Gli3^{EKO} mice.

(**A**) Immunofluorescence staining for K15 (green), K75 (red), K71 (grey), K86 (green) and DAPI (blue, nuclei) of back skin sections from P6 Gli2^{EKO}, Gli3^{EKO} and control mice. ($n = 3$ mice/genotype) (redisplay of data for control mice from Fig. 2A). (**B**, **C**) qRT-PCR analysis of Krt75, Krt71 and Krt86 mRNA expression in back skin of P6 Gli2^{EKO} (**B**) and Gli3^{EKO} (**C**) and control littermates. ($n = 3$–4 mice/genotype). Scale bar 50 µm. Data were represented as mean ± SEM. *P* value was calculated using paired (**B**) and unpaired Student's *t*-test (**C**).

© The Author(s)

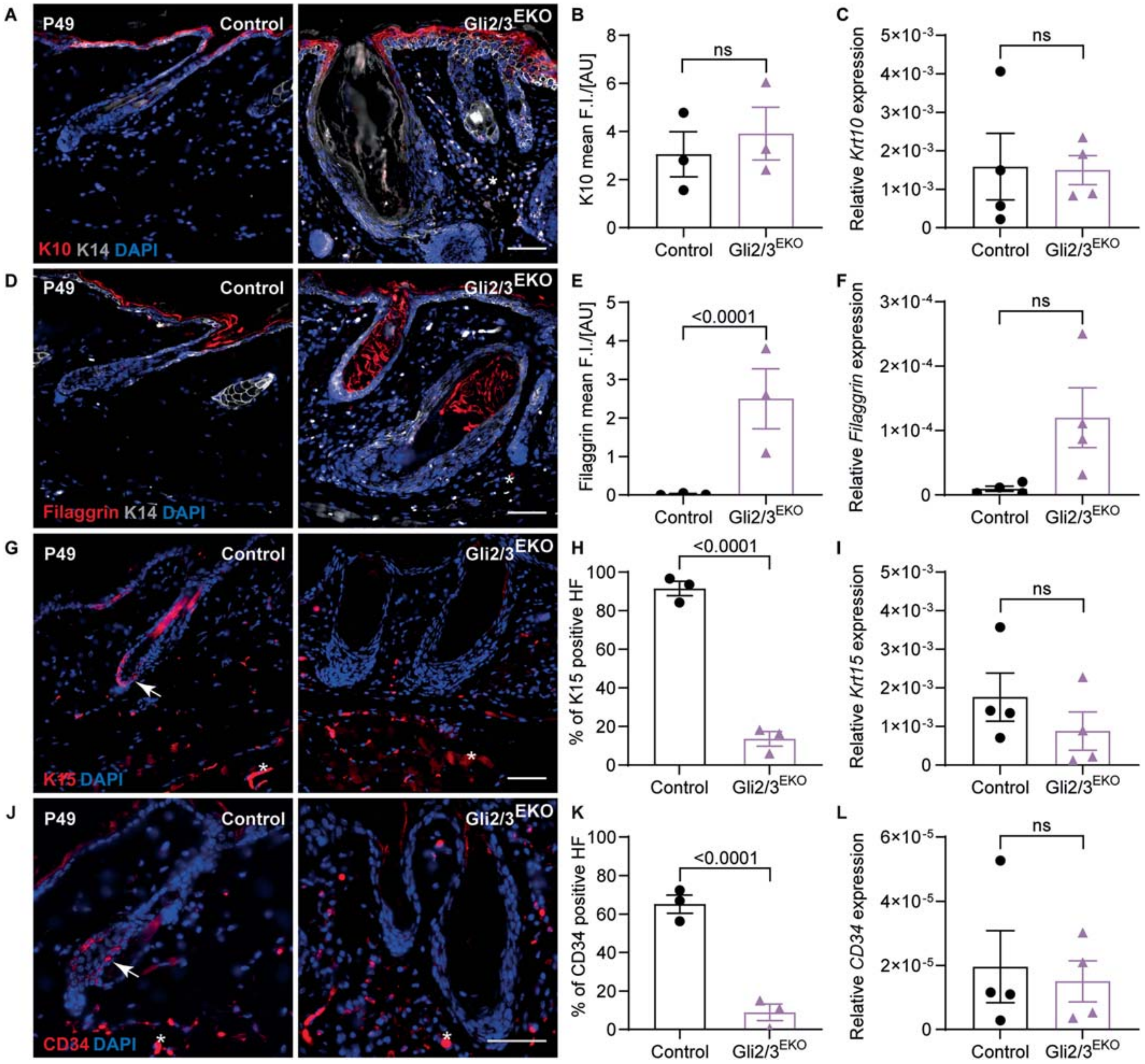

Figure EV3. Defective hair differentiation in Gli2/3^{EKO} mice.

(**A**, **B**) Immunofluorescence staining for K10 (red), K14(grey) and DAPI (blue, nuclei) (**A**) and quantification of K10 (**B**) in back skin sections from P49 Gli2/3^{EKO} and control littermates ($n = 3$ mice/genotype). K10 fluorescence intensity (FI) per arbitrary unit (AU). (**C**) qRT-PCR analysis of Krt10 mRNA expression in back skin from P49 Gli2/3^{EKO} and control littermates. The results shown are from two technical replicates ($n = 2$ mice/genotype). (**D**, **E**) Immunofluorescence staining for Filaggrin (red), K14 (grey) and DAPI (blue, nuclei) (**D**) and quantification of filaggrin (**E**) in back skin sections from P49 Gli2/3^{EKO} and control littermates ($n = 3$ mice/genotype). Filaggrin fluorescence intensity (FI) per arbitrary unit (AU) (p value = 8.42e-06). (**F**) qRT-PCR analysis of filaggrin mRNA expression in back skin from P49 Gli2/3^{EKO} and control littermates. The results shown are from two technical replicates ($n = 2$ mice/genotype). (**G**, **H**) Immunofluorescence staining for K15 (red, arrow) and DAPI (blue, nuclei) (**G**) and quantification of K15 (**H**) in back skin sections from P49 Gli2/3^{EKO} and control littermates. 50-147 HFs per animal were counted. ($n = 3$ mice/genotype) (p value = 5.4e-17). (**I**) qRT-PCR analysis of K15 mRNA expression in back skin from P49 Gli2/3^{EKO} and control littermates. The results shown are from two technical replicates ($n = 2$ mice/genotype). (**J**, **K**) Immunofluorescence staining for CD34 (red, arrow) and DAPI (blue, nuclei) (**J**) and quantification of CD34 (**K**) in back skin sections from P49 Gli2/3^{EKO} and control littermates. 106–175 HFs per animal were counted. ($n = 3$ mice/genotype) (p value = 8.17e-24). (**L**) qRT-PCR analysis of CD34 mRNA expression in back skin form P49 Gli2/3^{EKO} and control littermates. The results shown are from two technical replicates ($n = 2$ mice/genotype). Scale bars 50 µm (**A**, **D**, **G**, **J**). An asterisk marks an unspecified signal (**G**, **J**). Data were represented as mean ± SEM. *P* value was calculated using an unpaired Student's *t*-test.

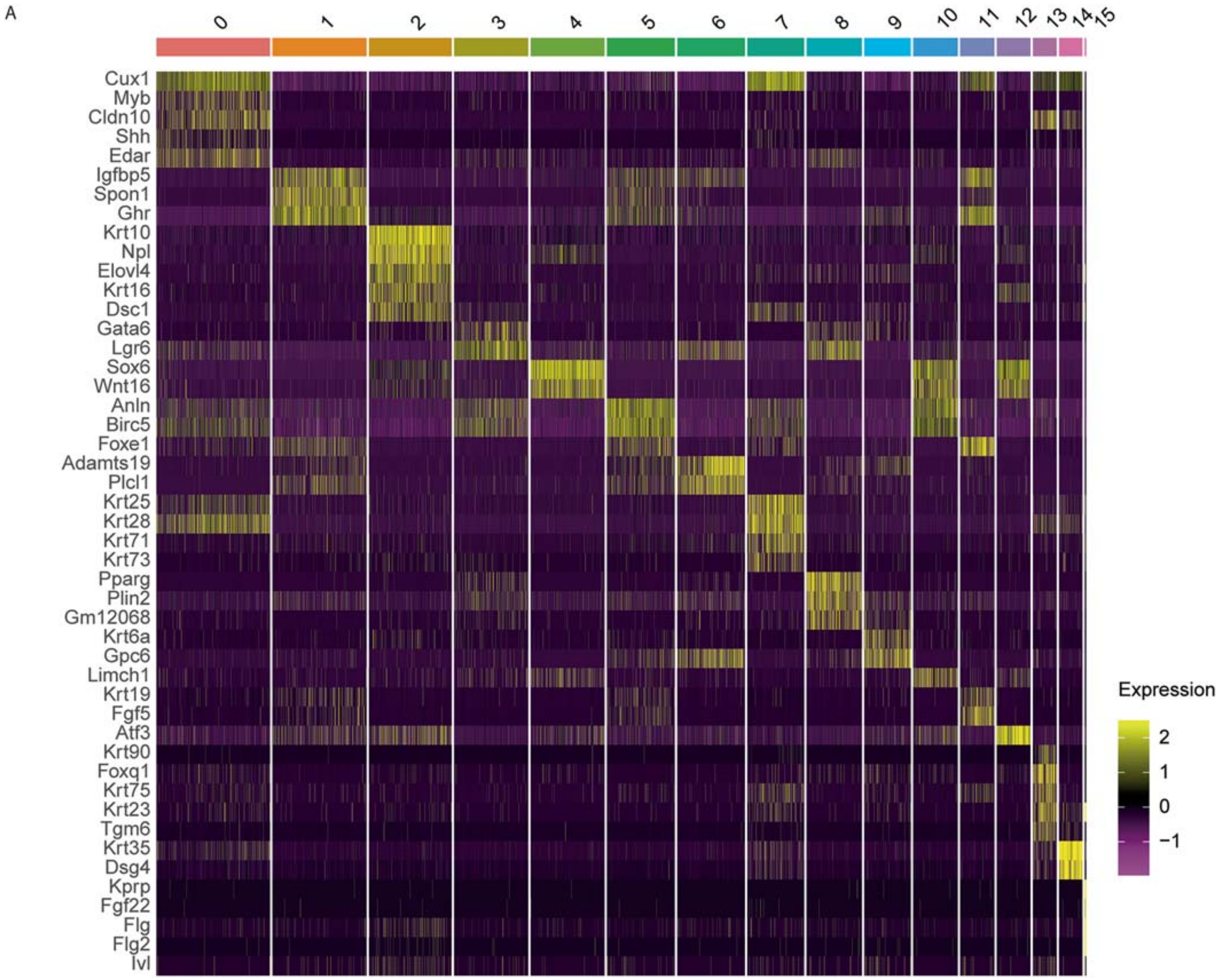

Figure EV4. Marker expression in keratinocyte clusters.

(A) Heatmap showing the marker expression defining each cluster. Colours represent scaled expression by gene.

 © The Author(s)

A

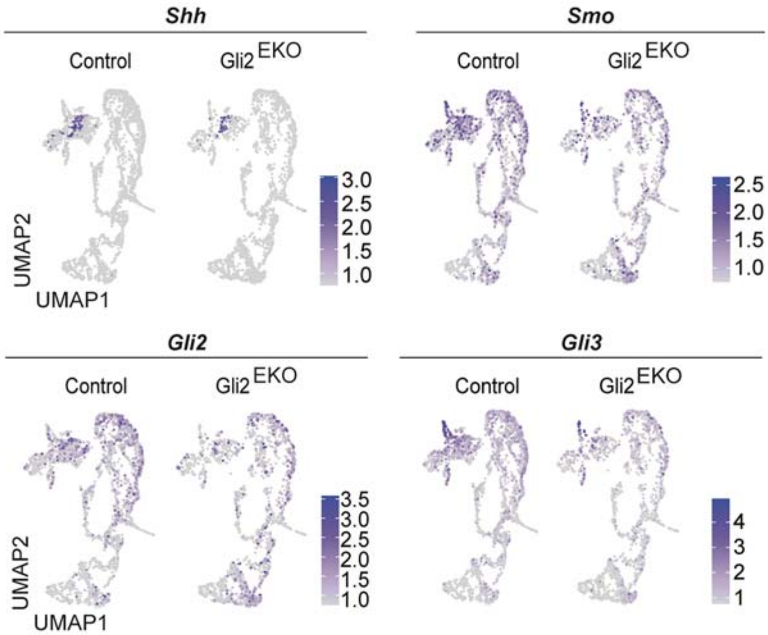

B

Cluster	Control		Gli2^{EKO}	
	# of cells	% of cells	# of cells	% of cells
0	491	15.83	181	8.26
7	229	7.38	108	4.93
13	108	3.48	33	1.51
14	93	3	43	2.05
8	214	6.9	113	5.16

C

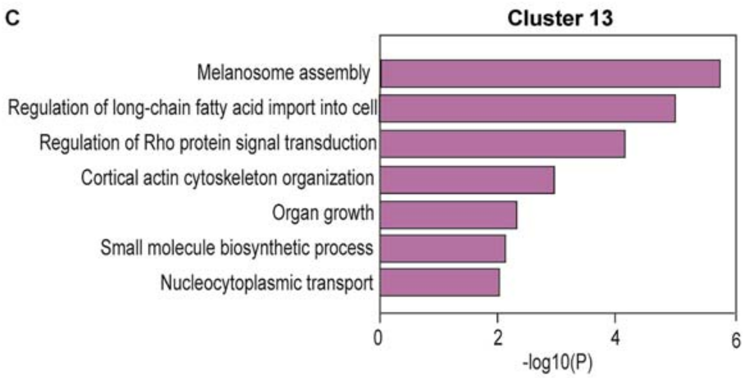

D

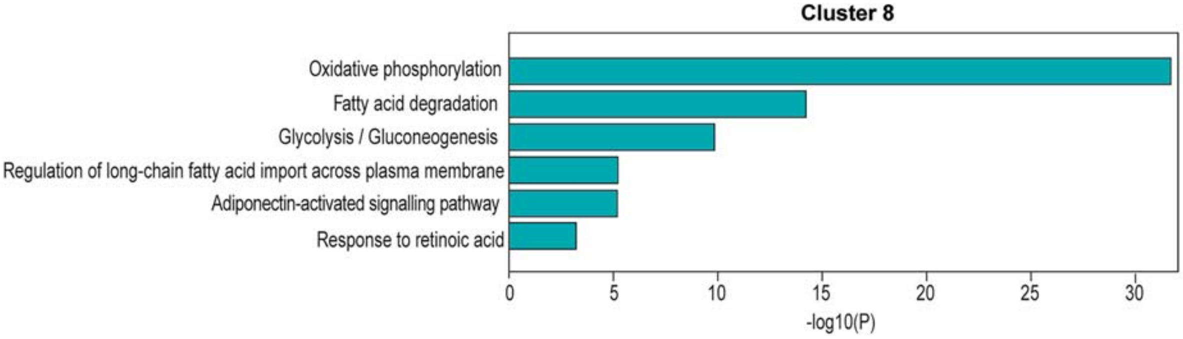

Figure EV5. Molecular and cellular characterisation of distinct keratinocyte populations in Gli2^{EKO} mice.

(**A**) Feature plots of scRNAseq data showing expression of Shh, Smo, Gli2, and Gli3 in Control vs Gli2^{EKO} keratinocytes. Expression levels are colour coded, and expression is shown for values greater than 0.5. (**B**) Numbers and percentages of keratinocytes for clusters 0, 7, 13, 14 and 8 in control vs. Gli2^{EKO} scRNAseq dataset. (**C**, **D**) Gene ontology for pathway and process enrichment analysis of the top 110–130 down- and up-regulated genes in clusters 13 (**C**) and 8 (**D**), respectively. *P* values were calculated based on the cumulative hypergeometric distribution.

© The Author(s)
